# Cell Plasticity-Related Phenotypes and Taxanes Resistance in Castration-Resistant Prostate Cancer

**DOI:** 10.3389/fonc.2020.594023

**Published:** 2020-11-02

**Authors:** Natalia Jiménez, Òscar Reig, Ruth Montalbo, Maria Milà-Guasch, Lluis Nadal-Dieste, Giancarlo Castellano, Juan José Lozano, Iván Victoria, Albert Font, Alejo Rodriguez-Vida, Joan Carles, Cristina Suárez, Montserrat Domènech, Núria Sala-González, Pedro Luis Fernández, Leonardo Rodríguez-Carunchio, Sherley Díaz, Aleix Prat, Mercedes Marín-Aguilera, Begoña Mellado

**Affiliations:** ^1^ Translational Genomics and Targeted Therapeutics in Solid Tumors Lab, Institut d'Investigacions Biomèdiques August Pi i Sunyer (IDIBAPS), Barcelona, Spain; ^2^ Translational Genomics and Targeted Therapeutics in Solid Tumors Lab, Fundació Clínic per a la Recerca Biomèdica, Barcelona, Spain; ^3^ Medical Oncology Department, Hospital Clínic, Barcelona, Spain; ^4^ Genomic Unit, Institut d'Investigacions Biomèdiques August Pi i Sunyer (IDIBAPS), Barcelona, Spain; ^5^ Bioinformatics Platform, Centro de Investigación Biomédica en Red de Enfermedades Hepáticas y Digestivas (CIBEREHD), Barcelona, Spain; ^6^ Medical Oncology Department, Institut Català d'Oncologia, Hospital Germans Trias i Pujol, Badalona, Spain; ^7^ Medical Oncology Department, Institut Hospital del Mar d’Investigacions Mèdiques (IMIM), Hospital del Mar, Barcelona, Spain; ^8^ Vall d’Hebron Institute of Oncology, Vall d’Hebron University Hospital, Barcelona, Spain; ^9^ Medical Oncology Department, Fundació Althaia Manresa, Barcelona, Spain; ^10^ Oncology Department, Institut Català d’Oncologia, Hospital Universitari Doctor Josep Trueta, Girona, Spain; ^11^ Department of Pathology, Hospital Germans Trias i Pujol, IGTP and Universidad Autonoma de Barcelona, Badalona, Spain; ^12^ Department of Pathology, Hospital Clínic, Barcelona, Spain; ^13^ Department of Medicine, University of Barcelona, Barcelona, Spain

**Keywords:** cell plasticity, EMT—epithelial-mesenchymal transition, neuroendocrine, castration-resistant prostate cancer, docetaxel, cabazitaxel, taxanes resistance

## Abstract

The prostatic tumor cells plasticity is involved in resistance to hormone-therapy, allowing these cells to survive despite androgen receptor inhibition. However, its role in taxanes resistance has not been fully established. Gene expression of plasticity-related phenotypes such as epithelial-mesenchymal transition (EMT), stem cell-like and neuroendocrine (NE) phenotypes was studied *in vitro*, *in silico*, in circulating tumor cells (CTCs) (*N*=22) and in tumor samples (*N*=117) from taxanes-treated metastatic castration-resistant prostate cancer (mCRPC) patients. Docetaxel (D)-resistant cells presented a more pronounced EMT phenotype than cabazitaxel (CZ)-resistant cells. *In silico* analysis revealed *ESRP1* down-regulation in taxane-exposed mCRPC samples. Cell plasticity-related changes occurred in CTCs after taxanes treatment. Tumor EMT phenotype was associated with lower PSA progression-free survival (PFS) to D (*P*<0.001), and better to CZ (*P*=0.002). High *ESRP1* expression was independently associated with longer PSA-PFS (*P*<0.001) and radiologic-PFS (*P*=0.001) in D and shorter PSA-PFS in the CZ cohort (*P*=0.041). High *SYP* expression was independently associated with lower PSA-PFS in D (*P*=0.003) and overall survival (OS) in CZ (*P*=0.002), and high *EZH2* expression was associated with adverse OS in D-treated patients (*P*=0.013). In conclusion, EMT profile in primary tumor is differentially associated with D or CZ benefit and NE dedifferentiation correlates with adverse taxanes clinical outcome.

## Introduction

Cell plasticity refers to the ability of cancer cells to switch their phenotype in response to environmental conditions, facilitating therapy fail. Specifically in prostate cancer (PC) cell plasticity allows cancer cells to reprogram and survive despite androgen receptor (AR) inhibition, being considered one of the mechanisms involved in androgen deprivation therapy (ADT) resistance (1). Throughout this process, tumor cells may develop epithelial-mesenchymal transition (EMT), and/or evolve towards an stem cell-like (SCL) or neuroendocrine (NE) phenotypes, and it is possible that they acquire mixed or intermediate phenotypes (2, 3).

ADT, novel hormone-therapies consisting in AR signaling inhibitors (ARSI; abiraterone or enzalutamide), and taxanes, chemotherapy agents that act by blocking microtubules depolymerization, are the most used therapies in metastatic PC (4–8). Today, a high percentage of patients with disseminated PC receive novel hormone-therapies at early stages of disease: in combination with ADT in non-castrate PC, in non-metastatic castration-resistant PC or as first-line treatment for metastatic castration-resistant PC (mCRPC). Taxanes are used after ARSI progression but its clinical activity may be eventually compromised by the development of cell plasticity-related phenotypes (9, 10). Furthermore, different degrees of NE dedifferentiation (from pure, mixed or intermediate adenocarcinoma/NE phenotypes) have been observed in biopsies from mCRPC patients who progressed to abiraterone and its presence is associated with an adverse clinical outcome (11). Moreover, no specific clinical strategies are defined based on the presence of these phenotypes and no targeted therapies have demonstrated benefit in mCRPC patients.

On the other hand, although there is growing evidence that tumor cell plasticity is a relevant biological event involved in therapeutic resistance and aggressive evolution of PC, the presence of these cell plasticity related phenotypes already in the primary tumor and its impact in the therapeutic response and clinical outcome has not been fully described.

In the present study we investigated the presence and potential role in predicting tumor evolution of EMT, SCL and NE cell plasticity-related phenotypes in CRPC cell lines, *in silico*, in circulating tumor cells (CTCs) and in tumor biopsies from mCRPC taxanes-treated patients.

## Materials and Methods

### Establishment of Taxane-Resistant Cells

DU-145 and PC-3 cells were purchased from American Type Culture Collection (ATCC). They were converted to docetaxel-resistant (DU-145DR and PC-3DR, respectively) and to cabazitaxel-resistant cells (DU-145CZR and PC-3CZR, respectively) by exposing them to an initial dose of 1.3 nM of docetaxel (D) and 0.6 nM of cabazitaxel (CZ), respectively, and culturing surviving cells with stepwise increasing doses in an intermittent regimen until a concentration of 6 nM during one year approximately. A subset of parental cells was cultured alongside the resistant ones as a control. All cell lines were authenticated using Human 9-Marker STR Profile and Interspecies Contamination Test by IDEXX BioAnalytics.

DU-145 and PC-3 cell lines were cultured in RPMI 1640 medium (Gibco, Life Technologies, Paisley, UK) and in F-12K nutrient mixture medium (Gibco), respectively, both supplemented with 10% fetal bovine serum (FBS). D and CZ drugs (MedChem Express, Monmouth Junction, NJ, USA) were dissolved in 10 mM in DMSO. D and CZ-resistant cells were maintained in 2 nM of D or CZ-containing medium, respectively.

### Cell Viability Assays 

Cells were seeded at a density of 3,000 cells/well in a 96-well microtiter plate. After 24 h, cells were exposed to D or CZ (MedChem Express, Monmouth Junction, NJ, USA) for an additional 72 h. Cell viability was assessed by using Cell Titer 96 Aqueous One Solution Cell Proliferation Assay kit (Promega, Madison, WI, USA) according to instructions protocol.

### Cell Adhesion and Proliferation Measurement

Quantitative monitoring of cell adhesion and proliferation was performed using the xCELLigence real-time cell analysis (RTCA) system, which measures the change in electrical impedance expressed as the Cell Index (CI). Experiments were carried out in a RTCA DP Instrument (ACEA Bio-sciences, San Diego, CA, USA) placed in a humidified incubator maintained at 37 °C with 5% CO2 according to manufacturer instructions. Briefly, 50 µl of cell-free medium with 10% FBS was added to the wells of E-16 plates for background impedance detection, then 5,000 cells/well were seeded. Cell Index was monitored for 90 h, the first 4 h every 1 min, then once every 30 min. Monitoring of the cells over the first hours provides adhesion data, while over the subsequent hours provides proliferation data. Each cell line was tested in triplicate. The rate of adhesion and proliferation were determined by calculating the averaged slopes of the curves between two given time points. Data were analyzed by RTCA 2.0 software (ACEA Bio-sciences).

### Cell Migration Assay

Cell migration was performed using the Cultrex 96 Well Cell Migration Assay (Trevigen, Gaithersburg, MD, USA) for 24 h and quantified using Calcein-AM according to the manufacturer’s instructions. Each assay was performed in sextuplicate and the experiment was repeated twice, independently.

### Western Blot Analysis

Whole-cell extracts were prepared and Western blot analysis performed as described previously (12). Nitrocellulose membranes were blocked, incubated and washed following the Odyssey System (Li-Cor Biosciences, Lincoln, NE, USA) recommendations. Blots were scanned with an Odyssey Infrared Imaging System (Li-Cor Biosciences).

Antibodies used were Aurora A (D3E4Q) (AURKA) (ref. 14475), AXL (C89E7) (ref. 8661), CD44 (156-3C11) (ref. 3570), E-cadherin (CDH1) (ref. 4065), N-cadherin (CDH2) (ref. 4061), N-MYC (D4B2Y) (ref. 51705), Synaptophysin (D35Ea) (SYP) (ref. 5461), Vimentin (R28) (VIM) (ref. 3932), β-Catenin (6B3) (CTNNB1) (ref. 9582) purchased from Cell-Signaling Technology (Leiden, The Netherlands). Actin (ref. A2066), CHGA (ref. AMAb90525), ESRP1 (ref. HPA023719), and Monoclonal Anti-α-Tubulin clone B-5-1-2 (TUB) (ref. T5168) were purchased from Sigma-Aldrich (St. Louis, MO, USA). ZEB1 (H-102) antibody (ref. sc-25388) was purchased from Santa Cruz Biotechnology (Santa Cruz, CA, USA).

### RNA Extraction

Total RNA from cell lines and CTCs samples was isolated using the Trizol Reagent (Tri-reagent solution; Invitrogen) according to the manufacturer’s protocol. For microarray hybridizations, RNA from cell lines was purified (from aqueous phase of Trizol lysis) using RNeasy Micro kit (QIAGEN, Hilden, Germany). In case of formalin-fixed paraffin-embedded (FFPE) tumor samples, total RNA was isolated using RecoverAll Total Nucleic Acid Isolation Kit (Invitrogen) according to the manufacturer's instructions. RNA was quantified by NanoDrop Spectrophotometer ND-1000 (Thermo Scientific, Wilmington, MA, USA) and RNA quality for microarrays was assessed using a Bioanalyzer 2100 (Agilent, Santa Clara, CA).

### Gene Expression Analysis

One μg of total RNA from cell lines and 0.5 μg from tissue biopsies and CTCs was reverse transcribed using the High Capacity cDNA Archive Kit (Life Technologies), following manufacturer’s instructions. In case of tissues and CTCs samples, cDNA samples were pre-amplified using TaqMan PreAmp Master Mix kit following manufacturer's instructions (Applied Biosystems, Foster City, CA, USA), except that the final reaction volume of the reaction was 12.5 μl.

Real-time quantitative reverse-transcription PCR (qRT-PCR) was performed in duplicate using a StepOne Plus Real-Time PCR system (Applied Biosystems), according to the manufacturer’s recommendations. Data were acquired using Step One Software v2.2.2. Expression values were based on the quantification cycle (Cq) from target genes relative to the Cq of the housekeeping genes *GUSB* in cell lines and tissue samples and *ACTB* in CTCs (DCq). Minus DCq values were considered as the expression level in tissue samples. Relative expression with respect to each reference group studied was reported as fold change or log2ratio. Target genes were amplified using commercial primers and probes (Applied Biosystems): *ACTB* (Hs99999903_m1), *AURKA* (Hs01582073_m1), *AXL* (Hs01064444_m1), *CD44* (Hs01075861_m1), *CDH1* (Hs01023895_m1), *CDH2* (Hs00169953_m1), *CHGA* (Hs00900375_m1), *CTNBB1* (Hs00170025_m1), *ESRP1* (Hs00214472_m1), *EZH2* (Hs00544830_m1), *GUSB* (Hs99999908_m1), *MYCN* (Hs00232074_m1), *SYP* (Hs00300531_m1), *TC2N* (Hs01120134_m1), *TWIST1* (Hs01675818_s1), *VIM* (Hs00185584_m1), *ZEB1* (Hs01566407_m1).

### Microarrays Hybridization and Differential Expression Analysis

RNA samples from parental and resistant cell lines were processed using WT PLUS chemistry (Affymetrix, Santa Clara, CA, USA). Fragmented and labelled ss-cDNA was prepared according to Affymetrix WT PLUS Reagent Kit user guide. Following fragmentation and terminal labelling, 5.2 µg of ss-cDNA were hybridized for 16 h at 45 °C on GeneChip Human Gene 2.0 ST Arrays (Affymetrix, Santa Clara, CA, USA). Arrays were washed and stained in the Affymetrix Fluidics Station 450. GeneChips were scanned using the Affymetrix GeneCHip Scanner 3000 System.

Raw expression data from microarrays were normalized using the robust multiarray algorithm (13) with a custom probe set definition that mapped probes to Entrez Gene IDs (hugene20st_Hs_ENTREZG) (14). After this step, a filtering was done to obtain the 5% genes with the lowest coefficient of variation. Finally, to identify differentially expressed genes between the different microarray study groups, we employed LIMMA (15) to estimate moderated t-statistics and to select statistically differentially expressed genes.

Significant genes were selected if they accomplished a fold change (|FC|)≥1.5 and false discovery rate (FDR)<0.05. Differentially expressed genes of resistant versus parental cells were mapped against the Kyoto Encyclopedia of Genes and Genomes (KEGG) pathway database (16) and analysis was performed using clusterProfiler (v3.8.1) R package.

Gene interactions were studied using the Ingenuity Pathway Analysis (IPA) software (QIAGEN).

We defined signatures for epithelial-mesenchymal transition (EMT) (345 EMT-related genes from the dbEMT database (17) plus *ESRP1*), stem cell-like (SCL) [659 genes from five expression profiles (18–21) using StemChecker online (22)] and neuroendocrine (NE) prostate cancer (NEPC) [70 NE-related genes from the integrated NEPC score (23) plus *ASCL1*, *AURKB*, *CHGA*, and *SYP* (24)], and tested them *in silico* in our microarray data. Hierarchical cluster analysis of these signatures was performed using Cluster 3.0 (25) and results were visualized in Java TreeView (26).

### Circulating Tumor Cells Enrichment

Blood circulating tumor cells (CTCs) enrichment was performed using the IsoFlux System (Fluxion Biosciences, South San Francisco, CA, USA). Two 10 mL EDTA tubes were collected from patients before taxanes initiation and after three cycles or at progression. One of the tubes was used for CTCs counting with the IsoFlux CTC Enumeration Kit, according to manufacturer’s instructions. CTCs were defined as nucleated cells, morphologically intact, cytokeratin positive and CD45 negative cells. The second EDTA tube was used for gene expression analysis. Briefly, peripheral blood mononuclear cells were isolated by Ficoll (GE Healthcare, Uppsala, Sweden) gradient and IsoFlux Rare Cell Enrichment Kit was used to incubate them with customized anti-epithelial cell adhesion molecule (EPCAM), prostate stem cell antigen (PSCA) and N-cadherin (CDH2)-coated beads for 2 h at 4 °C. Cell-beads complexes were loaded into Isoflux cartridges to be isolated automatically by the instrument. Cells returned by the instrument were stained or total RNA was extracted.

### *In Silico* Analysis in Prostate Cancer 

The alteration status of genes involved in EMT, SCL, and NE cell plasticity related phenotypes was studied in an *in silico* analysis including seven PC studies summing a total of 1,131 samples (23, 27–32) obtained from cBioportal for Cancer Genomics platform (http://cbioportal.org) (33, 34).

RNA-seq data from Abida et al. (35) were also downloaded from cBioportal and analyzed. Boxplots representing log FPKM (Fragments Per Kilobase of exon per Million fragments mapped) were generated by R (v.3.6.3) software.

### Patients and Samples 

We retrospectively collected tumor samples from patients diagnosed with mCRPC treated with D (75 mg/m^2^ iv every 3 weeks) or CZ (25 mg/m^2^ iv every 3 weeks or 10 mg/m^2^ iv weekly), both in association with prednisone (10 mg/day p.o), with available FFPE tumor tissue from several institutions.

Patients under the same treatment regimen with the possibility of extracting CTCs samples were prospectively included as a part of an ongoing prospective biomarker study in our institution. Blood samples were extracted before starting treatment and after 3 cycles and/or at time of progression

Treatment-response criteria and progressive-disease definitions followed Prostate Cancer Working Group 2 criteria (36).

### Statistical Analysis 

*In vitro* experiments data were expressed as mean ± SD and analyzed by Student t-test. All tests were 2-sided and *P*-values <0.05 were considered significant. Optimal cut-offs for gene expression were assessed using maximally selected log-rank statistics (Maxstat package) (37). PSA progression-free survival (PFS), radiologic-PFS (RX-PFS), and overall survival (OS) were calculated from the date of taxanes initiation to PSA progression, radiologic progression, and death or last follow-up visit, respectively, and were evaluated by log-rank test. Univariate analysis of gene expression levels and other clinical variables was performed by Cox regression; *P*<0.1 was required for inclusion in multivariate analysis. Test of interaction was performed by entering into proportional hazard models selected multiplicative interaction terms between two binary variables: treatment (D or CZ) and *ESRP1* expression (high or low). Changes in gene expression in CTCs samples before and after treatment were analyzed by Wilcoxon signed-rank test. SPSS 12.0 and R (v.3.6.3) softwares were used for statistical analyses.

## Results

### Phenotypic Characterization of Docetaxel and Cabazitaxel-Resistant Cells

**Figure 1:**
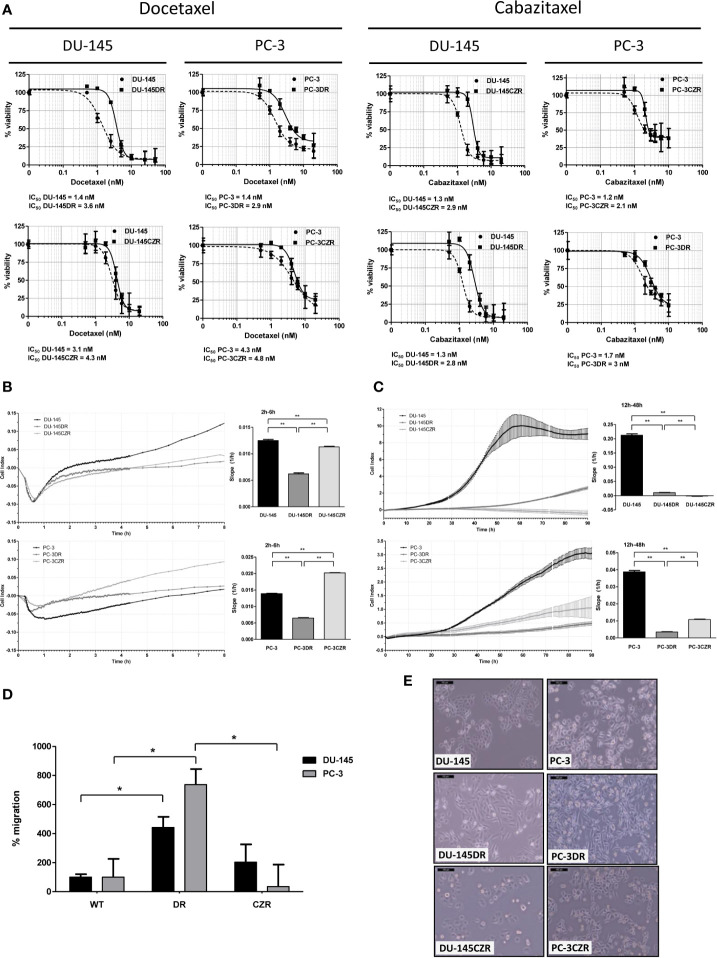
Taxane-resistant cells characterization. **(A)** Cell-viability assays (MTS) of docetaxel (D) and cabazitaxel (CZ)-treatment in D-resistant (DR) and CZ-resistant (CZR) cells and their respective parental cells (DU-145 and PC-3). IC_50_ value of each cell line from these MTS assays is indicated. Results are represented as mean of % viability ± SD. Each treatment was carried out in triplicate for 72 h. **(B)** Cell adhesion curves of parental and resistant cells. Data is plotted as mean of Cell Index. Adhesion rates (bar-graphs) were determined by analyzing the slope of the lines between the 2 h and 6 h interval. **(C)** Cell proliferation curves of parental and resistant cells. Data is plotted as mean of Cell Index ± SD. Proliferation rates (bar-graphs) were determined by analyzing the slope of the lines between the 12 h and 24 h interval. **(D)** Migration rate quantification of DR and CZR cells versus their respective parental cells. Results are represented as mean of % migration ± SD. Significant differences are indicated as *P < 0.05 and **P < 0.001 (Student t-test). **(E)** Light microscopy images (100X) showing the morphology of DR and CZR cells (cultured with 2 nM of D or CZ, respectively) and their respective parental cells.

Docetaxel (D) and cabazitaxel (CZ) resistant cell lines (DR and CZR, respectively) were generated from CRPC cell line models DU-145 and PC-3. DU-145DR and PC-3DR cells acquired levels of resistance to D that were 1.5 to 2.1 times higher than their parental cells. The levels of resistance to CZ for DU-145CZR and PC-3CZR were 2.2 to 4.5 times higher than their parental cells (**Figure 1A**). DR and CZR cells were also resistant to CZ and D, respectively, suggesting the existence of a cross resistance between both agents. Phenotypically, DR differed from CZR cells when compared to parental cells: DR cells were more elongated, with lower adhesion and proliferation, and higher migration rates, while CZR cells were more rounded and similar to parental cells, with similar (DU-145 model) or higher (PC-3 model) adhesion and lower proliferation rates, and without significant differences in migration rate (**Figures 1B–E**). These patterns suggest that DR cells have more motility abilities than CZR cells.

### Differentially Expressed Genes in Taxane-Resistant Versus Parental Cell Lines

**Figure 2:**
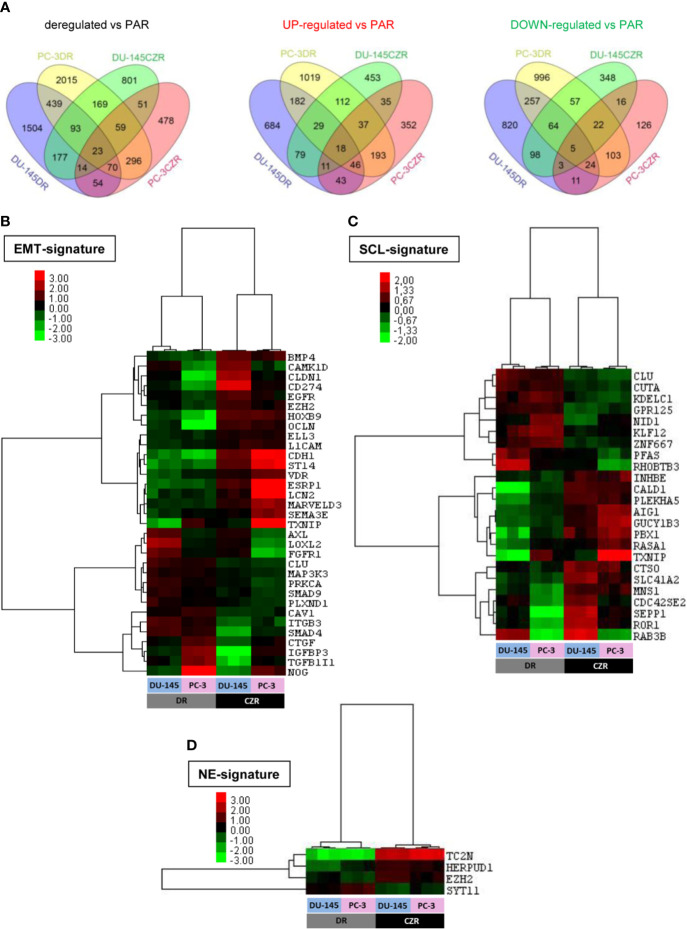
Differentially expressed genes in docetaxel-resistant (DR) and cabazitaxel-resistant (CZR) cell lines from microarray data. **(A)** Venn Diagram of the differentially expressed genes between DR and CZR cell lines versus their respective parental cells (PAR). **(B)** Hierarchical clustering expression heatmap for expression values of the epithelial-mesenchymal transition (EMT)-signature in DR and CZR cell lines. **(C)** Hierarchical clustering expression heatmap for expression values of the neuroendocrine (NE)-signature in DR and CZR cell lines. **(D)** Hierarchical clustering expression heatmap for expression values of the NE-signature in DR and CZR cell lines.

We performed cDNA microarrays to study the gene expression changes related to taxanes-acquired resistance in the above characterized cell lines. The microarray data analysis revealed 625 and 147 differentially deregulated genes in DR and CZR versus parental cells (|FC|≥1.5 and FDR<0.05), respectively (**Figure 2A** and **Tables S1** and **S2**). KEGG pathway enrichment and Ingenuity network analysis showed the relevance of EMT and NFKβ pathway in both D and CZ-resistance, among other deregulated pathways. Of note, *ESRP1* appeared highly underexpressed in DR models (**Figure S1A–C**).

Twenty-three genes were commonly deregulated in both DR and CZR versus parental cells (|FC|≥1.5 and FDR<0.05) (**Figure 2A** and **Table S3**). Among them, *ZEB1* and *ITGB3* were up-regulated and *BMP4* was down-regulated. Ingenuity network analysis showed that the most relevant network had two significant nodes around *EGFR* and *ITGB3* genes and the EMT-related markers, *ZEB1* and *TRPC1*, appeared overexpressed (**Figure S1D**).

### Differential Expression Analysis in CZ Versus D-Resistant Cells 

From the microarray data analysis we found 559 genes significantly commonly deregulated in CZR models versus DR models (|FC|≥1.5 and FDR<0.05) (**Table S4**). *ESRP1*, *TC2N*, *MPZL2, CDH1, OCLN*, and *EPCAM* were epithelial-related genes overexpressed in CZR respect to DR cell lines, whereas *AXL* was down-regulated. Ingenuity network analysis centered the most relevant network on *CDH1* and *EZH2* (**Figure S1E**).

These results were validated by qRT-PCR and Western Blot. By these techniques it was observed an overall increased expression of mesenchymal markers such as ZEB1 and VIM and a reduced expression of epithelial markers CDH1 and ESRP1 lead by taxanes. This validation supports a more pronounced EMT profile in DR cells (**Figure S2**).

We defined, based on literature and published gene expression data (as described in Materials and Methods), EMT, SCL, and NE-signatures and tested them *in silico* in our microarray data. We found 33 EMT, 24 SCL and 4 NE-related genes differentially expressed in CZR versus DR cells (|FC|≥1.5 and FDR<0.05). Hierarchical cluster analysis revealed a different deregulation pattern of EMT (more pronounced in DR cells) and SCL-related genes between D and CZ-resistance and more expression of NE markers in CZR than in DR cells (**Figures 2B–D**).

### Taxanes Dose-Response Experiments 

In order to evaluate whether EMT and NE patterns were modifiable in a dose-dependent manner we treated cell lines with increasing doses of taxanes and evaluated EMT and NE gene expression by qRT-PCR. We observed that D and CZ-treatment induced changes in the expression of EMT and NE markers at different degrees. Notably, CZ exposure significantly increased *ZEB1* and *VIM* expression in PC-3 models in a dose-dependent manner, restored *ESRP1* expression in DU-145DR model and decreased *AXL* levels in DR models but not in CZR cells (**Figure S3**). D and CZ-treatment also increased the expression of NE markers *SYP*, *MYCN* (only detected in PC-3 model), and *CHGA* in a dose-response manner in both resistant models (**Figure S4**). All together these results confirm the plasticity towards EMT and NE patterns after a short-term drug exposure *in vitro*.

### *In Silico* Analysis of Cell Plasticity-Related Genes in Prostate Cancer

**Figure 3:**
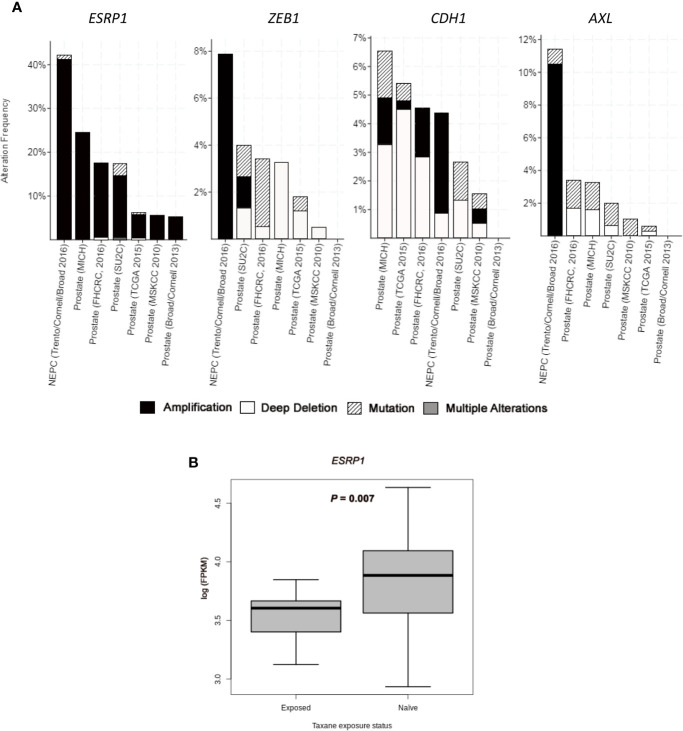
*In silico* analysis from public datasets. **(A)** Bar graphs representing alterations’ frequency of cell plasticity related genes in prostate cancer from seven studies (23,27–32) through cBio Cancer Genomics Portal (http://cbioportal.org) platform. **(B)** Boxplot representing *ESRP1* expression (log FPKM (Fragments Per Kilobase of exon per Million fragments mapped)) from RNA-seq data in taxane-naïve versus taxane-exposed mCRPC tumor samples from ARSI-naïve cohort of Abida et al. (35). Welch’s *t*-test was used for means comparison.

A subgroup of cell plasticity related genes was selected among those significantly deregulated between CZR and DR cell lines (and validated by qRT-PCR and Western Blot) to be analyzed *in silico* in seven PC studies obtained from cBioportal: three studies of primary tumors with 333 (30), 240 (31), and 57 samples (32); three studies of metastatic PC with 61 (27), 176 (28), and 150 samples (29); and one study of NEPC with 114 samples (23). *ESRP1* gene, one of the most differentially deregulated genes between DR and CZR, was mostly amplified, being this alteration detected in around 5% of primary tumors, 20% of metastatic PC, and in 40% of NEPC. Amplification of *ZEB1* (7.9%) and *AXL* (10.5%), both found in NEPC, were also frequent alterations. Otherwise, *CDH1* showed a variety of alterations, but most notably was amplified in NEPC (3.5%) and deleted in primary tumors and in metastatic PC (0.5%–4.5%) (**Figure 3A** and **Table S5**).

We also investigated gene expression changes in tumors from mCRPC patients treated with taxanes, by using RNA-seq data from the study of Abida et al. (35) Within ARSI-naïve patients, *ESRP1* expression was significantly lower in taxane-exposed respect to taxane-naïve patients (*P*=0.007) (**Figure 3B**). Median expression value for *AXL*, *VIM*, *ZEB1*, *AURKA*, *EZH2*, *MYCN*, and *SYP* was higher and for *CDH1* was lower in taxane-exposed versus taxane-naïve patients, although these differences were not statistically significant (**Figure S5**). These results confirm the effect of taxanes in the *ESRP1* down-regulation and EMT up-regulation in a clinical sample setting.

### Cell Plasticity in CTCs

**Figure 4:**
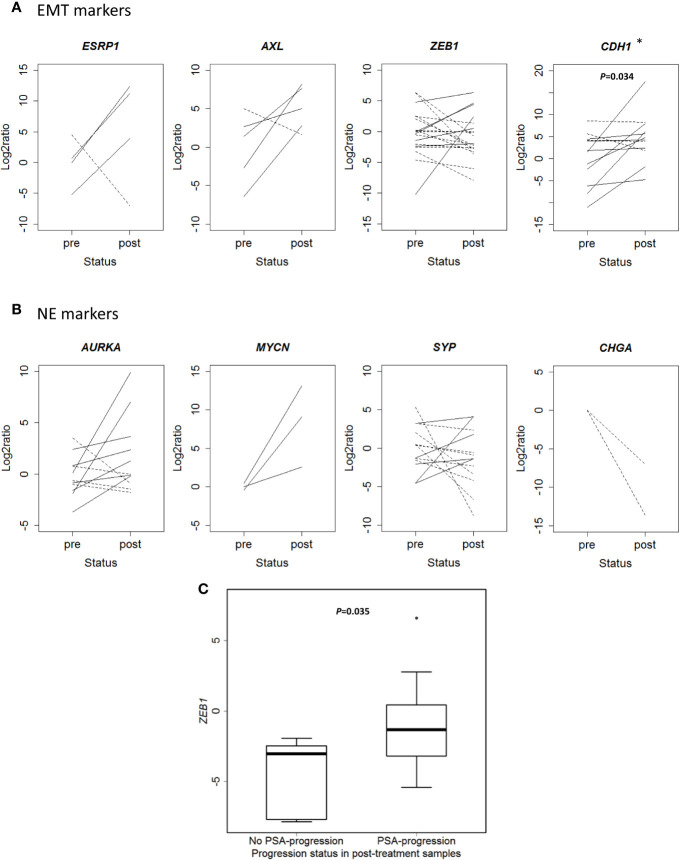
Changes in gene expression markers in circulating tumor cells (CTCs) samples after taxanes treatment by qRT-PCR. **(A)** Epithelial-mesenchymal transition (EMT) markers expression levels (represented as Log2ratio) before (pre) and after (post) taxanes treatment in CTCs. **(B)** Neuroendocrine (NE) markers expression levels (represented as Log2ratio) before (pre) and after (post) taxanes treatment in CTCs. Solid lines: overexpression; Dashed lines: underexpression. **(C)** Boxplot representing *ZEB1* expression levels (minus DCq) according to PSA-progression status in post-treatment samples. *P < 0.05.

Beyond the *in silico* analysis, we wondered whether molecular plasticity changes could be observed in CTCs samples. Then we collected 47 CTCs-enriched blood samples from 22 mCRPC patients, 17 of them with PSA-progression at post-treatment extraction, and performed qRT-PCR analyses. Patients’ characteristics’ are shown in **Table 1**. Gene expression patterns in CTCs before and after taxanes treatment showed changes in expression levels of all EMT and NE markers, with high intra- and inter-patient heterogeneity (**Figures 4A, B** and **Figure S6**). *CDH1* expression was significantly higher in post-treatment samples (*P*=0.034). Response was not correlated with pre-treatment expression values or with the difference obtained from post- and pre-treatment data. However, *ZEB1* expression after treatment was higher in patients with PSA-progression compared to the non-progressing patients (*P*=0.035) pointing out *ZEB1* as a potential marker of taxanes resistance in CTCs (**Figure 4C**).

**Table 1:** Clinical characteristics of patients with CTCs-enriched blood samples.

	Total	DOCETAXEL	CABAZITAXEL	*P*-value
Patients, N (%)	22*	16 (66.7)	8 (33.3)	
	(*2 included in both D and CZ cohorts, 1 has 1 sample post-D and pre-CZ)			
Age* (years)				
Median (range)	70 (41.6 - 87.1)	70.1 (41.6 - 87.1)	67.9 (43.2 - 83.3)	
Post-treatment samples, *N* (%)				
end of treatment	15 (62.5)	10 (62.5)	5 (62.5)	
after progression	6 (25)	4 (25)	2 (25)	
during treatment	3 (12.5)	2 (12.5)	1 (12.5)	
PSA response in post-treatment samples, *N* (%)				
Stable disease	4 (16.7)	3 (18.8)	1 (12.5)	
Partial response	3 (12.5)	1 (6.3)	2 (25)	
Progression	17 (70.8)	12 (75)	5 (62.5)	
CTCs pre-treatment				
Mean (range)	17 (0 - 100)	23 (1 - 100)	5 (0 - 22)	0.009
CTCs post-treatment				
Mean (range)	21 (0 - 182)	23 (0 - 182)	16 (1 - 44)	0.713
Stage at diagnosis, *N* (%)				
<IV	5 (20.8)	5 (31.3)	0 (0)	0.114
IV	15 (62.5)	8 (50)	7 (87.5)	
NA	4 (16.7)	3 (18.8)	1 (12.5)	
Gleason sum at diagnosis, *N* (%)				
≤7	8 (33.3)	6 (37.5)	2 (25)	0.667
≥8	16 (66.7)	10 (62.5)	6 (75)	
NA				
Best PSA response, *N* (%)				
Stable disease	8 (33.3)	5 (31.3)	3 (37.5)	
Partial response	8 (33.3)	4 (25)	4 (50)	
Progression	6 (25)	6 (37.5)	0 (0)	
NA	2 (8.3)	1 (6.3)	1 (12.5)	
Presence of bone metastases, *N* (%)				
Yes	21 (87.5)	14 (87.5)	7 (87.5)	1
No	3 (12.5)	2 (12.5)	1 (12.5)	
Presence of visceral metastases*, *N* (%)				
Yes	7 (29.2)	6 (37.5)	1 (12.5)	0.352
No	17 (70.8)	10 (62.5)	7 (87.5)	
ECOG performance status score*, *N* (%)				
0	1 (4.2)	1 (6.3)	0 (0)	1
1 or 2	23 (95.8)	15 (93.8)	8 (100)	
Baseline PSA (ng/mL)				
Median (range)	27 (1.8 - 479.6)	26 (2.8 - 479.6)	56 (1.8 - 377.7)	0.738
Baseline haemoglobin concentration (g/L)				
Median (range)	122 (84 - 498)	134 (110 - 498)	109 (84 - 140)	0.003
Baseline alkaline phosphatase (U/L)				
Median (range)	142 (54 - 873)	115 (54 - 344)	210 (76 - 873)	0.032
Baseline lactate dehydrogenase (U/L)				
Median (range)	356 (125 - 949)	346 (125 - 949)	565 (177 - 948)	0.217
A/E treatment pre-taxanes, *N* (%)				
Yes	17 (70.8)	11 (68.8)	6 (75)	1
No	7 (29.2)	5 (31.3)	2 (25)	

P-value is based on Fisher exact test and Mann-Whitney U test for categorical and continuous variables, respectively. N, number of cases; *data at taxanes start time; CTCs, circulating tumour cells; ECOG, Eastern Cooperative Oncology Group; A/E, Abiraterone/Enzalutamide therapy; D, Docetaxel; CZ, Cabazitaxel; PSA, prostate-specific antigen; NA, not available.

### Cell Plasticity-Related Gene Expression in Non-Castrate Tumor Biopsies and Clinical Outcome 

In order to confirm previous results in another cohort of tumor samples, we collected FFPE tumor tissues from taxanes-treated patients from several Spanish institutions and analyzed gene expression by qRT-PCR. A hundred and seventeen patients were included in this study (**Table 2**). For D-treated patients, median follow-up and PSA-PFS were 21.3 (0.9–90.1) and 7.7 (6.1–9.2) months, respectively; and for CZ-treated patients 12 (1.3–64.9) and 3 (1.7–4.3) months, respectively.

**Table 2:** Clinical characteristics of patients with FFPE tissue samples.

	Total	DOCETAXEL	CABAZITAXEL	*P*-value
Patients, *N* (%)	117*	103 (72)*	40 (28)*	
	(*26 (22.2) received both D and CZ)	(*77 (65.8) received only D)	(*14 (12) received only CZ)	
Age* (years)				
Median (range)	69.5 (41.6 - 87.1)	69.4 (41.6 - 87.1)	68.7 (43.2 - 83.3)	
Tumour origin, *N* (%)				
Primary	104 (88.9)	92 (89.3)	37 (92.5)	0.757
Metastatic	13 (11.1)	11 (10.7)	3 (7.5)	
Tumour Hormonal Status, *N* (%)				
HS	93 (79.5)	81 (78.6)	34 (85)	0.485
CRPC	24 (20.5)	22 (21.4)	6 (15)	
Stage at diagnosis, *N* (%)				
<IV	36 (30.8)	33 (32)	12 (30)	0.430
IV	65 (55.6)	54 (52.4)	28 (70)	
NA	16 (13.7)	16 (15.5)	0 (0)	
Gleason sum at diagnosis, *N* (%)				
≤7	37 (31.6)	34 (33)	8 (20)	0.148
≥8	73 (62.4)	63 (61.2)	30 (75)	
NA	7 (6)	6 (5.8)	2 (5)	
Best PSA response, *N* (%)				
Stable disease	39 (33.3)	31 (30.1)	19 (47.5)	
Partial response	58 (49.6)	56 (54.4)	9 (22.5)	
Progression	18 (15.4)	15 (14.6)	11 (27.5)	
NA	2 (1.7)	1 (1)	1 (2.5)	
Metastases at diagnosis, *N* (%)				
Yes	54 (46.2)	44 (42.7)	23 (57.5)	0.564
No	43 (36.8)	39 (37.9)	16 (40)	
NA	20 (17.1)	20 (19.4)	1 (2.5)	
Presence of bone metastases, *N* (%)				
Yes	99 (84.6)	86 (83.5)	35 (87.5)	1
No	9 (7.7)	8 (7.8)	3 (7.5)	
NA	9 (7.7)	9 (8.7)	2 (5)	
Presence of visceral metastases*, *N* (%)				
Yes	39 (33.3)	31 (30.1)	16 (40)	0.325
No	69 (59)	63 (61.2)	22 (55)	
NA	9 (7.7)	9 (8.7)	2 (5)	
ECOG performance status score*, *N* (%)				
0	17 (14.5)	16 (15.5)	2 (5)	0.092
1 or 2	84 (71.8)	72 (69.9)	35 (87.5)	
NA	16 (13.7)	15 (14.6)	3 (7.5)	
Baseline PSA (ng/mL)				
Median (range)	62.8 (0.04 - 1565)	62.8 (0.2 - 922.6)	173.3 (0.04 - 1565)	0.064
Baseline haemoglobin concentration (g/L)				
Median (range)	128 (60 - 166)	128 (67 - 166)	117 (60 - 151)	0.014
Baseline alkaline phosphatase (U/L)				
Median (range)	178 (17 - 3160)	161 (17 - 3160)	225 (65 - 698)	0.073
Baseline lactate dehydrogenase (U/L)				
Median (range)	366 (153 - 1255)	369 (153 - 1255)	436.5 (190 - 1536)	0.008
A/E treatment pre-taxanes, *N* (%)				
Yes	35 (29.9)	28 (27.2)	22 (55)	0.003
No	82 (70.1)	75 (72.8)	18 (45)	

P-value is based on Fisher exact test and Mann-Whitney U test for categorical and continuous variables, respectively. N, number of cases; *data at taxanes start time; ECOG, Eastern Cooperative Oncology Group; A/E, Abiraterone/Enzalutamide therapy; PSA, prostate-specific antigen; D, Docetaxel; CZ, Cabazitaxel; HS, hormone sensitive; CRPC, castration-resistant prostate cancer; NA, not available.

**Figure 5:**
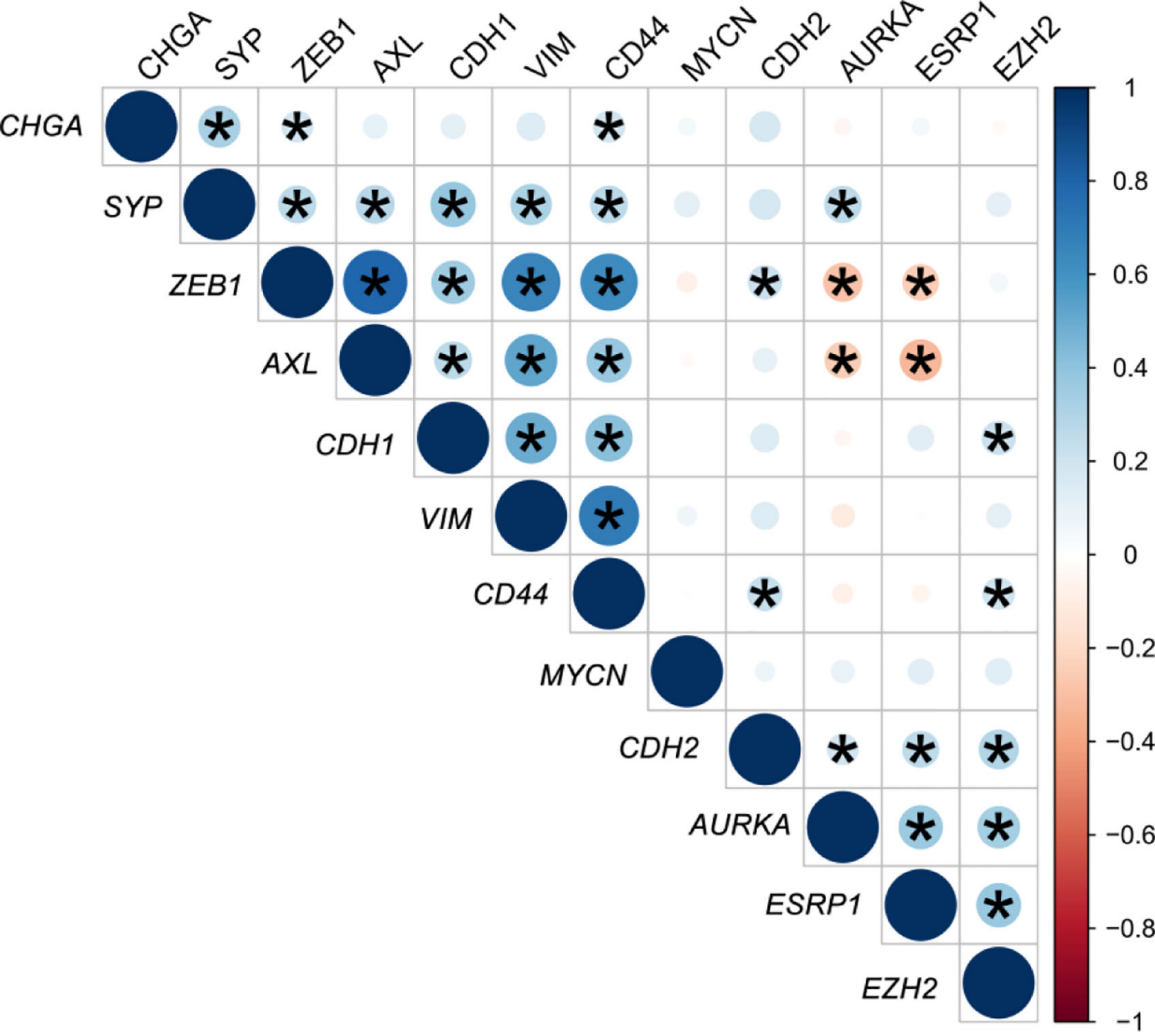
Correlation matrix of epithelial-mesenchymal transition (EMT) and neuroendocrine (NE) cell plasticity related genes in tissue samples. Correlation coefficients (r) between minus DCq values from qRT-PCR data are represented. *P < 0.05.

A gene expression correlation matrix between EMT, SCL, and NE markers (**Figure 5**) showed significant positive correlation between EMT markers (*ZEB1*, *AXL*, *VIM*, and *CDH1*), and these with the SCL-marker *CD44*. Correlation between NE markers was also positive. This fact suggests that EMT, NE and SCL phenotypes may coexist in primary tumors. Significant negative correlation was observed between *ESRP1-ZEB1* and *ESRP1-AXL* (*P*<0.05) confirming the inverse expression pattern between EMT and *ESRP1* expression.

**Figure 6:**
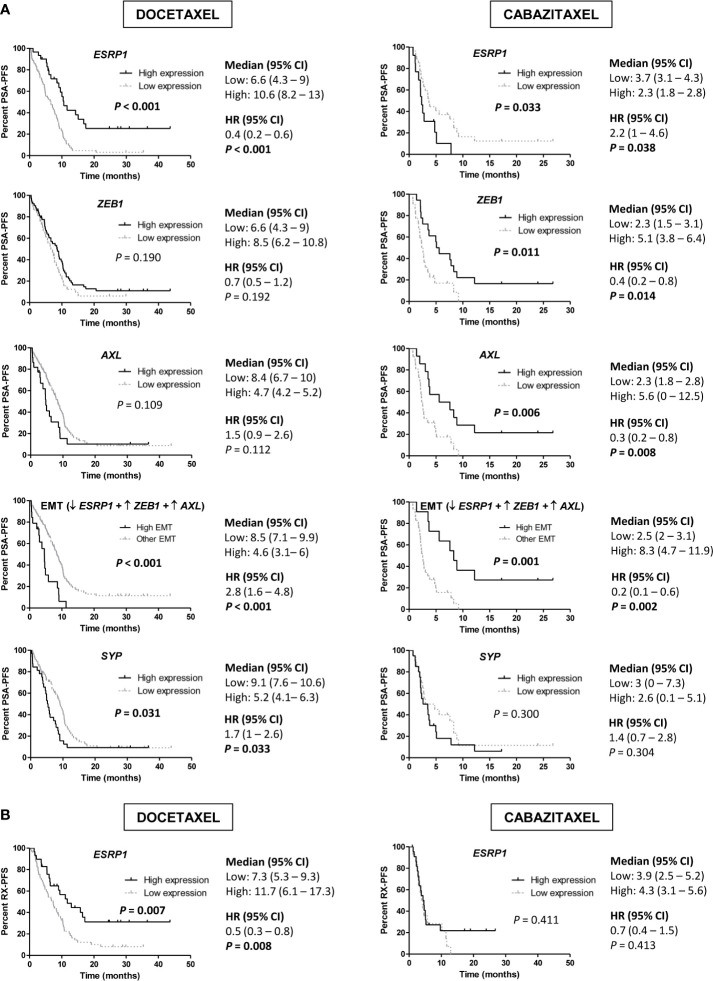
Progression-free survival (PFS) analysis in taxanes-treated patients according to gene expression in tumor samples by qRT-PCR. **(A)** Kaplan-Meier curves representing PSA-PFS according to epithelial-mesenchymal transition (EMT) markers and *SYP* gene expression levels. **(B)** Kaplan-Meier curves representing radiologic-PFS (RX-PFS) according to *ESRP1* expression levels. HR, hazard ratio; CI, confidence interval.

We observed that high expression of *ESRP1* predicted longer PSA-PFS (hazard ratio [HR] 0.4; 95% confidence interval [CI] 0.2–0.6; *P*<0.001) and RX-PFS (HR 0.5; 95 % CI 0.3–0.8; *P*=0.008) to D, but conversely shorter PSA-PFS to CZ (HR 2.2; 95 % CI 1–4.6; *P*=0.038). Moreover, high expression of both *ZEB1* (HR 0.4; 95 % CI 0.2–0.8; *P*=0.014) and *AXL* (HR 0.3; 95 % CI 0.2–0.8; *P*=0.008) was associated with a better PSA-PFS to CZ (**Figure 6**). The association of other EMT markers with PSA-PFS is shown in the **Figure S7**.

Then, we defined a group of patients with high-EMT phenotype (low *ESRP1* and high *ZEB1* and *AXL* expression) and compared it with the rest of patients. High-EMT patients showed a better PSA-PFS to CZ (HR 0.2; 95 % CI 0.1–0.6; *P*=0.002), contrary to D (HR 2.8; 95 % CI 1.6–4.8; *P*<0.001) (**Figure 6A**).

In a multivariate analysis, high *ESRP1* expression was independently associated with longer PSA-PFS (HR 0.3; 95 % CI 0.2–0.6; *P*<0.001) and RX-PFS (HR 0.3; 95 % CI 0.2–0.6; *P*=0.001) in D-treated patients and shorter PSA-PFS in the CZ-cohort (HR 3.1; 95 % CI 1–9.1; *P*=0.041) (**Table S6** and **S7**). Moreover, there was a significant interaction between *ESRP1* expression levels and D or CZ-treatment related to PSA-PFS (*P*<0.001).

Respect to OS, in the univariate analysis some EMT markers such as *ZEB1* for D-treated patients, *AXL* for CZ-treated patients and *VIM* for both were associated with a better OS, but none of them was significant in the multivariate analysis (**Figure S8**).

**Figure 7:**
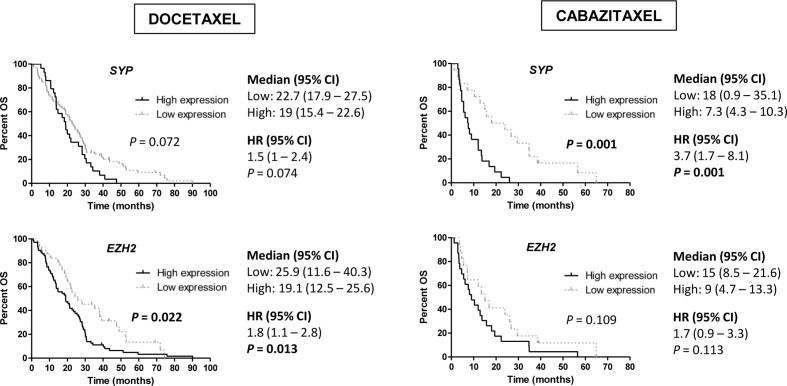
Overall survival (OS) analysis in taxanes-treated patients according to gene expression of neuroendocrine (NE) markers (*SYP* and *EZH2*) in tumor samples by qRT-PCR. HR, hazard ratio; CI, confidence interval.

Regarding NE markers, high expression of *SYP* was associated with shorter PSA-PFS to D (HR 1.7; 95 % CI 1–2.6; *P*=0.033). Moreover, high expression of *EZH2* for D (19.1 vs 25.9 months; HR 1.8; 95 % CI 1.1–2.8; *P*=0.013) and *SYP* for CZ (7.3 vs 18 months; HR 3.7; 95 % CI 1.7–8.1; *P*=0.001) correlated with lower OS (**Figure 7**). In a multivariate analysis, high *SYP* expression was independently associated with shorter PSA-PFS in D-treated patients (HR 2; 95 % CI 1.3–3.3; *P*=0.003) and worse OS in CZ-treated patients (HR 5.3; 95 % CI 1.8–15.4; *P*=0.002) (**Tables S6** and **S8**).

## Discussion

In this study we show that changes in cell plasticity-related phenotypes occur after taxane exposure in *in vitro* models of CRPC, in CRPC biopsies analyzed *in silico*, and in CTCs from patients with mCRPC treated with taxanes. We observed that this plasticity appears with different patterns for D and CZ treatments. Moreover, we found that the expression of EMT and NE markers in primary tumors predicts clinical outcome to taxanes in the mCRPC setting.

The taxanes D and CZ have shown a survival benefit in mCRPC patients (4–8). Specifically, CZ has shown to be active in patients with progression or low response to D (8, 38) and increases survival in patients who have progressed to prior D and abiraterone or enzalutamide (39), as demonstrated in randomized clinical trials. The fact that CZ is active in these adverse clinical situations supports that it may overcome the resistance to D or to hormone-therapy. The activity of CZ observed in our series in tumors with EMT phenotype (as discussed below) may be a potential explanation.

EMT phenotype has been associated by us and others with intrinsic and acquired resistance to D in CRPC (9, 10). Here, we show that DR and CZR cells have several EMT-deregulated genes when compared with parental cells, supporting the relevance of EMT in both D and CZ-resistances (9, 40, 41). However, EMT features such as elongation, less adhesion and proliferation, and more migration were present in DR cells but not in CZR. Differential expression analysis in CZR versus DR models also revealed a more marked mesenchymal phenotype in DR cells and a more notable epithelial phenotype in CZR cells. These results also suggest that EMT is not a binary state but a transitional process in which cells can undergo a stable intermediate EMT phenotype (42).

It has been described in mice PC models that CZ may revert EMT by redifferentiation of mesenchymal cells into an epithelial phenotype (43). Accordingly, in this work we found that CZ restored *ESRP1* expression in DU-145DR cells and decreased *AXL* levels in DR models. This may suggest a potential explanation of the distinct clinical benefit observed in our series between D and CZ according to EMT phenotype.

There has been recent emerging evidence in the literature about the capacity of androgen suppression and ARSI to induce a NE phenotype (2). However, whether taxanes could also be partly responsible of a NE growing pattern needs to be further explored. Increased levels of the NE markers *SYP*, *MYCN*, and *CHGA* in dose-response experiments with taxanes are a novel observation of this study, reinforcing the hypothesis that chemotherapy could also contribute to the acquisition of a NE phenotype.

*ESRP1* expression has been recently considered an independent prognostic biomarker in early PC (44). *In silico* analyses suggest that *ESRP1* could be an important driver in tumor progression and NE differentiation, since its amplification frequency increase from primary tumors to NEPC. In our series we found that *ESRP1* expression in tumor positively correlated with NE markers, such as *AURKA* and *EZH2*, and negatively with the EMT markers *ZEB1* and *AXL*. ESRP1 mediates alternative splicing of a panel of transcripts involved in maintaining epithelial features, becoming then an important EMT regulator (45). During EMT, direct repression of ESRP1 expression through ZEB1 occurs (46), with subsequent changes in protein isoforms that regulate actin cytoskeleton, cell adhesion, migration and cell polarity, providing cells with mesenchymal characteristics (45). ESRP1 could be also important in regulating other cell plasticity phenotypes, since overlapping between signaling pathways involved in these mechanisms has been described (2).

We also observed changes in EMT and NE markers in mRNA expression after taxanes treatment in CTCs from mCRPC patients. It has been described that the majority of CTCs exhibits an intermediate EMT phenotype with both epithelial and mesenchymal markers (42, 47), thus we considered to use a customized mixture of antibodies (PSCA, EPCAM, and CDH2) in order to capture prostatic CTCs with epithelial and/or mesenchymal characteristics. The high intra and inter-patient heterogeneity observed may be probably translating the complexity to study in patients the cell plasticity process. Of note, *ZEB1* post-treatment expression was higher in those patients with PSA-progression at the time of CTCs analysis, supporting the role of EMT in taxane resistance in patients. *CDH1* expression was also significantly higher in post-treatment samples. The fact that CDH1 may have a role in CTCs clusters formation (48, 49) contributing to cell migration and spread, could account for this result.

Results from this study also suggest that primary tumor phenotype can affect taxane benefit during CRPC, which may be different between D and CZ. Here we defined an EMT-expression pattern characterized by *ESRP1* down-regulation and *ZEB1* and *AXL* overexpression, which correlated with a better PSA-PFS to CZ and poorer to D. This observation reinforces the existence of a different mechanism of action for each taxane, depending on the EMT profile, and the potential role of CZ in the reversion of the EMT phenotype (43). We also found that high *ESRP1* expression independently predicted better PSA-PFS to D, but worse to CZ. Moreover, a significant interaction between the type of taxane treatment and *ESRP1* levels regarding PSA-PFS was observed. In relation with that, RNA-seq data from Abida et al. (35) supported the *in vitro* findings regarding the diminishing levels of *ESRP1* mRNA after taxanes-exposure. These results agree with the acquisition of EMT phenotypic changes by taxanes treatment. Overall, these data suggest that *ESRP1* may be a potential biomarker of taxane sensitivity, although a prospective validation is required.

In our series, EMT was not associated with a lower OS. While the role of EMT in resistance to therapy and progression of the disease is well supported by the literature (50), there are, however, some discrepancies regarding its association with OS (51, 52). These controversies may arise from the heterogeneity of the tumors, the spectrum of EMT phenotypes induced by different treatments and their different implication in metastasis (47), as well as the different methodologies used.

According to NE dedifferentiation, we show that the expression of NE markers in primary tumor is associated with adverse outcome in patients treated with D or CZ. Recently, a randomized phase II trial of CZ vs CZ-Carboplatin in mCRPC patients have shown more activity, PSA-PFS and OS of the combination (53). Thus, the early introduction of carboplatin in patients with NE expression in primary tumors may be explored as a strategy to improve clinical outcome of these patients.

In conclusion, this study supports the role of tumor cell plasticity in clinical outcome of mCRPC patients as well as in primary (when present in primary non-castrate tumors) and acquired resistance to taxanes. The EMT profile expression in primary tumor is differentially associated with D or CZ benefit and NE dedifferentiation is associated with adverse outcome to D or CZ. Further research of this complex process in longer series of patients is needed to validate these results.

## Data Availability Statement

The datasets presented in this study can be found in online repositories. The names of the repository/repositories and accession number(s) can be found below: https://www.ncbi.nlm.nih.gov/geo/, GSE158494.

## Ethics Statement

The studies involving human participants were reviewed and approved by Ethics Committees of all participating centers: Hospital Clínic, Hospital Germans Trias i Pujol, Hospital del Mar, Vall d’Hebron University Hospital, Fundació Althaia Manresa, and Hospital Universitari Doctor Josep Trueta. The patients/participants provided their written informed consent to participate in this study.

## Author Contributions

Study conception and design: NJ, ÒR, MM-A, and BM. Development of methodology: NJ, ÒR, RM, MM-G, LN-D, GC, JL, PF, LR-C, SD, and MM-A. Acquisition of data (acquired and managed patients): ÒR, IV, AF, AR-V, JC, CS, MD, NS-G, AP, and BM. Analysis and interpretation of data: NJ, ÒR, RM, MM-G, LN-D, GC, JL, AP, MM-A, and BM. Manuscript preparation and editing: NJ, ÒR, RM, MM-A, and BM. All authors contributed to the article and approved the submitted version.

## Funding

This work was supported by grants from Instituto de Salud Carlos III-Subdirección General de Evaluación y Fomento de la Investigación [PI12/01226, PI15/676 and PI18/714] and co-funded by the European Regional Development Fund (ERDF). Institutional funding from CERCA Programme/Generalitat de Catalunya is gratefully acknowledged. This work was developed at the Centro Esther Koplowitz, Barcelona, Spain.

## Conflict of Interest

Authors have provided the following relationships with companies (which may not be related to the subject matter of this manuscript): NJ: travel and accommodation from Sanofi. ÒR: speaker’s bureau from BMS, Ipsen and Pfizer; travel expenses from Ipsen and Pfizer. AF: consulting or advisory role by Roche, Sanofi and Janssen; research funding by Astra-Zeneca, Astellas and Pierre Fabre; travel accommodation expenses from Astra-Zeneca, Roche and Astellas. AR-V: research support from MSD, Pfizer and Takeda; speakers’ bureau honoraria from Astellas, Astra-Zeneca, Bayer, Bristol-Myers Squibb, Janssen, MSD, Pfizer, Roche, Ipsen and Sanofi-Aventis; consultant/advisory board member for Astellas, Bayer, Bristol-Myers Squibb, Janssen, MSD, Pfizer, Ipsen, Clovis and Roche. JC: advisory board from Bayer, Johnson&Johnson, Bristol-Myers Squibb, Atellas Pharma, Pfizer, Sanofi, MSD, Roche, Astra-Zeneca; speakers’ bureau from Bayer, Johnson&Johnson, Asofarma and Astellas. CS: consulting or advisory role from Bristol-Myers Squibb, Ipsen, Sanofi, Pfizer, EUSA Pharma, Astellas, Novartis; speakers’ bureau from Bristol-Myers Squibb, Ipsen, Pfizer, Roche/Genentech, Astra-Zeneca; travel expenses by Bristol-Myers Squibb and Roche. MD: advisory role and speaker honoraria by Roche, Bristol-Myers Squibb, MSD, Sanofi, Pfizer; travel accommodation expenses from Roche, Astellas and Janssen. NS-G: advisory board and accommodation support from Pfizer, Astellas, Bristol-Myers Squibb and Ipsen. AP: lecture fees from Roche, Pfizer, Novartis, Amgen, BMS, Daiichi Sankyo and Nanostring technologies; advisory role/consultancy from Pfizer, Novartis, Amgen, Puma, Oncolytics Biotech, MSD and Lilly; Board Member of Breast International Group, Solti’s Foundation and Actitud frente al cáncer Foundation; research funding from Boehringer, Roche, Pfizer, Nanostring technologies, Novartis, Sysmex Europe GmbH, Medica Scientia Innovation Research, Celgene and Astellas Pharma; financial support for clinical trials from Boehringer, Lilly, Roche, Pfizer, Novartis, Amgen and Daiichi Sankyo. MM-A: travel accommodation from Bristol-Myers Squibb. BM: advisory role from Roche, Sanofi, Janssen, Astellas, Pfizer, Novartis, Bristol-Myers Squibb and Ipsen, research funding from Roche, Bayer and Janssen, and accommodation expenses from Pfizer and Janssen.

The remaining authors declare that the research was conducted in the absence of any commercial or financial relationships that could be construed as a potential conflict of interest.
