# Supplementary material for: Cell Plasticity-Related Phenotypes and Taxanes Resistance in Castration-Resistant Prostate Cancer
Source: Front Oncol. 2020 Nov 2;10:594023. doi: 10.3389/fonc.2020.594023 (PMC7667288; doi:10.3389/fonc.2020.594023)
Supplement: Supplementary file 1 [file DataSheet_1.docx]

Supplementary Figures

**page**

**Figure S1 2**

**Figure S2 3**

**Figure S3 4**

**Figure S4 5**

**Figure S5 6**

**Figure S6 7**

**Figure S7 8**

**Figure S8 9**

**
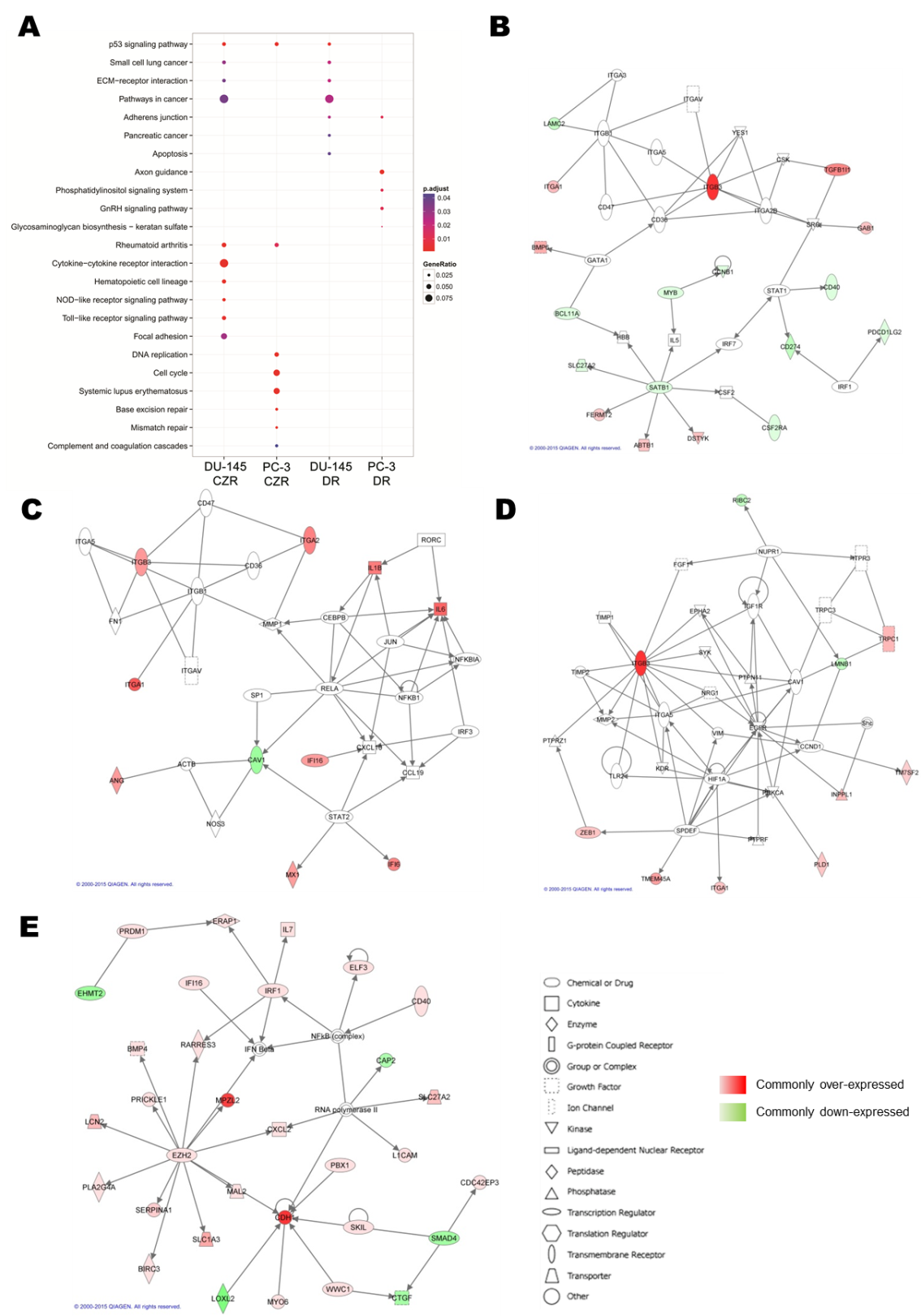
**

**Figure S1.**  KEGG Pathway Enrichment Analysis and gene networks deregulated in resistant cells from microarray data. **(A)** KEGG analysis for differently expressed genes in docetaxel-resistant (DR) and cabazitaxel-resistant (CZR) cell lines versus their respective parental cells. **(B)** The most significant (*P*<0.05) gene network deregulated in both DR cell lines versus parental cell lines (scored 15 by IPA software). **(C)** The most significant (*P*<0.05) gene network deregulated in both CZR cell lines versus parental cell lines (scored 11 by IPA software). **(D)** The most significant (*P*<0.05) gene network deregulated in both DR and CZR cell lines versus parental cell lines (scored 21 by IPA software). **(E)** The most significant (*P*<0.05) gene network deregulated in CZR versus DR cell lines (scored 45 by IPA software).

**
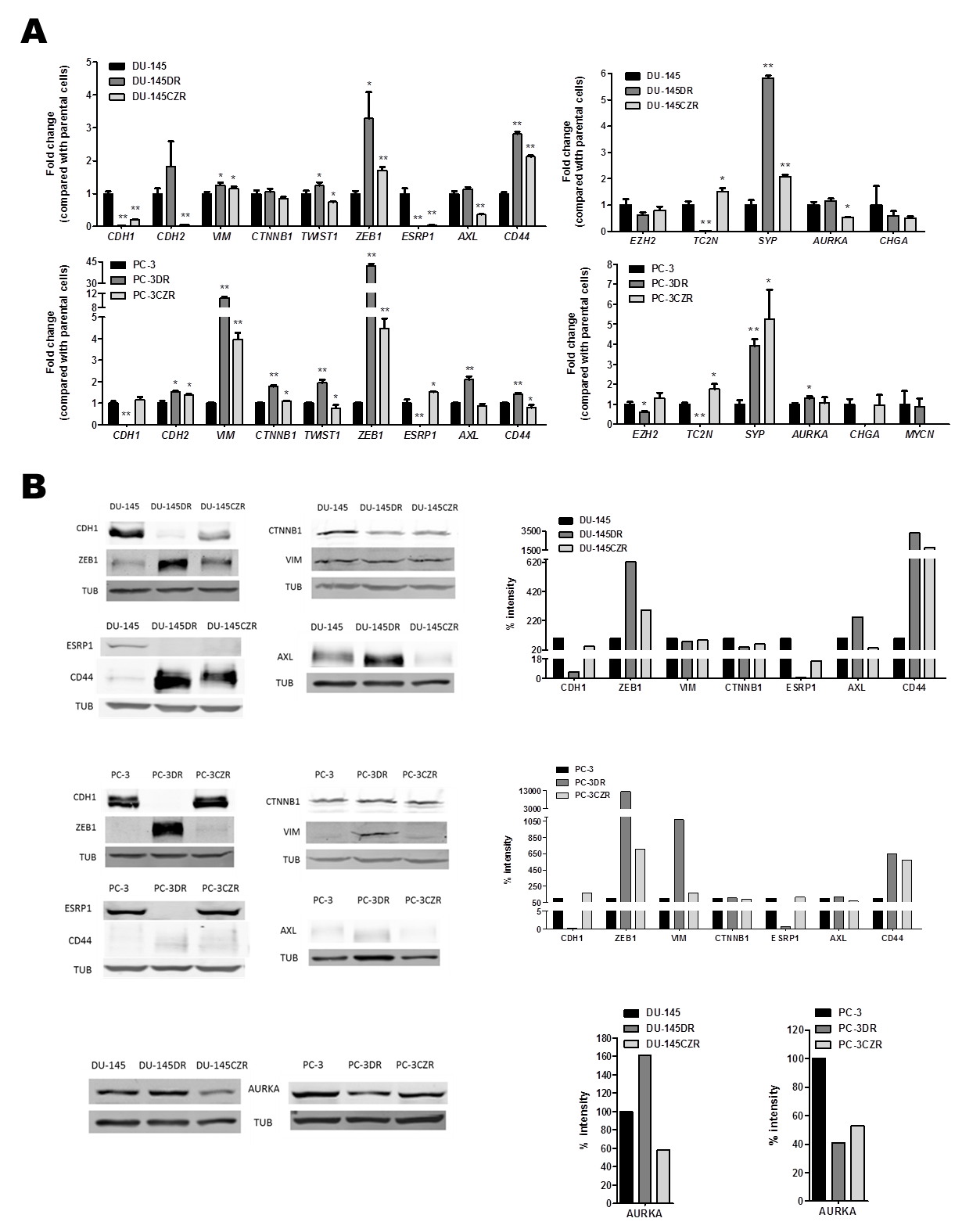
Figure S2.** Gene expression of EMT, SCL and NE markers in resistant cells. **(A)** Gene expression of EMT, SCL (CD44) and NE markers by qRT-PCR in parental and resistant cell lines. Data shown are the mean ± SD of cell lines from triplicate experiments. Significant differences respect to parental cell line are indicated as **P*<0.05, ***P*<0.001 (Student t-test). **(B)** Western Blot analysis and quantification in parental and resistant cell lines of EMT, SCL (CD44) and NE (AURKA) markers. Tubulin (TUB) was used as load control.


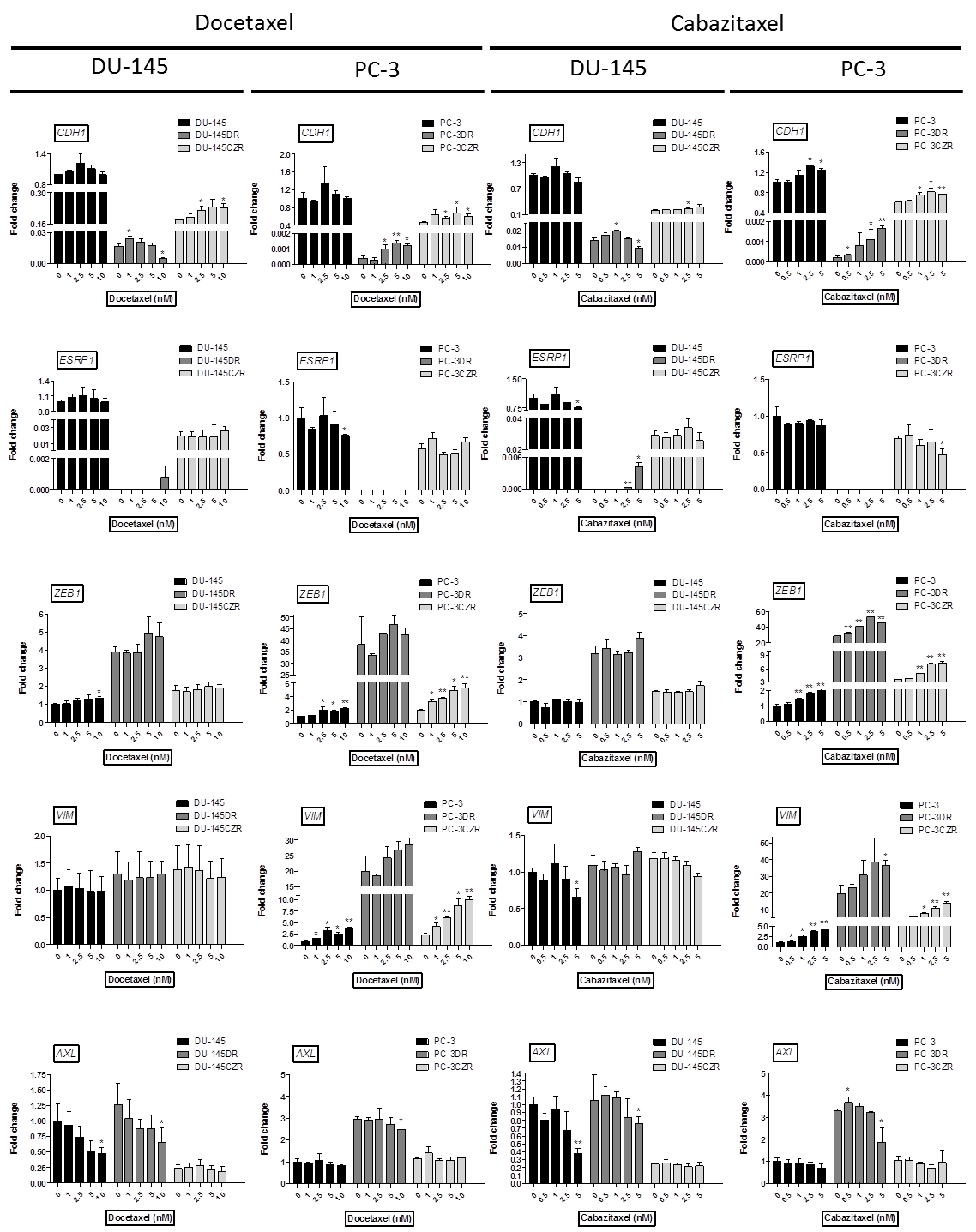


**Figure S3.** Effect of docetaxel (D) and cabazitaxel (CZ) exposure on EMT gene expression markers in cell lines. Dose-response experiments analysed by qRT-PCR of EMT markers. Cells were exposed to D and CZ for 48 h. Mean ± SD of fold change data by triplicate is shown. Parental cells at 0 nM were considered the reference in each graph. Significant differences respect to 0 nM in each cell line are indicated as **P*<0.05, ***P*<0.001 (Student t-test). DR: docetaxel-resistant; CZR: cabazitaxel-resistant.


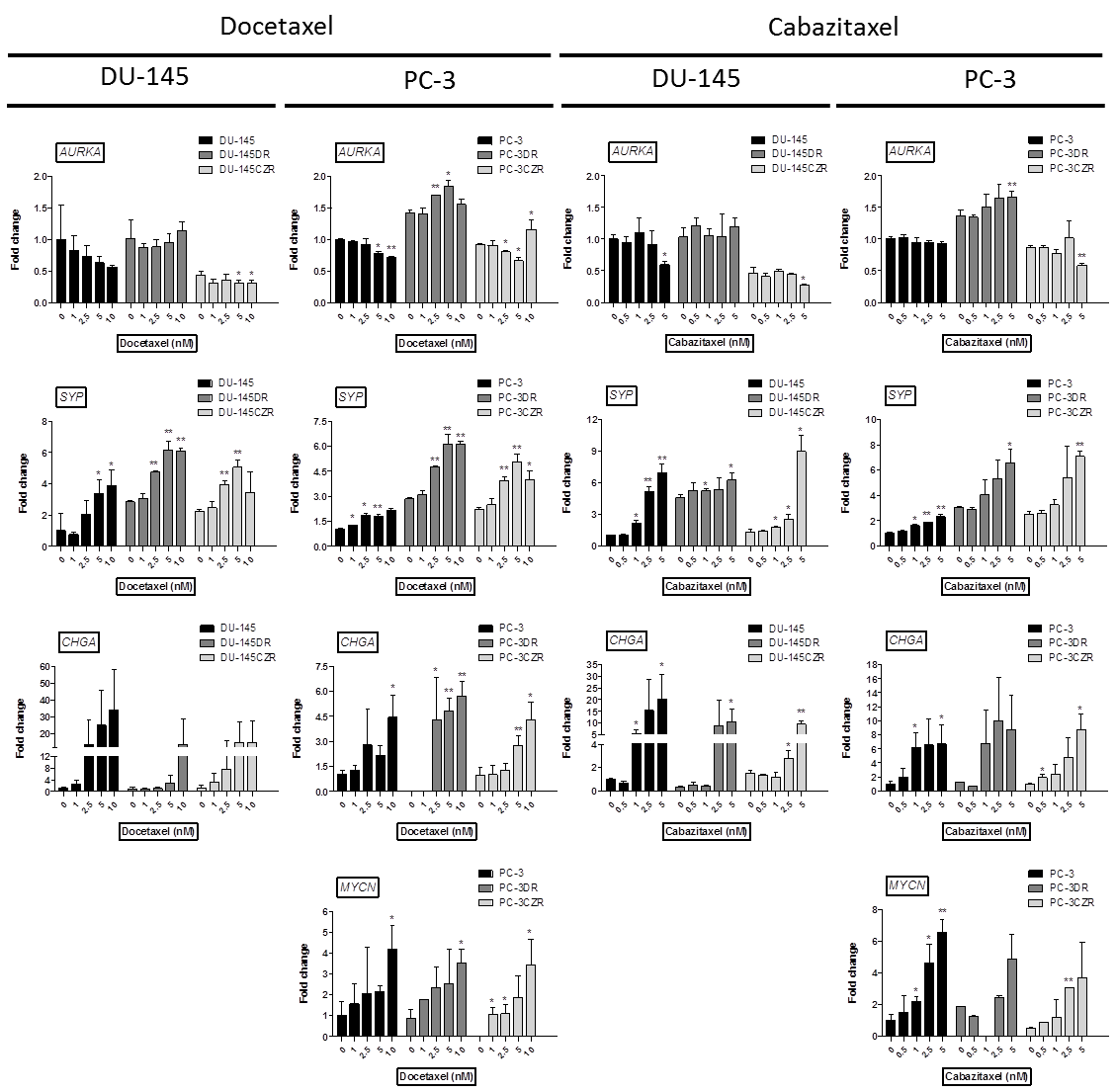


**Figure S4**. Effect of docetaxel (D) and cabazitaxel (CZ) exposure on NE gene expression markers in cell lines. Dose-response experiments analysed by qRT-PCR of NE markers. Cells were exposed to D and CZ for 48 h. Mean ± SD of fold change data by triplicate is shown. Parental cells at 0 nM were considered the reference in each graph. Significant differences respect to 0 nM in each cell line are indicated as **P*<0.05, ***P*<0.001 (Student t-test). DR: docetaxel-resistant; CZR: cabazitaxel-resistant.

**
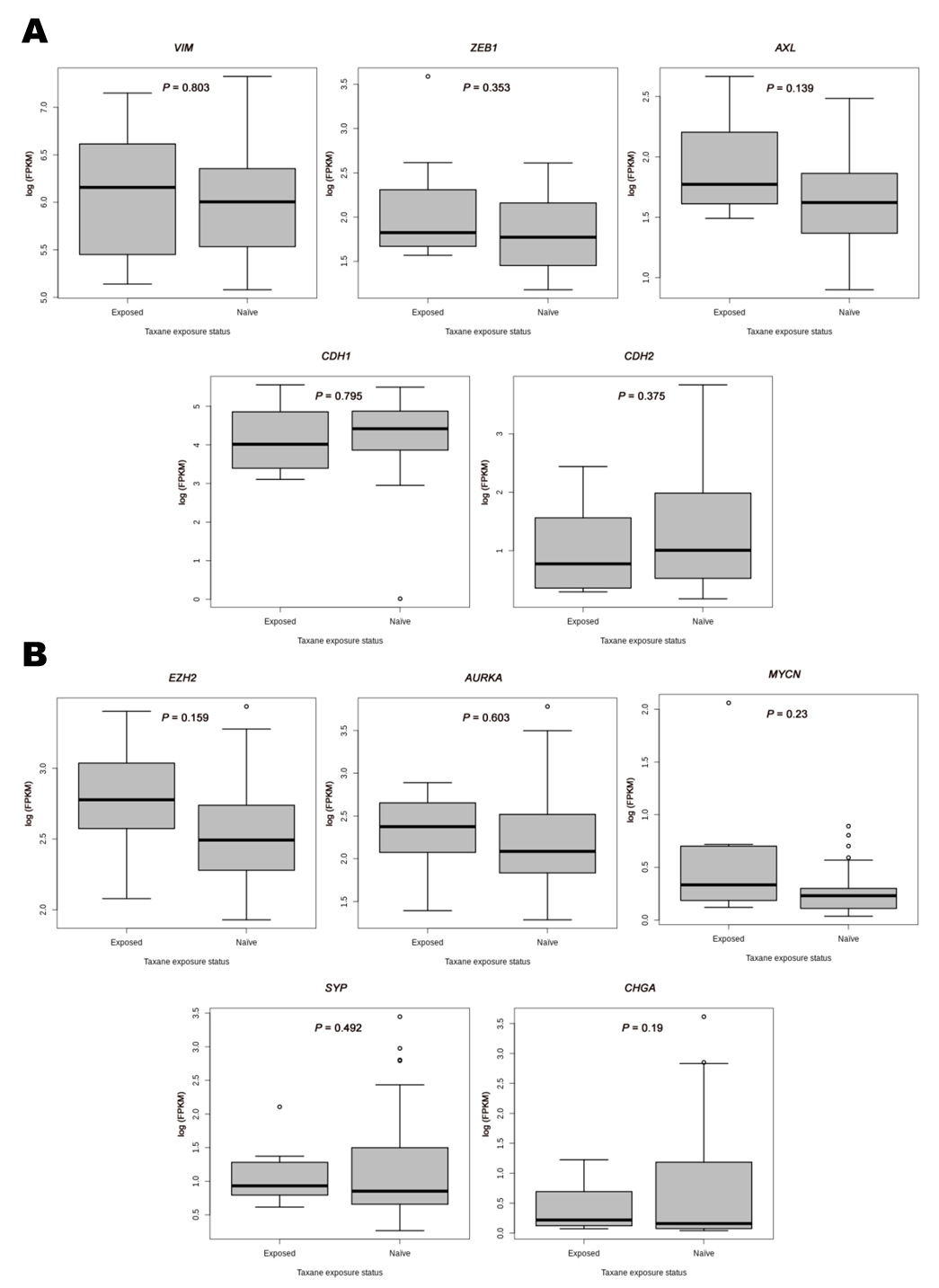
**

**Figure S5.** *In silico* analysis of EMT and NE markers according to taxane exposure of tumor samples. Boxplots of the log FPKM (Fragments Per Kilobase of exon per Million fragments mapped) from RNA-seq data representing mRNA expression of EMT **(A)** and NE **(B)** markers in taxane-naïve vs taxane-exposed mCRPC tumor samples from ARSI-naïve cohort of Abida et al. (1). Welch's *t*-test was used for means comparison.

1. Abida W, Cyrta J, Heller G, Prandi D, Armenia J, Coleman I, Cieslik M, Benelli M, Robinson D, Allen EMV, et al. Genomic correlates of clinical outcome in advanced prostate cancer. *PNAS* (2019) **116**:11428–11436. doi:10.1073/pnas.1902651116

**
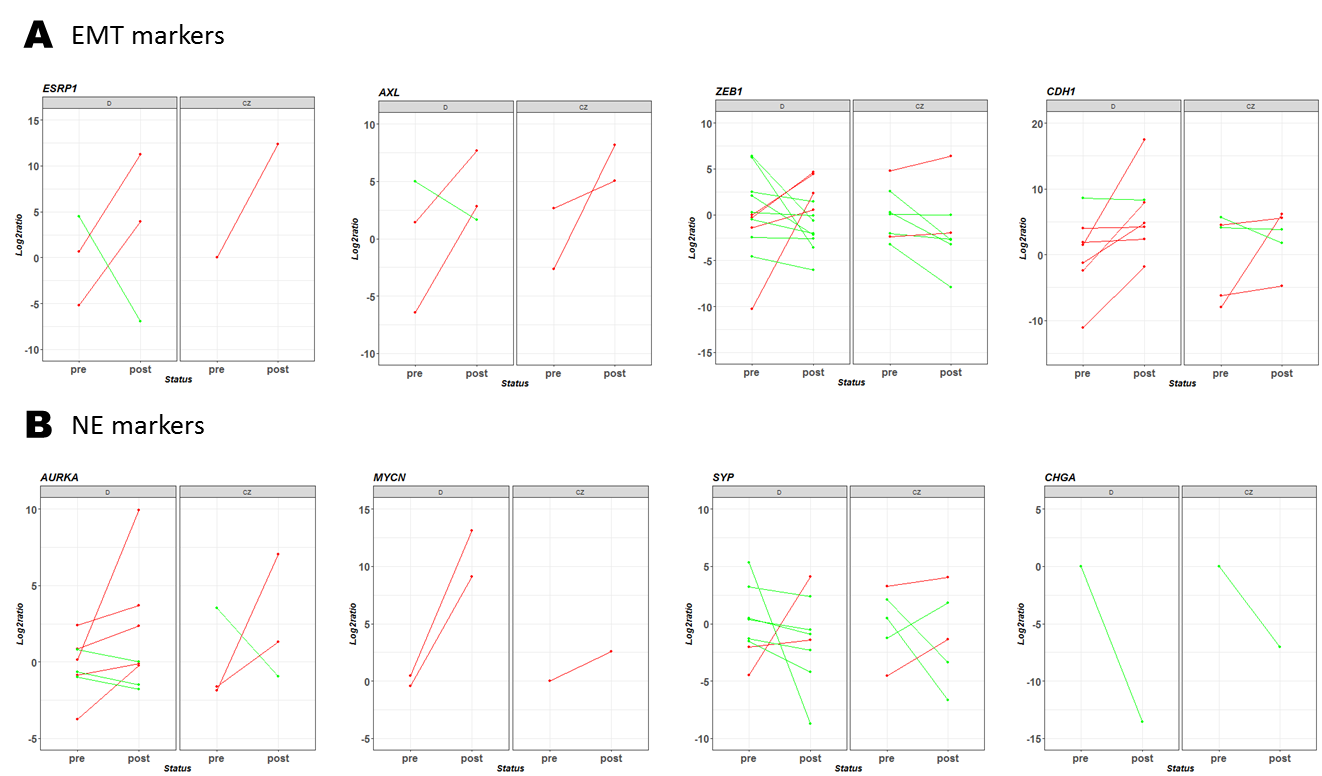
**

**Figure S6.** Changes in gene expression markers in CTCs after docetaxel (D) and cabazitaxel (CZ)-treatment by qRT-PCR. EMT **(A)** and NE-related **(B)** markers expression levels (Log2ratio), pre and post-taxanes treatment in CTCs. Red lines: overexpression; Green lines: underexpression.


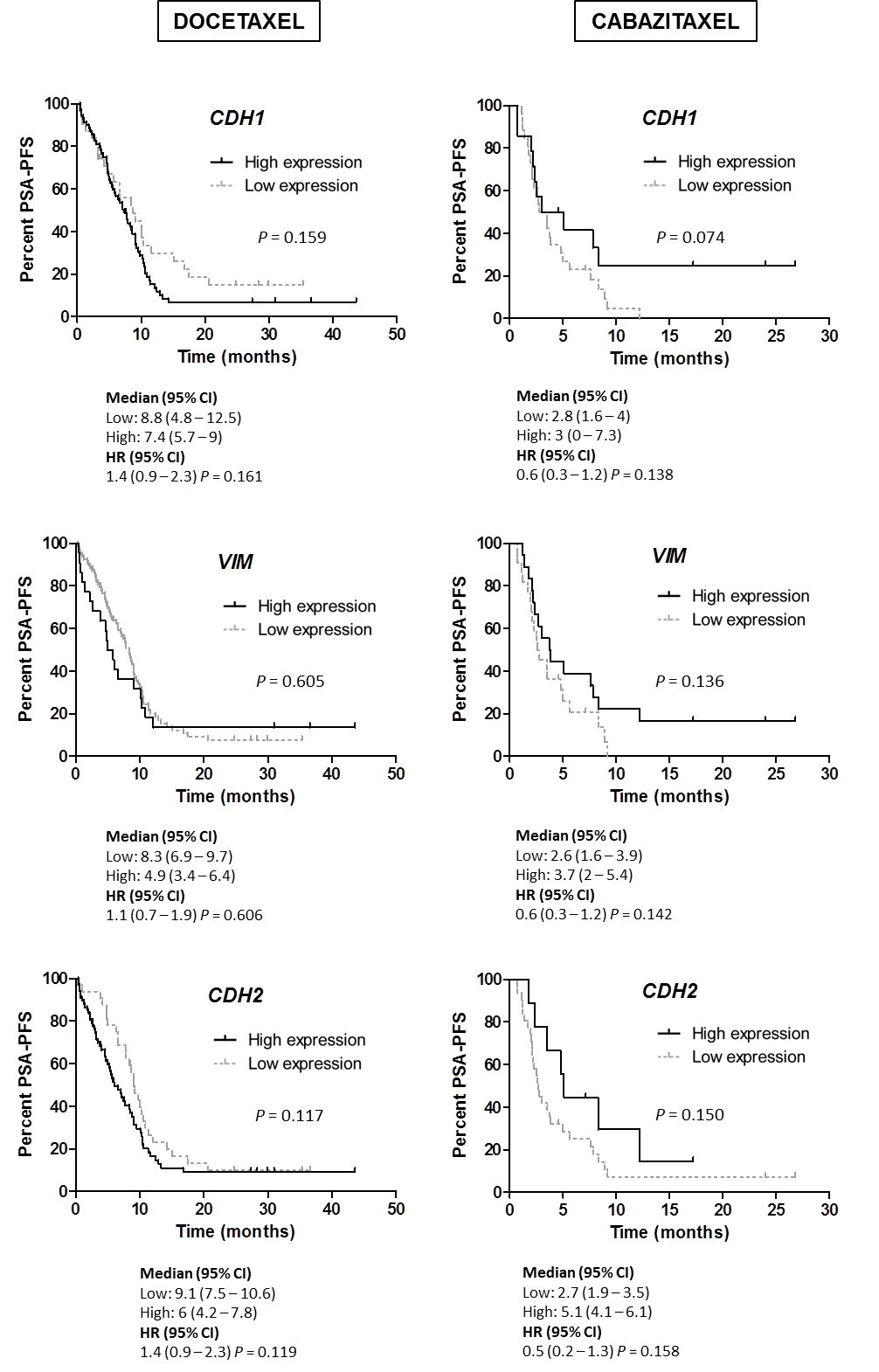


**Figure S7.** PSA progression-free survival (PSA-PFS) analysis in taxanes-treated patients according to gene expression of EMT markers in tumor samples by qRT-PCR. CI: confidence interval; HR: hazard ratio.


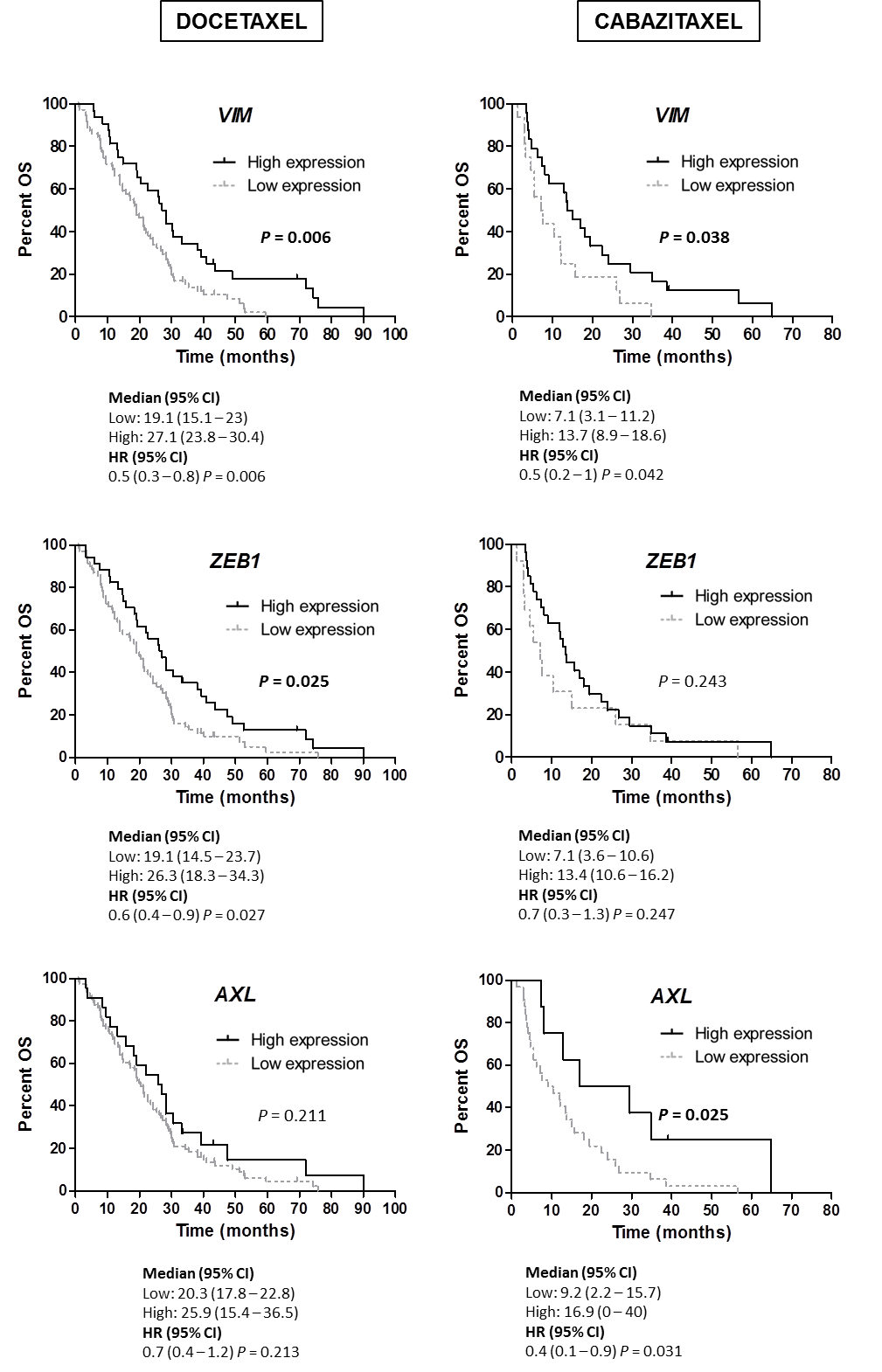


**Figure S8.** Overall survival (OS) analysis in taxanes-treated patients according to gene expression of EMT markers in tumor samples by qRT-PCR. CI: confidence interval; HR: hazard ratio.
