# Supplementary material for: Cell Plasticity-Related Phenotypes and Taxanes Resistance in Castration-Resistant Prostate Cancer
Source: Front Oncol. 2020 Nov 2;10:594023. doi: 10.3389/fonc.2020.594023 (PMC7667288; doi:10.3389/fonc.2020.594023)
Supplement: Supplementary file 2 [file DataSheet_2.docx]

Supplementary Tables

**pages**

**Table S1 2-19**

**Table S2 20-24**

**Table S3 25**

**Table S4 26-41**

**Table S5 42**

**Table S6 43**

**Table S7 44**

**Table S8 45**

**Table S1.** Commonly differentially expressed genes in docetaxel-resistant (DR) versus parental cells from microarray data. Fold change expression of 625 commonly differentially expressed genes in DR vs parental cell lines in both cell models (DU-145 and PC-3). FC: Fold Change; FDR: False Discovery Rate.

| **COMMON UP-REGULATED GENES in DR vs. PARENTALS** | | | | | |
| --- | --- | --- | --- | --- | --- |
| **DU-145DR vs. DU-145** | | | **PC-3DR vs. PC-3** | | |
| **Gene Symbol** | **FC** | **FDR** | **Gene Symbol** | **FC** | **FDR** |
| *SMAD4* | 10.04267 | 1.66E-07 | *CYBRD1* | 8.582694 | 4.68E-07 |
| *NID2* | 8.230832 | 9.75E-06 | *ZEB1* | 8.471464 | 3.11E-07 |
| *ITGB3* | 6.734176 | 5.5E-07 | *CD70* | 8.284467 | 4.87E-07 |
| *CPA4* | 5.862038 | 9.34E-06 | *ITGA1* | 8.102511 | 2.76E-07 |
| *EFR3B* | 4.426113 | 4.03E-05 | *B4GALT6* | 7.440817 | 6.43E-08 |
| *PTPRM* | 4.2902 | 2.18E-06 | *ITGB3* | 6.891471 | 4.49E-07 |
| *IGFBP3* | 4.189652 | 4.81E-06 | *FGFR1* | 6.656544 | 2.55E-07 |
| *NDRG1* | 4.183178 | 5.08E-06 | *PRKD1* | 6.101487 | 7.64E-07 |
| *TP53INP1* | 4.072966 | 3.39E-06 | *NOG* | 5.952859 | 1.35E-07 |
| *GPR135* | 3.901578 | 5.95E-06 | *SLC46A3* | 4.870741 | 2.11E-07 |
| *CCDC80* | 3.871559 | 3.81E-06 | *EFR3B* | 4.710836 | 2.18E-07 |
| *EML1* | 3.859493 | 4.72E-05 | *LTBP1* | 4.645106 | 1.54E-07 |
| *MLLT11* | 3.707797 | 6.77E-06 | *FERMT2* | 4.341577 | 4.05E-07 |
| *PIK3C3* | 3.551496 | 3.73E-06 | *TUBB4A* | 4.117608 | 8.54E-06 |
| *SLCO4A1* | 3.518105 | 1.2E-05 | *ZFYVE1* | 4.060574 | 4.36E-07 |
| *TGFB1I1* | 3.491955 | 0.000183 | *TTC28* | 4.036168 | 3.48E-06 |
| *PLAG1* | 3.47603 | 0.000757 | *IFI6* | 3.994021 | 3.69E-06 |
| *ID2-AS1* | 3.412822 | 0.000626 | *GAB1* | 3.850918 | 9.47E-07 |
| *ST6GALNAC2* | 3.302166 | 2.95E-05 | *HTRA1* | 3.834652 | 3.92E-07 |
| *ARHGAP24* | 3.230861 | 3.59E-05 | *SCARNA17* | 3.759435 | 8.07E-07 |
| *CKB* | 3.193146 | 7.39E-05 | *PLSCR4* | 3.741108 | 1.53E-06 |
| *LOC101927144* | 3.140404 | 5.76E-06 | *ARHGAP23* | 3.729515 | 3.59E-05 |
| *TMEM45A* | 3.137568 | 1.28E-05 | *C5orf42* | 3.70875 | 1.42E-06 |
| *ATP2B4* | 3.097593 | 9.78E-06 | *ST6GALNAC2* | 3.672399 | 9.87E-07 |
| *ACAA2* | 3.092403 | 1.25E-05 | *MXRA7* | 3.52248 | 7.64E-07 |
| *TENM3* | 2.958109 | 2.75E-05 | *EHD2* | 3.33273 | 0.000163 |
| *PROS1* | 2.897572 | 2.64E-05 | *PLD1* | 3.294461 | 8.69E-07 |
| *CNTNAP1* | 2.851755 | 3.95E-05 | *TGFB1I1* | 3.277581 | 2.03E-05 |
| *HTRA1* | 2.806868 | 0.000392 | *SFTA1P* | 3.264354 | 4.43E-05 |
| *CYBRD1* | 2.778985 | 4.92E-05 | *SYDE1* | 3.257876 | 1.48E-06 |
| *AOX1* | 2.760111 | 2.46E-05 | *PSEN1* | 3.250182 | 1.14E-06 |
| *SMAD2* | 2.721174 | 2.33E-05 | *CLU* | 3.202143 | 1.16E-06 |
| *SLC46A3* | 2.65786 | 3.95E-05 | *ZNF844* | 3.10327 | 2.78E-05 |
| *PCAT6* | 2.656328 | 0.00017 | *ANKRD20A5P* | 3.053486 | 3.45E-06 |
| *SCARNA17* | 2.648484 | 3.74E-05 | *ABHD3* | 3.051972 | 3.56E-05 |
| *RBM24* | 2.639551 | 0.000152 | *AOX1* | 3.044888 | 1.45E-06 |
| *CRIP1* | 2.592878 | 0.001077 | *GSPT2* | 3.040904 | 3.21E-05 |
| *TFAP2C* | 2.585471 | 0.000317 | *CPA4* | 3.020461 | 9.85E-06 |
| *ABHD3* | 2.579634 | 2.95E-05 | *SYT11* | 2.98509 | 1.24E-05 |
| *LEPRE1* | 2.575279 | 0.000129 | *ZNF382* | 2.937677 | 0.001009 |
| *TMCC1-AS1* | 2.560769 | 2.69E-05 | *RHOBTB3* | 2.8977 | 2.4E-06 |
| *HIST1H2BC* | 2.547629 | 0.000883 | *KDELC1* | 2.876031 | 4.83E-05 |
| *MXRA7* | 2.544641 | 2.69E-05 | *MMP16* | 2.8718 | 4.05E-06 |
| *SEC14L1* | 2.541646 | 0.000185 | *PTPRM* | 2.851846 | 2.04E-06 |
| *EPG5* | 2.535484 | 3.18E-05 | *LEPRE1* | 2.838032 | 2.49E-05 |
| *ATP9B* | 2.509639 | 0.000252 | *KLHL24* | 2.837144 | 0.000154 |
| *TTC28* | 2.482349 | 0.000352 | *ABCA1* | 2.816748 | 2.37E-06 |
| *ANKRD20A5P* | 2.444589 | 0.003679 | *NID2* | 2.807037 | 7.52E-05 |
| *ANXA2R* | 2.439025 | 6.89E-05 | *NTN4* | 2.793788 | 6.67E-05 |
| *WDR7* | 2.438288 | 7.15E-05 | *CNTNAP1* | 2.762463 | 9.7E-06 |
| *PLAT* | 2.433867 | 0.000185 | *LOC100506844* | 2.732017 | 1.23E-05 |
| *POLI* | 2.417575 | 0.000369 | *DOCK10* | 2.6963 | 1.52E-05 |
| *FGFR1* | 2.415738 | 0.000122 | *SEC24D* | 2.693262 | 2.75E-06 |
| *CITED2* | 2.410884 | 0.000129 | *TXNDC16* | 2.679082 | 1.31E-05 |
| *PLEKHG4* | 2.40772 | 0.000227 | *IGFBP3* | 2.65869 | 4.66E-05 |
| *ZSWIM5* | 2.404597 | 5.77E-05 | *CLYBL* | 2.653785 | 9.12E-06 |
| *LOC100506100* | 2.40229 | 3.78E-05 | *PIAS2* | 2.651326 | 2.18E-06 |
| *TMX4* | 2.400775 | 0.000911 | *LOC101929643* | 2.631016 | 0.001316 |
| *ARHGEF6* | 2.392775 | 7.83E-05 | *ANTXR2* | 2.626603 | 8.36E-06 |
| *RAB27B* | 2.378781 | 0.000373 | *BMP6* | 2.571241 | 0.000123 |
| *PTPRG* | 2.362689 | 8.68E-05 | *GPR137C* | 2.559625 | 1.17E-05 |
| *PINK1* | 2.360078 | 0.000426 | *LOC101927144* | 2.556115 | 0.003218 |
| *NTN4* | 2.347121 | 0.000167 | *FAM210B* | 2.553737 | 3.74E-06 |
| *DLGAP1-AS1* | 2.344586 | 8.88E-05 | *CRIP1* | 2.548655 | 3.76E-05 |
| *ARHGAP23* | 2.338658 | 0.000294 | *C5* | 2.543353 | 5.93E-06 |
| *TRPC1* | 2.333377 | 0.000426 | *ADAM23* | 2.423116 | 9.63E-05 |
| *PTPRS* | 2.320663 | 6.77E-05 | *ARHGAP24* | 2.417116 | 1.31E-05 |
| *BMP6* | 2.317541 | 7.67E-05 | *TMCC1-AS1* | 2.399797 | 8.91E-06 |
| *GPR137C* | 2.307134 | 0.000117 | *ATP2B4* | 2.398291 | 9.56E-06 |
| *LOC101929500* | 2.285052 | 0.015235 | *TENM3* | 2.38799 | 7.28E-06 |
| *C5orf42* | 2.281344 | 0.001272 | *RNF125* | 2.372227 | 9.11E-05 |
| *ANTXR2* | 2.273965 | 9.13E-05 | *LRP1* | 2.355126 | 1.25E-05 |
| *INPPL1* | 2.271854 | 0.000263 | *GALNT1* | 2.342574 | 1.31E-05 |
| *CTSF* | 2.267259 | 0.000197 | *ANKRD36BP2* | 2.327232 | 3.25E-05 |
| *TTLL11-IT1* | 2.266995 | 0.000676 | *PARD6G* | 2.321915 | 1.57E-05 |
| *ARHGAP31* | 2.256116 | 0.000537 | *PDE3B* | 2.317162 | 4.74E-05 |
| *TMEM136* | 2.241981 | 0.000129 | *CKB* | 2.314155 | 1.55E-05 |
| *MMP16* | 2.240972 | 0.000333 | *ZBED6* | 2.287252 | 3.96E-05 |
| *GSPT2* | 2.239351 | 0.001513 | *MSRB3* | 2.255249 | 4.39E-05 |
| *HABP4* | 2.225804 | 0.00019 | *FUT11* | 2.22278 | 2.19E-05 |
| *ZNF185* | 2.216588 | 0.000274 | *MRC2* | 2.212216 | 1.41E-05 |
| *CXXC1* | 2.213737 | 0.00021 | *DSC3* | 2.211257 | 2.02E-05 |
| *PGAP1* | 2.207936 | 0.000853 | *ANXA2R* | 2.200243 | 0.000259 |
| *ZSCAN30* | 2.20757 | 0.000307 | *POLI* | 2.198293 | 0.000162 |
| *RECK* | 2.201805 | 0.000531 | *TPP1* | 2.186242 | 1.53E-05 |
| *MBD1* | 2.189268 | 0.000128 | *MICU3* | 2.177157 | 0.000164 |
| *FBXO43* | 2.188882 | 0.000128 | *DFNB31* | 2.169257 | 3.61E-05 |
| *KLHL24* | 2.162067 | 0.001255 | *ZFYVE26* | 2.167983 | 3.77E-05 |
| *LOC100132356* | 2.150913 | 0.000481 | *MAP2K6* | 2.144523 | 8.53E-05 |
| *LOC100288911* | 2.145037 | 0.000645 | *GPR135* | 2.127254 | 0.000244 |
| *ZNF382* | 2.141418 | 0.000592 | *MAGED2* | 2.11715 | 4.67E-05 |
| *CHN1* | 2.137521 | 0.000155 | *ACAA2* | 2.080701 | 4.99E-05 |
| *LTBP1* | 2.119183 | 0.000188 | *ATG14* | 2.072059 | 0.000108 |
| *HS1BP3* | 2.10543 | 0.000718 | *PLAT* | 2.062307 | 3.73E-05 |
| *ABCA1* | 2.103153 | 0.001082 | *LOC101559451* | 2.05292 | 0.00016 |
| *EHD2* | 2.102474 | 0.000347 | *PIK3C3* | 2.04649 | 7.26E-05 |
| *PBXIP1* | 2.089517 | 0.000595 | *ABTB1* | 2.043757 | 0.000101 |
| *TPP1* | 2.088536 | 0.002488 | *GHR* | 2.0402 | 2.23E-05 |
| *FXR1* | 2.083463 | 0.000336 | *CNPY4* | 2.036606 | 4.36E-05 |
| *LOC101559451* | 2.080157 | 0.000272 | *LOC101929500* | 2.026937 | 0.000396 |
| *SYT11* | 2.079665 | 0.000167 | *GRK5* | 2.023604 | 0.00034 |
| *IER3IP1* | 2.071442 | 0.001121 | *CCDC80* | 2.020907 | 7.19E-05 |
| *FAM114A1* | 2.068561 | 0.00013 | *ARHGAP31* | 2.007747 | 4.83E-05 |
| *PDE3B* | 2.067829 | 0.000586 | *SASH1* | 1.992352 | 5.79E-05 |
| *SYDE1* | 2.063674 | 0.000286 | *WIPI1* | 1.987061 | 3.13E-05 |
| *SGCB* | 2.061028 | 0.000247 | *TIMP2* | 1.980456 | 0.000101 |
| *RTTN* | 2.054342 | 0.000156 | *CAMK2D* | 1.977835 | 2.87E-05 |
| *SLC38A6* | 2.052637 | 0.002058 | *HSPG2* | 1.97695 | 7.59E-05 |
| *OLFML2A* | 2.052123 | 0.000341 | *DLGAP1-AS1* | 1.970475 | 0.002356 |
| *PLOD1* | 2.031768 | 0.000396 | *LOC153684* | 1.966135 | 0.000437 |
| *HMOX1* | 2.026617 | 0.001825 | *MAP3K3* | 1.964734 | 0.000133 |
| *SEC11C* | 2.013965 | 0.000551 | *TRAPPC8* | 1.962852 | 6.25E-05 |
| *WIPI1* | 2.009064 | 0.000365 | *CPEB2* | 1.960025 | 0.001487 |
| *PLCD4* | 2.005979 | 0.006039 | *MVP* | 1.953595 | 0.00014 |
| *CHKA* | 2.004484 | 0.000318 | *EPG5* | 1.949404 | 3.88E-05 |
| *ANKRD36BP2* | 2.002486 | 0.000786 | *RECK* | 1.947715 | 0.000238 |
| *DFNB31* | 1.999202 | 0.000189 | *CYB5R3* | 1.946583 | 7.1E-05 |
| *WDFY2* | 1.998043 | 0.000308 | *ARHGEF6* | 1.944857 | 0.001313 |
| *TXNDC16* | 1.996987 | 0.000583 | *PROS1* | 1.931727 | 0.000239 |
| *SORBS3* | 1.993895 | 0.001213 | *SLC38A6* | 1.921172 | 5.03E-05 |
| *CAP2* | 1.97856 | 0.000206 | *RRAS* | 1.903692 | 9.46E-05 |
| *MIB1* | 1.974265 | 0.000437 | *FBXO43* | 1.90349 | 0.000238 |
| *WDR35* | 1.959692 | 0.000595 | *SPIRE1* | 1.901119 | 0.001326 |
| *MRC2* | 1.955417 | 0.003304 | *WDR35* | 1.896724 | 0.00036 |
| *ITGA1* | 1.948904 | 0.00408 | *ID2-AS1* | 1.896716 | 0.035614 |
| *PLSCR4* | 1.945254 | 0.000899 | *TTLL11-IT1* | 1.895723 | 2.95E-05 |
| *DYM* | 1.944678 | 0.000198 | *SMAD4* | 1.89302 | 0.000131 |
| *ZEB1* | 1.937094 | 0.001183 | *EHD3* | 1.892463 | 8.62E-05 |
| *SUCLG2-AS1* | 1.936179 | 0.000455 | *BNC2* | 1.888338 | 0.001691 |
| *RHOBTB3* | 1.933927 | 0.000217 | *PCID2* | 1.883586 | 4.34E-05 |
| *MAGED1* | 1.931177 | 0.002201 | *PTPRG* | 1.881538 | 0.000163 |
| *PDLIM5* | 1.914488 | 0.000488 | *LNP1* | 1.880311 | 0.000101 |
| *CPEB2* | 1.907194 | 0.001185 | *CRIM1* | 1.876704 | 4.36E-05 |
| *ZNF844* | 1.898255 | 0.028206 | *USP12* | 1.876011 | 0.000331 |
| *CREB3L2* | 1.894058 | 0.000506 | *CHN1* | 1.868821 | 0.001526 |
| *PLD1* | 1.877562 | 0.000361 | *FAM114A1* | 1.86766 | 8.36E-05 |
| *DENND5A* | 1.863476 | 0.000406 | *SCARA3* | 1.861418 | 0.000168 |
| *GHR* | 1.863034 | 0.035706 | *PINK1* | 1.859745 | 7.74E-05 |
| *CTSB* | 1.858834 | 0.000426 | *LOC100507291* | 1.855674 | 0.002881 |
| *PIAS2* | 1.857764 | 0.000736 | *CTSB* | 1.855075 | 4.22E-05 |
| *C5* | 1.8511 | 0.018939 | *SLC39A9* | 1.850343 | 4.06E-05 |
| *ARHGEF17* | 1.836221 | 0.002135 | *PCAT6* | 1.840125 | 0.000346 |
| *DOCK10* | 1.833199 | 0.000781 | *PLAG1* | 1.839725 | 0.001462 |
| *LOC101928530* | 1.832263 | 0.021875 | *CEP19* | 1.836994 | 0.000854 |
| *FAM214B* | 1.831467 | 0.001169 | *ATP9B* | 1.83662 | 0.000261 |
| *LOC100507577* | 1.831221 | 0.008908 | *C18orf54* | 1.827841 | 0.000666 |
| *ADPRHL1* | 1.826867 | 0.001155 | *WDFY2* | 1.826679 | 7.52E-05 |
| *KDSR* | 1.815439 | 0.000528 | *FXR1* | 1.815374 | 5.76E-05 |
| *TIMP2* | 1.809975 | 0.000711 | *SCD5* | 1.814245 | 0.000125 |
| *MIR4451* | 1.805691 | 0.013805 | *GRN* | 1.813773 | 8.08E-05 |
| *FUT11* | 1.80538 | 0.000687 | *IER3IP1* | 1.808176 | 0.000196 |
| *CLU* | 1.801138 | 0.001487 | *KDSR* | 1.802933 | 5.05E-05 |
| *ZBED6* | 1.798452 | 0.001229 | *DENND5A* | 1.801669 | 0.000122 |
| *LMAN1* | 1.797591 | 0.001638 | *SNX29* | 1.800463 | 5.79E-05 |
| *FMNL2* | 1.79584 | 0.000506 | *MIR24-2* | 1.798648 | 0.002125 |
| *DSTYK* | 1.794738 | 0.001526 | *PITPNM1* | 1.792551 | 0.00051 |
| *PLEKHO1* | 1.79456 | 0.005104 | *HCFC1R1* | 1.792029 | 6.29E-05 |
| *SLC26A6* | 1.793935 | 0.011667 | *EML1* | 1.784979 | 0.000143 |
| *KLF12* | 1.791137 | 0.013274 | *ZCCHC2* | 1.781912 | 0.000352 |
| *TNFRSF10C* | 1.790499 | 0.009114 | *TRPC1* | 1.781472 | 0.002809 |
| *ZCCHC2* | 1.783789 | 0.0007 | *SLC26A6* | 1.78072 | 0.000415 |
| *GRK5* | 1.782719 | 0.001714 | *GNE* | 1.768988 | 0.000125 |
| *ZSWIM8* | 1.782317 | 0.003717 | *HMOX1* | 1.762453 | 0.000379 |
| *EHD3* | 1.781408 | 0.002182 | *CD99L2* | 1.760493 | 0.000212 |
| *CRIM1* | 1.773673 | 0.00048 | *CREB3L2* | 1.759847 | 0.0001 |
| *MVP* | 1.773479 | 0.0023 | *SNORA37* | 1.755761 | 0.003634 |
| *PITPNM1* | 1.769087 | 0.005628 | *GACAT2* | 1.747959 | 0.000811 |
| *ADAM23* | 1.768979 | 0.001994 | *PDLIM5* | 1.742138 | 0.000115 |
| *ARPC5* | 1.763807 | 0.00056 | *DYM* | 1.740865 | 0.000238 |
| *MAP3K3* | 1.758803 | 0.001285 | *SEC14L1* | 1.738193 | 0.000127 |
| *DSC3* | 1.754972 | 0.002214 | *APLP1* | 1.735146 | 8.35E-05 |
| *NOG* | 1.752944 | 0.004605 | *OLFML2A* | 1.733751 | 0.000799 |
| *SNORA37* | 1.749722 | 0.003142 | *ARPC5* | 1.729856 | 0.000148 |
| *PARD6G* | 1.74741 | 0.003321 | *SOBP* | 1.729283 | 0.000669 |
| *TUBB4A* | 1.745785 | 0.002219 | *ZC3H6* | 1.725942 | 0.00033 |
| *LRP12* | 1.73821 | 0.001123 | *PLOD1* | 1.724661 | 0.000177 |
| *LOC101928868* | 1.737891 | 0.003642 | *PLEKHO1* | 1.72434 | 0.002688 |
| *GRN* | 1.732823 | 0.004237 | *DSTYK* | 1.717376 | 0.000102 |
| *ZFYVE1* | 1.727779 | 0.002949 | *PGAP1* | 1.713601 | 0.001149 |
| *TMCO3* | 1.726588 | 0.001398 | *INPPL1* | 1.70407 | 0.000254 |
| *DDHD2* | 1.725808 | 0.000901 | *PC* | 1.701944 | 0.000199 |
| *DFNA5* | 1.725342 | 0.010268 | *RAB27B* | 1.700753 | 0.000344 |
| *LOC100507291* | 1.724524 | 0.006945 | *HIST1H2BC* | 1.699044 | 0.004332 |
| *PRKD1* | 1.72208 | 0.002732 | *NDRG1* | 1.698946 | 0.000116 |
| *GACAT2* | 1.721111 | 0.005462 | *DFNA5* | 1.695648 | 0.002241 |
| *RRAS* | 1.721087 | 0.005779 | *MIB1* | 1.694641 | 0.000133 |
| *MAGED2* | 1.721055 | 0.002382 | *FMNL2* | 1.694156 | 0.000365 |
| *LOC153684* | 1.717114 | 0.00095 | *ZSWIM5* | 1.693748 | 0.000598 |
| *APLP1* | 1.714662 | 0.002819 | *CAP2* | 1.689756 | 0.000173 |
| *ABTB1* | 1.713584 | 0.000781 | *PRKCA* | 1.686657 | 0.000182 |
| *KDELC1* | 1.711741 | 0.005948 | *AGO4* | 1.682345 | 0.000611 |
| *MSRB3* | 1.710472 | 0.001345 | *TMEM136* | 1.681326 | 0.001099 |
| *GNE* | 1.709245 | 0.001203 | *WDR7* | 1.679465 | 0.000204 |
| *JMJD1C-AS1* | 1.706906 | 0.001883 | *CDR2L* | 1.679022 | 0.000294 |
| *GAB1* | 1.698258 | 0.002098 | *LOC101927365* | 1.678224 | 0.00408 |
| *CNPY4* | 1.697333 | 0.004629 | *ATP8B3* | 1.674348 | 0.001818 |
| *USP12* | 1.69502 | 0.004625 | *LRP12* | 1.673908 | 0.001102 |
| *BNC2* | 1.69268 | 0.006585 | *ARHGEF17* | 1.668951 | 0.003603 |
| *HDHD2* | 1.691957 | 0.003478 | *TMEM45A* | 1.668914 | 0.001036 |
| *CD109* | 1.688829 | 0.002083 | *DDHD2* | 1.665932 | 0.000302 |
| *SASH1* | 1.68544 | 0.001828 | *TMCO3* | 1.664349 | 0.00017 |
| *ZNF236* | 1.678345 | 0.001311 | *SLCO4A1* | 1.664201 | 0.000395 |
| *EDIL3* | 1.678072 | 0.001328 | *MIR4451* | 1.659694 | 0.020388 |
| *PCID2* | 1.677357 | 0.003385 | *SUCLG2-AS1* | 1.6567 | 0.003914 |
| *LOC100996660* | 1.676463 | 0.004756 | *PLCD4* | 1.65629 | 0.001103 |
| *TRAK1* | 1.676195 | 0.001535 | *ST6GALNAC6* | 1.653284 | 0.000254 |
| *CEP19* | 1.676019 | 0.002491 | *WDR19* | 1.652191 | 0.000497 |
| *LOC101929643* | 1.675644 | 0.013132 | *HS1BP3* | 1.651989 | 0.000202 |
| *SNX29* | 1.667622 | 0.002872 | *LOC100288911* | 1.650958 | 0.005731 |
| *CD70* | 1.667208 | 0.004077 | *GLT8D1* | 1.648268 | 0.000152 |
| *AGO4* | 1.667117 | 0.000883 | *FNBP1* | 1.645126 | 0.000366 |
| *SELM* | 1.664803 | 0.01161 | *LOC100996660* | 1.640444 | 0.00593 |
| *SEMA4G* | 1.661992 | 0.010793 | *CD109* | 1.634935 | 0.000279 |
| *WDR19* | 1.659609 | 0.005547 | *EDIL3* | 1.634917 | 0.000622 |
| *LOC101928674* | 1.653585 | 0.010793 | *HDHD2* | 1.632698 | 0.000412 |
| *ORAI3* | 1.649257 | 0.001994 | *SGCB* | 1.631992 | 0.0003 |
| *SLC39A9* | 1.647388 | 0.001178 | *TRAK1* | 1.625406 | 0.000223 |
| *ST6GALNAC6* | 1.642439 | 0.005532 | *ZSCAN30* | 1.624537 | 0.000839 |
| *TM7SF2* | 1.641597 | 0.003156 | *ZNF236* | 1.621751 | 0.000204 |
| *CAMK2D* | 1.641221 | 0.001951 | *C8orf58* | 1.620254 | 0.003448 |
| *ATG14* | 1.635294 | 0.001914 | *LOC100131564* | 1.618591 | 0.02057 |
| *SFTA1P* | 1.632117 | 0.002417 | *PBXIP1* | 1.614787 | 0.000526 |
| *HCFC1R1* | 1.63201 | 0.00256 | *VPS39* | 1.613586 | 0.000223 |
| *CACNA2D2* | 1.63041 | 0.001765 | *TM7SF2* | 1.610128 | 0.001086 |
| *CLYBL* | 1.62921 | 0.004429 | *LOC101928530* | 1.605348 | 0.001567 |
| *PVRL3* | 1.627121 | 0.002413 | *SELM* | 1.602587 | 0.002685 |
| *CDR2L* | 1.6261 | 0.003363 | *LOC100507577* | 1.601428 | 0.002654 |
| *PRKCA* | 1.620107 | 0.001696 | *JMJD1C-AS1* | 1.599426 | 0.000858 |
| *HPCAL1* | 1.617315 | 0.002266 | *SLC25A37* | 1.598765 | 0.000451 |
| *CYB5R3* | 1.616004 | 0.004591 | *HPCAL1* | 1.598231 | 0.000757 |
| *ASAP2* | 1.615484 | 0.001567 | *ASAP2* | 1.587482 | 0.000614 |
| *SCD5* | 1.615141 | 0.00424 | *ZNF185* | 1.578965 | 0.000386 |
| *CD99L2* | 1.613478 | 0.004734 | *PTPRS* | 1.577176 | 0.000408 |
| *MAP2K6* | 1.605879 | 0.013805 | *TFAP2C* | 1.576314 | 0.001884 |
| *PC* | 1.604411 | 0.022318 | *ST3GAL2* | 1.575031 | 0.007426 |
| *SPIRE1* | 1.601389 | 0.001552 | *TSHZ1* | 1.573903 | 0.001827 |
| *SOBP* | 1.599318 | 0.005053 | *FAM214B* | 1.568617 | 0.004022 |
| *LRP1* | 1.597352 | 0.002951 | *CTSF* | 1.568571 | 0.006484 |
| *SEC24D* | 1.592008 | 0.001603 | *CACNA2D2* | 1.56648 | 0.000643 |
| *LNP1* | 1.585252 | 0.003515 | *RTTN* | 1.563454 | 0.000354 |
| *SCARA3* | 1.581345 | 0.004328 | *ADPRHL1* | 1.562987 | 0.000775 |
| *FERMT2* | 1.575955 | 0.001955 | *ACOT2* | 1.562552 | 0.001835 |
| *MIR24-2* | 1.571492 | 0.019477 | *ZSWIM8* | 1.561147 | 0.000535 |
| *SLC25A37* | 1.570907 | 0.002518 | *CXXC1* | 1.557459 | 0.000969 |
| *LOC100131564* | 1.566296 | 0.041762 | *SMAD2* | 1.557228 | 0.001452 |
| *RNF125* | 1.566109 | 0.003166 | *LOC100132356* | 1.549124 | 0.000933 |
| *GLT8D1* | 1.564316 | 0.003378 | *LOC100506100* | 1.548004 | 0.001536 |
| *FNBP1* | 1.563623 | 0.003164 | *PVRL3* | 1.542448 | 0.000687 |
| *ZC3H6* | 1.561852 | 0.02595 | *CHST7* | 1.540714 | 0.000598 |
| *HSPG2* | 1.561506 | 0.003945 | *ORAI3* | 1.538541 | 0.000995 |
| *BTN2A2* | 1.553148 | 0.006838 | *SEC11C* | 1.537967 | 0.000816 |
| *PSEN1* | 1.549777 | 0.002802 | *SEMA4G* | 1.536228 | 0.002607 |
| *SOCS6* | 1.546395 | 0.010656 | *LOC101928674* | 1.534689 | 0.001829 |
| *TRAPPC8* | 1.543174 | 0.008696 | *SORBS3* | 1.534049 | 0.001342 |
| *B4GALT6* | 1.541437 | 0.005163 | *CITED2* | 1.526805 | 0.005299 |
| *IFI6* | 1.539199 | 0.006736 | *MLLT11* | 1.525873 | 0.006374 |
| *TSHZ1* | 1.537969 | 0.030291 | *LMAN1* | 1.523495 | 0.000648 |
| *PPARA* | 1.536575 | 0.007787 | *BTN2A2* | 1.522355 | 0.001552 |
| *LOC100506844* | 1.532356 | 0.005934 | *TMX4* | 1.521442 | 0.000732 |
| *GALNT1* | 1.532248 | 0.024917 | *HABP4* | 1.521427 | 0.000641 |
| *FAM210B* | 1.527925 | 0.012119 | *RBM24* | 1.519481 | 0.001642 |
| *MICU3* | 1.524603 | 0.047704 | *CTNS* | 1.516861 | 0.003101 |
| *ZFYVE26* | 1.524242 | 0.00795 | *CARD8* | 1.51558 | 0.00942 |
| *CARD8* | 1.521013 | 0.00893 | *SOCS6* | 1.514276 | 0.002612 |
| *CTNS* | 1.517439 | 0.010939 | *TNFRSF10C* | 1.513416 | 0.015883 |
| *ACOT2* | 1.51637 | 0.006208 | *MAGED1* | 1.513188 | 0.000697 |
| *ST3GAL2* | 1.515147 | 0.006603 | *FRMD6-AS1* | 1.512259 | 0.002826 |
| *ATP8B3* | 1.510661 | 0.01658 | *PPARA* | 1.510869 | 0.009966 |
| *LOC101927365* | 1.510233 | 0.031109 | *MBD1* | 1.510495 | 0.00185 |
| *FRMD6-AS1* | 1.509832 | 0.0098 | *TP53INP1* | 1.508956 | 0.005627 |
| *VPS39* | 1.509351 | 0.006736 | *SAMD4A* | 1.507937 | 0.001014 |
| *C18orf54* | 1.508307 | 0.027482 | *CHKA* | 1.507786 | 0.000764 |
| *CHST7* | 1.506773 | 0.00576 | *LOC101928868* | 1.507618 | 0.002494 |
| *C8orf58* | 1.503999 | 0.028494 | *PLEKHG4* | 1.505893 | 0.005195 |
| *SAMD4A* | 1.501027 | 0.008483 | *KLF12* | 1.503829 | 0.000862 |
| **COMMON DOWN-REGULATED GENES in DR vs. PARENTALS** | | | | | |
| **DU-145DR vs. DU-145** | | | **PC-3DR vs. PC-3** | | |
| **Gene Symbol** | **FC** | **FDR** | **Gene Symbol** | **FC** | **FDR** |
| *MAL2* | -133.807 | 1.02E-09 | *ESRP1* | -92.8716 | 5.13E-09 |
| *AFAP1-AS1* | -39.9853 | 2.65E-08 | *EFEMP1* | -48.7116 | 2.19E-09 |
| *ESRP1* | -34.3019 | 2.38E-08 | *LCP1* | -44.2875 | 5.2E-08 |
| *NRG4* | -23.114 | 1.55E-07 | *MPZL2* | -42.6202 | 1.03E-08 |
| *EPCAM* | -21.0433 | 2.65E-08 | *CDH1* | -40.993 | 4.73E-09 |
| *TC2N* | -17.2825 | 1.47E-06 | *TC2N* | -37.0216 | 1.49E-08 |
| *CDH1* | -16.5908 | 1.55E-07 | *OCLN* | -32.9964 | 3.22E-07 |
| *MPZL2* | -15.6517 | 1.58E-07 | *ST14* | -25.9496 | 3.26E-08 |
| *TACSTD2* | -13.6473 | 2.48E-06 | *GJB2* | -25.5639 | 9.72E-06 |
| *TNS4* | -12.348 | 7.06E-08 | *PTGFRN* | -20.4589 | 3.01E-08 |
| *SERPINB5* | -10.6812 | 1.47E-06 | *EPCAM* | -20.1467 | 7.03E-09 |
| *GALNT3* | -10.414 | 5.76E-07 | *TACSTD2* | -19.4003 | 3.45E-08 |
| *ST14* | -10.2692 | 1.69E-06 | *SLC27A2* | -17.9332 | 4.05E-07 |
| *SPINT1* | -10.0563 | 5.16E-07 | *FAM84B* | -15.117 | 8.37E-09 |
| *SERPINA1* | -9.24011 | 5.76E-06 | *TMEM30B* | -14.9063 | 1.08E-08 |
| *GPR87* | -9.19183 | 8.54E-07 | *SLC1A3* | -14.2764 | 1.28E-07 |
| *C1orf116* | -6.94866 | 8.98E-07 | *CLDN7* | -14.1886 | 1.32E-08 |
| *MME* | -6.93414 | 1.33E-06 | *TMEM45B* | -14.0313 | 1.55E-07 |
| *GJA1* | -6.49417 | 1.13E-06 | *SERPINA1* | -12.4644 | 1.83E-07 |
| *CD274* | -6.47177 | 5.76E-07 | *CADM1* | -12.1482 | 3.45E-08 |
| *PADI2* | -6.21266 | 2.48E-06 | *FBP1* | -11.9198 | 2.72E-08 |
| *LAMC2* | -6.20208 | 4.56E-07 | *DPYD* | -11.5024 | 2.55E-08 |
| *BMP4* | -6.17783 | 1.69E-06 | *TXNIP* | -11.0485 | 2.65E-06 |
| *VAMP8* | -6.13961 | 1.1E-06 | *MACC1* | -11.0359 | 1.19E-07 |
| *VWDE* | -5.80914 | 9.22E-07 | *PRRG4* | -10.4533 | 2.88E-08 |
| *TMEM156* | -5.78794 | 9.91E-07 | *LCN2* | -9.99246 | 1.42E-07 |
| *DDIT4* | -5.72815 | 1.39E-06 | *LAD1* | -9.68137 | 2.72E-08 |
| *JPH1* | -5.42319 | 2.21E-05 | *CDS1* | -9.38107 | 2.72E-08 |
| *CALD1* | -5.19535 | 1.92E-05 | *ATP2C2* | -8.27743 | 2.71E-07 |
| *TMEM45B* | -5.1878 | 3.94E-06 | *ARAP2* | -8.11344 | 4.66E-07 |
| *MTUS1* | -4.95193 | 1.39E-06 | *C2orf15* | -7.82578 | 1.11E-06 |
| *SLC1A3* | -4.83215 | 3.9E-06 | *DDIT4* | -7.47767 | 1.77E-05 |
| *TXNIP* | -4.66709 | 7.52E-05 | *GALNT3* | -7.44095 | 4.52E-08 |
| *RNF43* | -4.62104 | 5.48E-06 | *LOC647859* | -6.83117 | 7.17E-07 |
| *CST6* | -4.61972 | 3.89E-06 | *CLDN1* | -6.79674 | 1.33E-06 |
| *MIR622* | -4.54705 | 1.06E-05 | *SMPDL3B* | -6.63568 | 1.2E-07 |
| *RAB17* | -4.45025 | 0.000101 | *JPH1* | -6.39564 | 2.49E-07 |
| *SYK* | -4.2379 | 1.69E-06 | *TNS4* | -6.35728 | 3.64E-07 |
| *CASP4* | -4.17151 | 3.89E-06 | *BMP4* | -6.09643 | 4.66E-07 |
| *SPINT2* | -4.16085 | 1.47E-06 | *MBNL3* | -6.01422 | 1.19E-07 |
| *FAM84B* | -4.05188 | 2.69E-05 | *KRT7* | -5.97379 | 4.49E-07 |
| *MAP7* | -3.83573 | 9.36E-06 | *IRF6* | -5.93204 | 8.48E-08 |
| *EREG* | -3.81433 | 4.81E-06 | *SPINT1* | -5.84111 | 1.2E-07 |
| *SMPDL3B* | -3.79954 | 9.78E-06 | *LSR* | -5.79363 | 8.99E-08 |
| *ASNS* | -3.76244 | 2.53E-06 | *MOXD1* | -5.74625 | 1.21E-06 |
| *SCN9A* | -3.7355 | 0.000105 | *PLEKHG1* | -5.72003 | 0.000115 |
| *PRICKLE1* | -3.57826 | 9.34E-06 | *TBC1D30* | -5.58822 | 1.35E-07 |
| *DENND2D* | -3.49981 | 3.28E-05 | *CD274* | -5.4858 | 1.33E-06 |
| *SEMA3E* | -3.49817 | 2.33E-05 | *PRR15* | -5.45875 | 6.44E-07 |
| *DMKN* | -3.49786 | 9.69E-06 | *C1orf116* | -5.43854 | 3E-06 |
| *GJB2* | -3.46257 | 2.21E-05 | *MARVELD2* | -5.40381 | 1.49E-06 |
| *SEL1L3* | -3.43933 | 6.03E-06 | *MARVELD3* | -5.37975 | 1.92E-07 |
| *IFIH1* | -3.43315 | 1.31E-05 | *ZNF165* | -5.29571 | 1.54E-07 |
| *KRTAP2-3* | -3.40724 | 2.48E-05 | *DLC1* | -5.20275 | 1.24E-06 |
| *CDS1* | -3.3483 | 6.09E-06 | *SYK* | -5.00905 | 1.78E-06 |
| *GUCY1B3* | -3.27077 | 4.83E-06 | *MAPK13* | -4.92182 | 2.33E-07 |
| *CGN* | -3.20102 | 2.66E-05 | *HOOK1* | -4.85295 | 2.25E-07 |
| *TAOK3* | -3.18542 | 4.81E-06 | *MPZL3* | -4.77396 | 1.54E-06 |
| *HOXB9* | -3.18381 | 6.82E-06 | *CHMP4C* | -4.76641 | 3.64E-07 |
| *MAPK13* | -3.17783 | 8.47E-06 | *MAP7* | -4.72967 | 1.72E-07 |
| *PLA2G4A* | -3.16118 | 2.21E-05 | *SCN9A* | -4.68324 | 8.99E-06 |
| *ABHD17C* | -3.10072 | 7.22E-05 | *NNMT* | -4.65556 | 7.28E-05 |
| *LAD1* | -3.08294 | 0.000292 | *GPR87* | -4.57107 | 2.81E-07 |
| *VDR* | -3.08201 | 2.66E-05 | *ARHGDIB* | -4.48226 | 3.99E-06 |
| *FBXO32* | -3.06583 | 0.00019 | *SLC37A1* | -4.30174 | 7.1E-07 |
| *ANK3* | -3.0644 | 6.34E-06 | *TINAGL1* | -4.18715 | 4.68E-06 |
| *MARVELD3* | -3.0133 | 1.42E-05 | *SNORD93* | -4.10873 | 0.000627 |
| *GDAP1* | -2.96108 | 0.000114 | *ATP10A* | -4.06398 | 5.09E-06 |
| *GOLT1A* | -2.94672 | 0.000314 | *ANKH* | -4.05281 | 1.13E-06 |
| *FRMD4A* | -2.91882 | 5.62E-05 | *HOXB9* | -4.05103 | 0.000203 |
| *KIAA1244* | -2.90676 | 5.06E-05 | *MNS1* | -3.97936 | 1.45E-06 |
| *TINAGL1* | -2.87939 | 0.000155 | *ST8SIA4* | -3.94314 | 1.22E-05 |
| *SYTL2* | -2.8613 | 0.000113 | *INADL* | -3.81535 | 7.11E-07 |
| *MOB3B* | -2.83271 | 4.64E-05 | *C6orf132* | -3.78654 | 1.1E-06 |
| *PLEKHG1* | -2.82129 | 7.67E-05 | *WWC1* | -3.71216 | 5.45E-07 |
| *CSF2RA* | -2.80796 | 0.000822 | *BSPRY* | -3.68149 | 1.71E-05 |
| *MARVELD2* | -2.80455 | 2.29E-05 | *SH2D3A* | -3.68068 | 2.93E-05 |
| *C2orf15* | -2.80278 | 0.000173 | *VEGFA* | -3.65556 | 5.48E-05 |
| *CLDN7* | -2.75648 | 7.39E-05 | *MYO6* | -3.62103 | 1.12E-06 |
| *RPL22L1* | -2.75527 | 2.69E-05 | *IFIH1* | -3.54345 | 9.67E-06 |
| *FAM222A-AS1* | -2.74696 | 0.00085 | *CDC42EP3* | -3.54291 | 8.3E-06 |
| *EFEMP1* | -2.73903 | 1.35E-05 | *TGM2* | -3.49288 | 1.12E-06 |
| *APOBEC3D* | -2.73877 | 0.000354 | *WSCD1* | -3.45166 | 6.34E-07 |
| *ATP10A* | -2.7279 | 0.000141 | *VDR* | -3.39929 | 2.32E-06 |
| *BCAT1* | -2.71426 | 0.000203 | *C17orf51* | -3.33237 | 9.93E-07 |
| *MND1* | -2.71007 | 2.16E-05 | *ICA1* | -3.23798 | 3.86E-06 |
| *CBLC* | -2.67373 | 0.000412 | *RNF43* | -3.21518 | 4.32E-05 |
| *MNS1* | -2.67095 | 6.06E-05 | *MOCOS* | -3.1787 | 1.17E-05 |
| *SLC27A2* | -2.64358 | 4.79E-05 | *FAM111B* | -3.17294 | 0.000101 |
| *GLIPR1* | -2.60299 | 8.26E-05 | *RPS6KA2* | -3.16748 | 1.98E-05 |
| *TJP2* | -2.59453 | 3.65E-05 | *RBM47* | -3.10014 | 1.8E-05 |
| *IRF6* | -2.59196 | 5.9E-05 | *ASNS* | -3.0995 | 0.000366 |
| *SAMD12* | -2.57224 | 0.000537 | *KDF1* | -3.04046 | 4.89E-06 |
| *CYFIP2* | -2.56958 | 6.65E-05 | *FAM111A* | -3.03394 | 2.3E-06 |
| *RAB19* | -2.56683 | 8.21E-05 | *AMIGO2* | -3.02979 | 0.000592 |
| *FA2H* | -2.55362 | 0.000772 | *SNORD72* | -3.02686 | 2.51E-05 |
| *WSCD1* | -2.54951 | 6.1E-05 | *GTPBP10* | -3.01423 | 2.96E-06 |
| *MREG* | -2.53549 | 0.000152 | *ABHD17C* | -2.96775 | 3.46E-06 |
| *FAM160A1* | -2.52061 | 8.35E-05 | *CRB3* | -2.92622 | 3.34E-05 |
| *BSPRY* | -2.51392 | 3.72E-05 | *PMEPA1* | -2.90626 | 8.22E-06 |
| *NIPAL1* | -2.49027 | 0.00068 | *GABRQ* | -2.90554 | 9.61E-06 |
| *SFMBT2* | -2.48513 | 7.15E-05 | *SFMBT2* | -2.89859 | 2.03E-05 |
| *RIPK4* | -2.48296 | 0.000561 | *KRTAP2-3* | -2.8875 | 0.000122 |
| *GATA6* | -2.45736 | 6.89E-05 | *SATB1* | -2.88009 | 2.89E-06 |
| *MAP3K1* | -2.4571 | 7.54E-05 | *HOXC8* | -2.85096 | 6.7E-06 |
| *GALNT12* | -2.44152 | 4.54E-05 | *TRIB3* | -2.83418 | 0.00018 |
| *KIAA0922* | -2.41307 | 8.27E-05 | *SEMA3E* | -2.82771 | 2.46E-05 |
| *PRRG4* | -2.40459 | 0.000265 | *GUCY1B3* | -2.82158 | 1.31E-05 |
| *HOXB2* | -2.3888 | 0.000227 | *MIR622* | -2.79525 | 2.89E-06 |
| *RPS6KA2* | -2.37103 | 0.001046 | *PRAMEF25* | -2.75783 | 0.002492 |
| *SH2D3A* | -2.37082 | 0.000272 | *RTKN2* | -2.75721 | 7.15E-06 |
| *CDC42EP3* | -2.36354 | 7.99E-05 | *LOC101927204* | -2.7335 | 2.28E-05 |
| *SLC12A8* | -2.36039 | 0.000203 | *RASEF* | -2.7248 | 6.75E-06 |
| *APOL6* | -2.35999 | 0.000551 | *RIPK4* | -2.71788 | 3.92E-06 |
| *HSPB8* | -2.35966 | 0.002382 | *ESRP2* | -2.70843 | 2.91E-06 |
| *PARP12* | -2.35741 | 0.00017 | *FAM83H* | -2.70014 | 2.63E-06 |
| *CXCL16* | -2.3565 | 0.000128 | *CXCL16* | -2.6981 | 2.29E-06 |
| *KRT7* | -2.35571 | 0.000386 | *VAMP8* | -2.69455 | 9.3E-06 |
| *IFI16* | -2.35413 | 0.000332 | *NIPAL1* | -2.67629 | 3.99E-06 |
| *SH3RF1* | -2.34502 | 0.000137 | *EGLN3* | -2.67331 | 4.09E-05 |
| *GLS2* | -2.32029 | 4.38E-05 | *SNHG8* | -2.66463 | 6.45E-05 |
| *NMI* | -2.31679 | 0.000111 | *TJP3* | -2.65058 | 9.41E-06 |
| *ZNF165* | -2.31251 | 0.000296 | *SH3BGRL2* | -2.64132 | 3.62E-06 |
| *CSGALNACT1* | -2.3101 | 0.000181 | *RBMS3* | -2.62654 | 7.52E-05 |
| *SNORA4* | -2.29015 | 0.001031 | *ALDH1A3* | -2.61674 | 4.4E-06 |
| *FGD4* | -2.24213 | 0.000164 | *MIR891A* | -2.6028 | 0.000253 |
| *PTGFRN* | -2.23023 | 0.00015 | *KIAA0922* | -2.51474 | 4E-05 |
| *MBNL3* | -2.20608 | 0.000412 | *SAMD12* | -2.51329 | 7.12E-06 |
| *AMIGO2* | -2.20581 | 0.000175 | *SNORA26* | -2.50388 | 9.14E-06 |
| *MISP* | -2.18027 | 0.000225 | *BCAT1* | -2.49593 | 3.09E-05 |
| *MOXD1* | -2.16952 | 0.001824 | *DERA* | -2.49383 | 1.11E-05 |
| *SERINC2* | -2.15927 | 0.000277 | *MANSC1* | -2.47703 | 9.65E-06 |
| *PPP1R14C* | -2.14428 | 0.000595 | *C4orf19* | -2.46562 | 0.000197 |
| *HERC6* | -2.14382 | 0.000564 | *SESN2* | -2.45589 | 0.000905 |
| *LCN2* | -2.14242 | 0.006977 | *CLMP* | -2.45509 | 3.88E-06 |
| *HOXC8* | -2.1424 | 0.000139 | *ARRDC4* | -2.45493 | 0.000249 |
| *TFCP2L1* | -2.14159 | 0.000227 | *ROR1* | -2.42589 | 8.54E-06 |
| *AP1S3* | -2.13428 | 0.000401 | *SLC7A5* | -2.41782 | 4.04E-05 |
| *ALDH1A3* | -2.12679 | 0.000348 | *MYB* | -2.40036 | 0.000109 |
| *FAM111A* | -2.12634 | 0.00015 | *SLC12A8* | -2.39337 | 1.57E-05 |
| *CADPS2* | -2.1193 | 0.000216 | *HSPB8* | -2.37199 | 0.000165 |
| *LOC101927204* | -2.10438 | 0.00124 | *C19orf48* | -2.36998 | 3.13E-05 |
| *RPS26* | -2.10241 | 0.003315 | *ELOVL7* | -2.35784 | 0.000122 |
| *RBM47* | -2.08295 | 0.000279 | *IL17RB* | -2.35082 | 0.000138 |
| *THAP10* | -2.07944 | 0.000135 | *PDGFC* | -2.34833 | 1.06E-05 |
| *TPD52L1* | -2.07138 | 0.000199 | *TAOK3* | -2.34807 | 3.57E-05 |
| *FAM111B* | -2.06853 | 0.00158 | *TFCP2L1* | -2.34169 | 3.72E-05 |
| *ARHGAP28* | -2.06848 | 0.002163 | *DYSF* | -2.31981 | 1.32E-05 |
| *PRR15* | -2.05812 | 0.000645 | *ARHGAP10* | -2.30849 | 8.7E-05 |
| *ZNF525* | -2.05677 | 0.036038 | *IFI16* | -2.28329 | 1E-04 |
| *MYB* | -2.05526 | 0.000501 | *FA2H* | -2.2822 | 3.5E-05 |
| *RAB11FIP4* | -2.04484 | 0.00159 | *CGN* | -2.28102 | 6.38E-05 |
| *REPS2* | -2.04045 | 0.001178 | *VWDE* | -2.28099 | 1.01E-05 |
| *TMEM238* | -2.03935 | 0.002083 | *TMEM154* | -2.25651 | 0.001379 |
| *GAL* | -2.03299 | 0.000295 | *AFAP1-AS1* | -2.2565 | 0.00049 |
| *CRB3* | -2.02297 | 0.003723 | *MIR4521* | -2.25358 | 4E-05 |
| *TMEM30B* | -2.01085 | 0.002808 | *PLA2G4A* | -2.25234 | 0.000224 |
| *CLDN1* | -1.99671 | 0.00019 | *SNX10* | -2.24826 | 0.001174 |
| *SFXN2* | -1.99481 | 0.000443 | *STYK1* | -2.23232 | 0.000187 |
| *KDF1* | -1.9852 | 0.000883 | *RAB17* | -2.22892 | 0.000105 |
| *NOP16* | -1.98416 | 0.000403 | *SEPP1* | -2.20934 | 0.00149 |
| *SNORA13* | -1.97947 | 0.001527 | *PARP12* | -2.19584 | 9.54E-06 |
| *EGLN3* | -1.97313 | 0.000586 | *ARHGAP28* | -2.18971 | 0.000578 |
| *MCOLN3* | -1.96412 | 0.003661 | *MND1* | -2.18148 | 3.57E-05 |
| *ELL3* | -1.95747 | 0.000272 | *FGD4* | -2.18076 | 0.000593 |
| *MIR4640* | -1.95353 | 0.01268 | *RPARP-AS1* | -2.17909 | 5.16E-05 |
| *ELOVL7* | -1.94082 | 0.002091 | *PBX1* | -2.1731 | 2.98E-05 |
| *ESRP2* | -1.93954 | 0.000843 | *TBC1D2* | -2.16537 | 0.000415 |
| *PDCD1LG2* | -1.9394 | 0.002539 | *SERINC2* | -2.15892 | 1.67E-05 |
| *ARHGAP44* | -1.93833 | 0.00103 | *CSF2RA* | -2.1571 | 0.000101 |
| *KRT18* | -1.9374 | 0.000198 | *ADIPOR2* | -2.14469 | 0.000316 |
| *MIR891A* | -1.93671 | 0.003965 | *PADI2* | -2.14064 | 5.9E-05 |
| *IDS* | -1.93621 | 0.000316 | *GPR160* | -2.12631 | 6.88E-05 |
| *ATP2C2* | -1.9352 | 0.000925 | *AP1S3* | -2.12413 | 4.36E-05 |
| *MANSC1* | -1.93454 | 0.000644 | *TPD52L1* | -2.11623 | 3.61E-05 |
| *MMD* | -1.92456 | 0.00038 | *SFXN2* | -2.11349 | 0.000164 |
| *GRPEL2* | -1.92333 | 0.000514 | *DDR1* | -2.10679 | 1.23E-05 |
| *SLFN5* | -1.92073 | 0.000959 | *ZNF525* | -2.10569 | 0.021229 |
| *C19orf48* | -1.91849 | 0.000797 | *MLLT3* | -2.09028 | 4.09E-05 |
| *TRIB3* | -1.91723 | 0.000478 | *CADPS2* | -2.08799 | 0.00015 |
| *ARAP2* | -1.91427 | 0.003497 | *RIBC2* | -2.08296 | 0.000324 |
| *GRB7* | -1.90454 | 0.000786 | *JUP* | -2.07705 | 2.22E-05 |
| *AIM1* | -1.90405 | 0.000314 | *MYO5B* | -2.07239 | 0.000485 |
| *HNRNPAB* | -1.90066 | 0.000551 | *SCNN1A* | -2.06948 | 4.87E-05 |
| *LINC00673* | -1.89884 | 0.000825 | *SOX4* | -2.06638 | 2.79E-05 |
| *RTKN2* | -1.89728 | 0.001253 | *FAM222A-AS1* | -2.05998 | 0.000126 |
| *MRPL35* | -1.89698 | 0.000366 | *SNHG12* | -2.05492 | 9.65E-05 |
| *ARHGAP10* | -1.89199 | 0.000609 | *PPP1R14C* | -2.05451 | 0.000199 |
| *SNORA26* | -1.89051 | 0.004239 | *ANK3* | -2.048 | 2.93E-05 |
| *OCLN* | -1.88796 | 0.000843 | *ARHGAP44* | -2.0444 | 2.85E-05 |
| *YBX3P1* | -1.87418 | 0.000581 | *EPS8L1* | -2.03628 | 6.47E-05 |
| *RAPGEF5* | -1.86995 | 0.002993 | *GJA1* | -2.02675 | 0.000132 |
| *C12orf45* | -1.86672 | 0.001908 | *TMEM238* | -2.02447 | 3.73E-05 |
| *SLC37A1* | -1.86549 | 0.000843 | *AIG1* | -2.02261 | 5.27E-05 |
| *POLR3B* | -1.86241 | 0.000654 | *FBXL14* | -2.01993 | 4.26E-05 |
| *HOOK1* | -1.85826 | 0.006106 | *LYPD3* | -2.019 | 7.45E-05 |
| *MACC1* | -1.85223 | 0.001877 | *MAL2* | -2.00984 | 2.28E-05 |
| *TMEM154* | -1.8511 | 0.003548 | *MISP* | -1.99862 | 8.27E-05 |
| *SATB1* | -1.85061 | 0.005384 | *FAS* | -1.99502 | 0.000303 |
| *PBX1* | -1.84889 | 0.014354 | *LGALS8* | -1.99236 | 3.88E-05 |
| *LRBA* | -1.84864 | 0.000374 | *ZSCAN12P1* | -1.98393 | 0.000132 |
| *SSBP1* | -1.84619 | 0.000366 | *HIST4H4* | -1.97953 | 3.94E-05 |
| *CAMSAP3* | -1.83787 | 0.003246 | *GRPEL2* | -1.9746 | 0.000191 |
| *FAM83H* | -1.83259 | 0.00103 | *MOB3B* | -1.96743 | 0.000112 |
| *SH3BGRL2* | -1.8324 | 0.001161 | *EZH2* | -1.96279 | 0.000125 |
| *SLC7A5* | -1.83128 | 0.000709 | *SEMA6B* | -1.96098 | 4.68E-05 |
| *LOC101927391* | -1.80912 | 0.008471 | *RAB19* | -1.95636 | 0.000199 |
| *TBC1D30* | -1.80584 | 0.003132 | *LIPG* | -1.95583 | 0.000404 |
| *GPR160* | -1.80484 | 0.005006 | *PDCD1LG2* | -1.94828 | 0.000457 |
| *TBC1D2* | -1.80317 | 0.002797 | *NABP1* | -1.94331 | 8.08E-05 |
| *PMEPA1* | -1.79263 | 0.001543 | *TMEM54* | -1.93542 | 0.000105 |
| *EZH2* | -1.78566 | 0.00148 | *MIR3198-1* | -1.93042 | 0.000101 |
| *ST8SIA4* | -1.78444 | 0.000935 | *MTUS1* | -1.92448 | 0.000234 |
| *FBP1* | -1.78169 | 0.003162 | *RAPGEF5* | -1.91881 | 0.000102 |
| *SNX10* | -1.78 | 0.001229 | *ZNF350* | -1.9171 | 0.00021 |
| *SNHG8* | -1.77892 | 0.000636 | *LINC00673* | -1.90849 | 3.79E-05 |
| *DCP1B* | -1.77149 | 0.001123 | *SLFN5* | -1.90665 | 0.001486 |
| *NNMT* | -1.77074 | 0.000583 | *SNORA22* | -1.9006 | 0.001893 |
| *SNHG4* | -1.76499 | 0.032404 | *BID* | -1.90001 | 4.25E-05 |
| *MRPL39* | -1.76479 | 0.00098 | *PRICKLE1* | -1.86742 | 0.000695 |
| *EPS8L1* | -1.76269 | 0.001037 | *FHDC1* | -1.86731 | 0.001567 |
| *SFN* | -1.76235 | 0.003087 | *ARHGAP18* | -1.85668 | 7.16E-05 |
| *GABRQ* | -1.76217 | 0.004453 | *SNORA4* | -1.85153 | 0.002306 |
| *SEPP1* | -1.7617 | 0.001877 | *CSGALNACT1* | -1.83775 | 0.001178 |
| *DDR1* | -1.75974 | 0.001596 | *FRMD5* | -1.83557 | 0.000163 |
| *FRMD5* | -1.75884 | 0.000798 | *GLS2* | -1.83154 | 0.000231 |
| *PRAMEF25* | -1.75411 | 0.004679 | *CBLC* | -1.82641 | 0.001174 |
| *SESN2* | -1.74033 | 0.00227 | *KIAA1244* | -1.82639 | 0.000287 |
| *ZNF641* | -1.73611 | 0.001342 | *PPCDC* | -1.82291 | 0.000101 |
| *HIST4H4* | -1.73434 | 0.001547 | *LAT2* | -1.81608 | 0.001216 |
| *MYO6* | -1.73343 | 0.00612 | *MREG* | -1.81588 | 0.000594 |
| *ANKH* | -1.73087 | 0.00098 | *KRT18* | -1.81439 | 0.000128 |
| *FBXL14* | -1.72602 | 0.000667 | *DCP1B* | -1.80978 | 5.23E-05 |
| *STYK1* | -1.72508 | 0.003189 | *LOC647264* | -1.80583 | 0.000522 |
| *WWC1* | -1.72095 | 0.001303 | *SH3RF1* | -1.79917 | 0.000232 |
| *AIG1* | -1.71951 | 0.000744 | *EGFR* | -1.79729 | 0.000137 |
| *ROR1* | -1.71909 | 0.001511 | *DOPEY1* | -1.79438 | 0.000122 |
| *RPARP-AS1* | -1.71379 | 0.001123 | *APOBEC3C* | -1.78843 | 9.34E-05 |
| *BLM* | -1.71351 | 0.003363 | *AIM1* | -1.78491 | 0.000148 |
| *MIR3198-1* | -1.71223 | 0.015235 | *CCNB1* | -1.77632 | 0.000278 |
| *FHDC1* | -1.70739 | 0.007684 | *KRT80* | -1.76979 | 0.000141 |
| *LOC647859* | -1.70367 | 0.001184 | *TMEM156* | -1.76854 | 0.000478 |
| *CLMP* | -1.7035 | 0.004312 | *SNHG4* | -1.76438 | 0.000816 |
| *TMEM5* | -1.69855 | 0.002512 | *MARS* | -1.75668 | 0.000285 |
| *DERA* | -1.69703 | 0.000911 | *NMI* | -1.75558 | 0.000291 |
| *LYPD3* | -1.69575 | 0.003697 | *POLR3B* | -1.75448 | 0.000158 |
| *C17orf51* | -1.68971 | 0.004194 | *TSTD1* | -1.74844 | 0.00087 |
| *C6orf132* | -1.68794 | 0.00402 | *ELL3* | -1.74574 | 0.000598 |
| *TJP3* | -1.6847 | 0.012109 | *ZNF774* | -1.74482 | 0.000132 |
| *CCNB1* | -1.68419 | 0.003996 | *HOXB2* | -1.74305 | 0.007543 |
| *LMNB1* | -1.67786 | 0.001182 | *SPINT2* | -1.74177 | 8.76E-05 |
| *TGM2* | -1.67771 | 0.001659 | *GLIPR1* | -1.73944 | 0.000264 |
| *LRRC61* | -1.67764 | 0.001185 | *LRRC61* | -1.73902 | 0.000241 |
| *ADIPOR2* | -1.67753 | 0.001448 | *DENND2D* | -1.72554 | 0.000161 |
| *VEGFA* | -1.67422 | 0.000911 | *MME* | -1.7243 | 0.005178 |
| *CDCA3* | -1.67391 | 0.000995 | *YBX3P1* | -1.71994 | 0.000281 |
| *ERMP1* | -1.67108 | 0.001231 | *RPL22L1* | -1.70982 | 0.005272 |
| *MIR181A1HG* | -1.66546 | 0.00795 | *ZNF551* | -1.70904 | 0.00041 |
| *GTPBP10* | -1.66497 | 0.00457 | *PKP3* | -1.70894 | 0.001918 |
| *RIBC2* | -1.66135 | 0.003559 | *LY6E* | -1.707 | 0.000147 |
| *NEIL3* | -1.65906 | 0.002067 | *KLHL2* | -1.70302 | 0.000291 |
| *ARHGDIB* | -1.65712 | 0.011503 | *ERMP1* | -1.70095 | 0.000203 |
| *DOPEY1* | -1.6507 | 0.029361 | *MRPL35* | -1.69613 | 0.000236 |
| *ZNF350* | -1.64945 | 0.003055 | *CD40* | -1.69087 | 0.002408 |
| *ZNF774* | -1.64338 | 0.004922 | *ZNF641* | -1.68387 | 0.001537 |
| *SNX24* | -1.64172 | 0.005298 | *FRMD4A* | -1.67972 | 0.004073 |
| *ICA1* | -1.64162 | 0.001454 | *SNORA13* | -1.67798 | 0.00036 |
| *LCP1* | -1.63979 | 0.019017 | *APOL6* | -1.67775 | 0.000547 |
| *DYSF* | -1.63961 | 0.001648 | *SNX24* | -1.677 | 0.000445 |
| *TSTD1* | -1.63574 | 0.004047 | *RPL36* | -1.67393 | 0.000308 |
| *TUBE1* | -1.63465 | 0.015485 | *CCDC71L* | -1.67389 | 0.000232 |
| *MIR4521* | -1.63452 | 0.01208 | *TTC22* | -1.67357 | 0.003694 |
| *MLLT3* | -1.63432 | 0.002518 | *GCH1* | -1.66979 | 0.000222 |
| *RASEF* | -1.63266 | 0.00257 | *DMKN* | -1.66949 | 0.000234 |
| *MOCOS* | -1.62512 | 0.003148 | *LAMC2* | -1.6668 | 0.000248 |
| *FAS* | -1.62222 | 0.001669 | *CASP4* | -1.66248 | 0.000243 |
| *LIPG* | -1.62076 | 0.003096 | *GDAP1* | -1.66132 | 0.001209 |
| *APOBEC3C* | -1.61894 | 0.008267 | *MEST* | -1.65943 | 0.000131 |
| *ZNF551* | -1.61847 | 0.003795 | *FAM160A1* | -1.65934 | 0.000418 |
| *ACSL1* | -1.6184 | 0.003187 | *SLC52A3* | -1.65913 | 0.000856 |
| *LAT2* | -1.61736 | 0.00408 | *IDS* | -1.65667 | 0.00024 |
| *NMB* | -1.6173 | 0.003315 | *GRB7* | -1.65443 | 0.00068 |
| *CHMP4C* | -1.61685 | 0.003248 | *SH3RF2* | -1.65387 | 0.000276 |
| *FUT1* | -1.61676 | 0.020529 | *RPS26* | -1.65099 | 0.009983 |
| *MYO5B* | -1.61278 | 0.007189 | *CGNL1* | -1.64991 | 0.002087 |
| *PIP4K2C* | -1.61119 | 0.002708 | *HELB* | -1.64975 | 0.002329 |
| *SNORA61* | -1.60889 | 0.001549 | *SEL1L3* | -1.64875 | 0.000257 |
| *CGNL1* | -1.60367 | 0.019072 | *C12orf45* | -1.64313 | 0.000679 |
| *JUP* | -1.59907 | 0.008482 | *BCL11A* | -1.64216 | 0.000656 |
| *PKP3* | -1.59707 | 0.001636 | *MIR181A1HG* | -1.63573 | 0.009516 |
| *PDGFC* | -1.59639 | 0.002005 | *CST6* | -1.63411 | 0.001767 |
| *LOC647264* | -1.59496 | 0.012243 | *SFN* | -1.63212 | 0.000812 |
| *SNHG12* | -1.59348 | 0.009462 | *SYTL2* | -1.63128 | 0.000185 |
| *CD40* | -1.59152 | 0.015365 | *ACSL1* | -1.62864 | 0.001245 |
| *SLC52A3* | -1.58809 | 0.007359 | *CDCA3* | -1.62482 | 0.000471 |
| *ZSCAN12P1* | -1.58796 | 0.004127 | *BLM* | -1.62236 | 0.003093 |
| *ARHGEF5* | -1.58749 | 0.003498 | *HERC6* | -1.6188 | 0.00133 |
| *KIAA0020* | -1.58591 | 0.009137 | *PIP4K2C* | -1.61649 | 0.000596 |
| *INSIG1* | -1.5851 | 0.007675 | *ARHGEF5* | -1.61639 | 0.003016 |
| *MEST* | -1.5834 | 0.002514 | *NOP16* | -1.61531 | 0.000444 |
| *LY6E* | -1.58337 | 0.005375 | *EREG* | -1.60786 | 0.016169 |
| *RCC1* | -1.58054 | 0.009636 | *GALNT12* | -1.60762 | 0.000243 |
| *HSD11B2* | -1.5794 | 0.004808 | *MMD* | -1.60689 | 0.000963 |
| *CADM1* | -1.57927 | 0.002154 | *REPS2* | -1.60651 | 0.000917 |
| *TTC22* | -1.57905 | 0.002514 | *NAGS* | -1.60486 | 0.000505 |
| *IL17RB* | -1.57877 | 0.029589 | *LRBA* | -1.60148 | 0.000403 |
| *CCDC120* | -1.57748 | 0.003087 | *SERPINB5* | -1.60015 | 0.000874 |
| *AKR1B10* | -1.57383 | 0.042042 | *GOLT1A* | -1.59873 | 0.008876 |
| *EEF1A2* | -1.57276 | 0.009981 | *CYFIP2* | -1.59474 | 0.000295 |
| *ARHGAP18* | -1.57118 | 0.003696 | *INSIG1* | -1.59346 | 0.000992 |
| *GCH1* | -1.57068 | 0.011087 | *SKIL* | -1.58688 | 0.008897 |
| *RPL36* | -1.56614 | 0.018897 | *HMMR* | -1.58408 | 0.000417 |
| *SNORD72* | -1.56587 | 0.033781 | *GATA6* | -1.58392 | 0.004346 |
| *LGALS8* | -1.56564 | 0.0023 | *LMNB1* | -1.58041 | 0.002889 |
| *SDSL* | -1.56433 | 0.012526 | *CCDC120* | -1.58026 | 0.000319 |
| *TMEM54* | -1.56412 | 0.014309 | *EEF1A2* | -1.5775 | 0.00043 |
| *CCDC71L* | -1.56273 | 0.005346 | *FBXO32* | -1.57467 | 0.011361 |
| *SNORA22* | -1.56127 | 0.027724 | *RAB11FIP4* | -1.57344 | 0.002386 |
| *RBMS3* | -1.55559 | 0.011682 | *CALD1* | -1.56948 | 0.000277 |
| *KRT80* | -1.55416 | 0.012866 | *MCOLN3* | -1.56887 | 0.000601 |
| *NFE2L3* | -1.55389 | 0.003572 | *HNRNPAB* | -1.5632 | 0.000568 |
| *HMMR* | -1.54871 | 0.022249 | *MRPL39* | -1.56189 | 0.000775 |
| *HELB* | -1.54786 | 0.006288 | *NMB* | -1.55976 | 0.000508 |
| *KLHL2* | -1.54402 | 0.007689 | *NFE2L3* | -1.55972 | 0.00042 |
| *ARRDC4* | -1.54324 | 0.004697 | *THAP10* | -1.55925 | 0.009474 |
| *LSR* | -1.54241 | 0.007721 | *MDFIC* | -1.55887 | 0.001098 |
| *BID* | -1.54206 | 0.016073 | *RCC1* | -1.55784 | 0.000304 |
| *MDFIC* | -1.5409 | 0.031941 | *LOC101927391* | -1.55591 | 0.00764 |
| *C4orf19* | -1.54084 | 0.011197 | *SDSL* | -1.55525 | 0.001097 |
| *INADL* | -1.54041 | 0.003847 | *SNORA61* | -1.55457 | 0.000729 |
| *SEMA6B* | -1.53486 | 0.004509 | *FUT1* | -1.55313 | 0.003098 |
| *SH3RF2* | -1.53321 | 0.003729 | *WIPF3* | -1.5527 | 0.000822 |
| *MPZL3* | -1.53097 | 0.006981 | *MAP3K1* | -1.55118 | 0.001086 |
| *NAGS* | -1.53001 | 0.006137 | *TUBE1* | -1.55117 | 0.027455 |
| *DLC1* | -1.52983 | 0.002819 | *SSBP1* | -1.54899 | 0.000691 |
| *C10orf12* | -1.52845 | 0.009091 | *MIR4640* | -1.54729 | 0.021397 |
| *PDSS1* | -1.52842 | 0.004572 | *GAL* | -1.5463 | 0.001204 |
| *BCL3* | -1.52826 | 0.011628 | *PDSS1* | -1.5391 | 0.006623 |
| *BCL11A* | -1.52637 | 0.003284 | *BCL3* | -1.5355 | 0.00129 |
| *SCNN1A* | -1.52567 | 0.013153 | *APOBEC3D* | -1.53322 | 0.020449 |
| *SOX4* | -1.52287 | 0.044064 | *FASTKD3* | -1.53169 | 0.013489 |
| *MARS* | -1.51867 | 0.007898 | *NEIL3* | -1.52976 | 0.002408 |
| *NABP1* | -1.51808 | 0.009324 | *TJP2* | -1.52398 | 0.000837 |
| *SNORD93* | -1.51365 | 0.012327 | *C10orf12* | -1.52325 | 0.000466 |
| *PPCDC* | -1.51271 | 0.011373 | *CAMSAP3* | -1.51895 | 0.000568 |
| *WIPF3* | -1.51215 | 0.017164 | *TMEM5* | -1.51682 | 0.001004 |
| *FASTKD3* | -1.50929 | 0.025612 | *NRG4* | -1.50891 | 0.028625 |
| *EGFR* | -1.50893 | 0.00329 | *AKR1B10* | -1.50884 | 0.008468 |
| *SKIL* | -1.50884 | 0.012831 | *KIAA0020* | -1.50834 | 0.001414 |
| *DPYD* | -1.50652 | 0.015672 | *HSD11B2* | -1.50251 | 0.002643 |

**Table S2.** Commonly differentially expressed genes in cabazitaxel-resistant (CZR) versus parental cells from microarray data. Fold change expression of 147 commonly differentially expressed genes in CZR vs parental cell lines in both cell models (DU-145 and PC-3). FC: Fold Change; FDR: False Discovery Rate.

| **COMMON UP-REGULATED GENES in CZR vs. PARENTALS** | | | | | |
| --- | --- | --- | --- | --- | --- |
| **DU-145CZR vs. DU-145** | | | **PC-3CZR vs. PC-3** | | |
| **Gene Symbol** | **FC** | **FDR** | **Gene Symbol** | **FC** | **FDR** |
| *LINC01376* | 3.402513 | 0.015144 | *ITGA1* | 9.41037 | 9.21E-06 |
| *C1S* | 3.081621 | 0.002876 | *SERPINE1* | 8.766929 | 3.53E-06 |
| *CNTNAP1* | 3.032397 | 0.000164 | *MAP1B* | 3.946051 | 0.000273 |
| *ITGA1* | 2.781181 | 0.000388 | *ITGB3* | 3.506101 | 0.002922 |
| *DNAJB9* | 2.593476 | 0.000421 | *STK32A* | 3.417626 | 0.038531 |
| *IL6* | 2.577875 | 0.000654 | *LINC01376* | 3.294901 | 0.004707 |
| *PA2G4P4* | 2.418488 | 0.001364 | *ESM1* | 3.237475 | 0.000287 |
| *SESN3* | 2.416045 | 0.000404 | *KIF5C* | 3.18315 | 0.000858 |
| *TTLL7* | 2.406843 | 0.000293 | *CYBRD1* | 3.137422 | 0.000934 |
| *ALDH1L2* | 2.406488 | 0.000838 | *MMP13* | 2.994118 | 0.004239 |
| *FTX* | 2.399885 | 0.00382 | *RNASE4* | 2.934451 | 0.000934 |
| *GADD45A* | 2.32951 | 0.000345 | *DOCK11* | 2.891485 | 0.001612 |
| *PTPRM* | 2.327321 | 0.000625 | *CACNG7* | 2.860366 | 0.000664 |
| *ERO1LB* | 2.269997 | 0.000478 | *ERN1* | 2.724257 | 0.007843 |
| *ANTXR2* | 2.242064 | 0.000311 | *LAMP3* | 2.714192 | 0.010749 |
| *IL1B* | 2.181707 | 0.003964 | *IFI16* | 2.679257 | 0.00737 |
| *GOLGA8B* | 2.16393 | 0.006301 | *ULBP1* | 2.602367 | 0.006351 |
| *WIPI1* | 2.131898 | 0.000801 | *C1S* | 2.590129 | 0.002117 |
| *ITGA2* | 2.08995 | 0.000461 | *LINC01239* | 2.582202 | 0.037071 |
| *ADAM23* | 2.08341 | 0.001637 | *WIPI1* | 2.465874 | 0.004239 |
| *CD70* | 2.058221 | 0.001842 | *GFPT2* | 2.423612 | 0.002562 |
| *IFI6* | 2.037404 | 0.00076 | *SEPP1* | 2.410888 | 0.003602 |
| *MAP1B* | 2.036666 | 0.000801 | *IL1B* | 2.404571 | 0.002562 |
| *CLIP4* | 2.012994 | 0.000817 | *DYRK3* | 2.344652 | 0.005767 |
| *MMP13* | 2.007054 | 0.000801 | *TTC28* | 2.307062 | 0.003629 |
| *TRPC1* | 1.951024 | 0.001998 | *ZEB1* | 2.298454 | 0.001479 |
| *STK32A* | 1.94956 | 0.008501 | *GOLGA8B* | 2.275372 | 0.005767 |
| *TMEM150C* | 1.946821 | 0.00864 | *DNAJB9* | 2.261363 | 0.042481 |
| *ARNT2* | 1.938893 | 0.003345 | *ERO1LB* | 2.255048 | 0.006184 |
| *CLGN* | 1.93313 | 0.00159 | *IL1RL1* | 2.207038 | 0.008635 |
| *ERN1* | 1.92347 | 0.001773 | *CLIP4* | 2.201408 | 0.006351 |
| *LOC101929378* | 1.915267 | 0.005237 | *CLGN* | 2.131656 | 0.029318 |
| *ITPR2* | 1.910487 | 0.001225 | *FRK* | 2.130557 | 0.02058 |
| *MIR421* | 1.909207 | 0.028503 | *PLD1* | 2.093693 | 0.003442 |
| *SERPINE1* | 1.902611 | 0.006801 | *CREBRF* | 2.076305 | 0.02136 |
| *ESM1* | 1.898454 | 0.026027 | *SYT1* | 2.045882 | 0.026795 |
| *ABCA5* | 1.888233 | 0.00631 | *MIR421* | 2.043497 | 0.056774 |
| *KIF5C* | 1.865093 | 0.005552 | *IL6* | 2.008582 | 0.043906 |
| *PARP3* | 1.847739 | 0.001773 | *IFI6* | 2.000572 | 0.003544 |
| *LINC01239* | 1.846842 | 0.003253 | *CCNG2* | 1.997734 | 0.004313 |
| *TMEM45A* | 1.841579 | 0.002198 | *PPP1R15A* | 1.970692 | 0.022149 |
| *ZBTB20* | 1.839894 | 0.004871 | *ABCA5* | 1.967928 | 0.008635 |
| *RNASE4* | 1.836296 | 0.001814 | *VIMP* | 1.941746 | 0.005621 |
| *ULBP1* | 1.823032 | 0.026438 | *LRRC49* | 1.905599 | 0.022248 |
| *ZEB1* | 1.809308 | 0.001473 | *GRAMD1B* | 1.891544 | 0.002598 |
| *CPEB4* | 1.808107 | 0.015575 | *ITGA2* | 1.866947 | 0.003373 |
| *IL1RL1* | 1.793177 | 0.002404 | *C10orf11* | 1.863892 | 0.009848 |
| *LAMP3* | 1.784342 | 0.006306 | *APLF* | 1.843673 | 0.001913 |
| *LAMB2* | 1.783986 | 0.005747 | *ADAM23* | 1.837145 | 0.010058 |
| *TTC28* | 1.774164 | 0.010996 | *TMEM150C* | 1.83466 | 0.002155 |
| *SYT1* | 1.769486 | 0.003003 | *SESN3* | 1.824014 | 0.001556 |
| *LRRC49* | 1.741597 | 0.005643 | *PTPRM* | 1.822543 | 0.010984 |
| *OPHN1* | 1.73411 | 0.00228 | *ANTXR2* | 1.7948 | 0.007643 |
| *C10orf11* | 1.732734 | 0.009914 | *ULK4P3* | 1.788496 | 0.009923 |
| *TSPAN31* | 1.707059 | 0.004511 | *TRPC1* | 1.780605 | 0.020782 |
| *ZFAND6* | 1.703374 | 0.005513 | *SYCP2* | 1.777583 | 0.023897 |
| *CYBRD1* | 1.696955 | 0.001808 | *RASSF8* | 1.771662 | 0.002363 |
| *ITGB3* | 1.691241 | 0.003287 | *MX1* | 1.73163 | 0.011715 |
| *LOC84214* | 1.68464 | 0.01849 | *FAM174B* | 1.726291 | 0.016823 |
| *ANG* | 1.683602 | 0.010705 | *TMEM158* | 1.723889 | 0.014835 |
| *ISG20* | 1.664333 | 0.002062 | *TMEM45A* | 1.709772 | 0.016376 |
| *APLF* | 1.659643 | 0.005543 | *ALDH1L2* | 1.692902 | 0.008409 |
| *FAM174B* | 1.645194 | 0.016636 | *ZBED6* | 1.690729 | 0.010427 |
| *LINC-PINT* | 1.64107 | 0.010261 | *IFI35* | 1.673983 | 0.002536 |
| *CACNG7* | 1.630922 | 0.007465 | *PLXNA2* | 1.651041 | 0.01366 |
| *VIMP* | 1.625338 | 0.013697 | *ADAMTSL3* | 1.647205 | 0.003902 |
| *MX1* | 1.624202 | 0.003302 | *ISG20* | 1.645323 | 0.029945 |
| *IFI16* | 1.622753 | 0.003972 | *OPHN1* | 1.633775 | 0.034115 |
| *KAT2B* | 1.6186 | 0.002827 | *CREM* | 1.632379 | 0.017101 |
| *GORAB* | 1.617132 | 0.003345 | *APH1B* | 1.631442 | 0.009258 |
| *RASSF8* | 1.61699 | 0.004288 | *LINC00265* | 1.621017 | 0.04531 |
| *SEPP1* | 1.611471 | 0.008145 | *PA2G4P4* | 1.611166 | 0.013227 |
| *TMEM158* | 1.605687 | 0.005289 | *TM7SF2* | 1.607801 | 0.012633 |
| *DOCK11* | 1.59175 | 0.006695 | *ZFAND6* | 1.599278 | 0.010081 |
| *ADAMTSL3* | 1.589105 | 0.012273 | *FGF2* | 1.598294 | 0.024218 |
| *GOLGB1* | 1.583119 | 0.004084 | *LOC84214* | 1.597086 | 0.044022 |
| *IFI35* | 1.578939 | 0.006906 | *CD70* | 1.58664 | 0.035982 |
| *ZBED6* | 1.577226 | 0.002703 | *GOLGB1* | 1.585417 | 0.025737 |
| *PLXNA2* | 1.576909 | 0.005747 | *GADD45A* | 1.582348 | 0.042851 |
| *TM7SF2* | 1.576289 | 0.009605 | *CABYR* | 1.57928 | 0.012351 |
| *GFPT2* | 1.575707 | 0.004084 | *TSPAN31* | 1.552319 | 0.022892 |
| *ULK4P3* | 1.573987 | 0.028919 | *LAMB2* | 1.551512 | 0.02577 |
| *PPP1R15A* | 1.572555 | 0.005663 | *PHF21A* | 1.549279 | 0.023377 |
| *ANKRD12* | 1.569892 | 0.00487 | *FRMD4B* | 1.546793 | 0.012046 |
| *PLD1* | 1.559363 | 0.012723 | *SNORD98* | 1.545956 | 0.032116 |
| *PHF21A* | 1.553589 | 0.003064 | *ZBTB20* | 1.542978 | 0.02512 |
| *FRK* | 1.552043 | 0.017434 | *KAT2B* | 1.542076 | 0.018583 |
| *INPPL1* | 1.551074 | 0.009067 | *FTX* | 1.541987 | 0.031897 |
| *CABYR* | 1.549358 | 0.004181 | *LOC101929378* | 1.540323 | 0.014806 |
| *CREM* | 1.548158 | 0.002522 | *ITPR2* | 1.539404 | 0.007143 |
| *APH1B* | 1.547809 | 0.006911 | *LINC-PINT* | 1.535444 | 0.020084 |
| *FUOM* | 1.54727 | 0.008014 | *TTLL7* | 1.535381 | 0.01006 |
| *DYRK3* | 1.54531 | 0.00506 | *ANKRD12* | 1.533643 | 0.018099 |
| *FGF2* | 1.536142 | 0.002703 | *CPEB4* | 1.532897 | 0.015049 |
| *CREBRF* | 1.522496 | 0.024871 | *PARP3* | 1.530663 | 0.009323 |
| *SNORD98* | 1.520894 | 0.010498 | *CNTNAP1* | 1.522022 | 0.024474 |
| *CCNG2* | 1.515071 | 0.021437 | *ANG* | 1.520041 | 0.041336 |
| *LINC00265* | 1.510681 | 0.01284 | *GORAB* | 1.512116 | 0.029301 |
| *FRMD4B* | 1.51048 | 0.049706 | *ARNT2* | 1.511675 | 0.036272 |
| *GRAMD1B* | 1.510024 | 0.008653 | *FUOM* | 1.506604 | 0.008233 |
| *SYCP2* | 1.505344 | 0.006553 | *INPPL1* | 1.505048 | 0.012637 |
| **COMMON DOWN-REGULATED GENES in CZR vs. PARENTALS** | | | | | |
| **DU-145CZR vs. DU-145** | | | **PC-3CZR vs. PC-3** | | |
| **Gene Symbol** | **FC** | **FDR** | **Gene Symbol** | **FC** | **FDR** |
| *CST6* | -3.6889 | 0.000554 | HIST1H3B | -3.68269 | 0.005314 |
| *CDK6* | -2.47044 | 0.000676 | CDCA7 | -3.12251 | 0.023142 |
| *HIST1H2AK* | -2.44061 | 0.001983 | ORC1 | -2.76727 | 0.022167 |
| *AIF1L* | -2.22496 | 0.008198 | HIST1H2AM | -2.34603 | 0.023182 |
| *ZNF100* | -2.0425 | 0.028042 | HIST1H3F | -2.27756 | 0.020717 |
| *CENPH* | -2.01606 | 0.000637 | LMNB1 | -2.2507 | 0.01366 |
| *F3* | -1.97335 | 0.00159 | HIST1H4A | -2.23527 | 0.028354 |
| *HIST1H4A* | -1.97073 | 0.011624 | TLE4 | -2.19457 | 0.004014 |
| *CPM* | -1.93322 | 0.000923 | MCM3 | -2.17441 | 0.020227 |
| *SNORD91B* | -1.90837 | 0.001773 | ZNF100 | -2.12441 | 0.001331 |
| *HIST1H3B* | -1.89563 | 0.003434 | GMNN | -2.02601 | 0.013713 |
| *LPCAT1* | -1.89541 | 0.000862 | HIST1H2AK | -2.01651 | 0.028599 |
| *HIST1H2AI* | -1.89083 | 0.002123 | HIST1H2AI | -1.99572 | 0.035693 |
| *HIST1H3H* | -1.8577 | 0.002483 | MCM4 | -1.98956 | 0.034957 |
| *HIST1H2AM* | -1.84233 | 0.001209 | RAD54L | -1.93891 | 0.045151 |
| *BMP4* | -1.83703 | 0.001532 | LPCAT1 | -1.90988 | 0.010081 |
| *HNRNPA1P33* | -1.79328 | 0.007525 | DSCC1 | -1.90922 | 0.01924 |
| *SNORD49B* | -1.79217 | 0.002374 | BMP4 | -1.90086 | 0.008199 |
| *GMNN* | -1.78392 | 0.002323 | CDK6 | -1.89633 | 0.000934 |
| *RPP40* | -1.70525 | 0.009061 | SNORD3D | -1.89533 | 0.011714 |
| *CDCA7* | -1.70176 | 0.00167 | CAV1 | -1.87726 | 0.004937 |
| *AMOT* | -1.67336 | 0.00164 | SNORD49B | -1.82192 | 0.006351 |
| *BRIX1* | -1.67201 | 0.002768 | SHMT1 | -1.8012 | 0.016858 |
| *GEMIN4* | -1.65405 | 0.002495 | SNORD24 | -1.75139 | 0.001891 |
| *MYBL2* | -1.65076 | 0.001993 | FAM83D | -1.7261 | 0.027243 |
| *TMPO* | -1.63327 | 0.002374 | CST6 | -1.72366 | 0.009029 |
| *RIBC2* | -1.6178 | 0.012583 | RECQL4 | -1.70543 | 0.046447 |
| *HIST1H3F* | -1.6077 | 0.047351 | SLC29A1 | -1.69809 | 0.049142 |
| *CAV1* | -1.60718 | 0.003326 | HNRNPA1P33 | -1.65834 | 0.008233 |
| *CAV2* | -1.60504 | 0.004888 | TMPO | -1.64922 | 0.023893 |
| *SHMT1* | -1.60464 | 0.015112 | GEMIN4 | -1.64299 | 0.00995 |
| *SLC29A1* | -1.59811 | 0.003659 | AIF1L | -1.62684 | 0.022964 |
| *SOX4* | -1.59479 | 0.043468 | AMOT | -1.61722 | 0.008871 |
| *MCM3* | -1.56686 | 0.002404 | RIBC2 | -1.60436 | 0.028787 |
| *DSCC1* | -1.56368 | 0.012135 | SNORD91B | -1.60196 | 0.017316 |
| *SNORD3D* | -1.55574 | 0.043408 | CAV2 | -1.5954 | 0.007182 |
| *LMNB1* | -1.54755 | 0.002681 | MYBL2 | -1.56249 | 0.046395 |
| *TLE4* | -1.54662 | 0.019047 | SOX4 | -1.56093 | 0.03453 |
| *RECQL4* | -1.54397 | 0.017285 | F3 | -1.55844 | 0.010026 |
| *ELAC2* | -1.53893 | 0.003239 | CENPH | -1.54793 | 0.018359 |
| *SNORD24* | -1.53696 | 0.013764 | RPP40 | -1.54477 | 0.013108 |
| *FAM83D* | -1.53273 | 0.002829 | ELAC2 | -1.53912 | 0.0185 |
| *MCM4* | -1.51842 | 0.003031 | CPM | -1.53232 | 0.009029 |
| *THEM6* | -1.51535 | 0.021583 | HIST1H3H | -1.51597 | 0.020782 |
| *RAD54L* | -1.51284 | 0.00718 | THEM6 | -1.51181 | 0.0427 |
| *ORC1* | -1.51199 | 0.005747 | BRIX1 | -1.511 | 0.014842 |

**Table S3.** Commonly differentially expressed genes in taxane-resistant versus parental cells from microarray data. Fold change expression of 23 commonly differentially expressed genes in resistant cells vs parental cells in both resistant (DR and CZR) and parental cell models (DU-145 and PC-3). FC: Fold Change; FDR: False Discovery Rate; DR: Docetaxel-resistant; CZ: Cabazitaxel-resistant.

| **COMMON UP-REGULATED GENES in R vs. Parentals** | | | | | | | | |
| --- | --- | --- | --- | --- | --- | --- | --- | --- |
| **Gene symbol** | **DU-145DR** | | **DU-145CZR** | | **PC-3DR** | | **PC-3CZR** | |
|  | **FC** | **FDR** | **FC** | **FDR** | **FC** | **FDR** | **FC** | **FDR** |
| *ZBED6* | 1.798452 | 0.001229 | 1.577226 | 0.002703 | 2.287252 | 3.96E-05 | 1.690729 | 0.010427 |
| *ANTXR2* | 2.273965 | 9.13E-05 | 2.242064 | 0.000311 | 2.626603 | 8.36E-06 | 1.7948 | 0.007643 |
| *TTC28* | 2.482349 | 0.000352 | 1.774164 | 0.010996 | 4.036168 | 3.48E-06 | 2.307062 | 0.003629 |
| *IFI6* | 1.539199 | 0.006736 | 2.037404 | 0.00076 | 3.994021 | 3.69E-06 | 2.000572 | 0.003544 |
| *INPPL1* | 2.271854 | 0.000263 | 1.551074 | 0.009067 | 1.70407 | 0.000254 | 1.505048 | 0.012637 |
| *ITGA1* | 1.948904 | 0.00408 | 2.781181 | 0.000388 | 8.102511 | 2.76E-07 | 9.41037 | 9.21E-06 |
| *ITGB3* | 6.734176 | 5.5E-07 | 1.691241 | 0.003287 | 6.891471 | 4.49E-07 | 3.506101 | 0.002922 |
| *PLD1* | 1.877562 | 0.000361 | 1.559363 | 0.012723 | 3.294461 | 8.69E-07 | 2.093693 | 0.003442 |
| *WIPI1* | 2.009064 | 0.000365 | 2.131898 | 0.000801 | 1.987061 | 3.13E-05 | 2.465874 | 0.004239 |
| *TMEM45A* | 3.137568 | 1.28E-05 | 1.841579 | 0.002198 | 1.668914 | 0.001036 | 1.709772 | 0.016376 |
| *PTPRM* | 4.2902 | 2.18E-06 | 2.327321 | 0.000625 | 2.851846 | 2.04E-06 | 1.822543 | 0.010984 |
| *ZEB1* | 1.937094 | 0.001183 | 1.809308 | 0.001473 | 8.471464 | 3.11E-07 | 2.298454 | 0.001479 |
| *TM7SF2* | 1.641597 | 0.003156 | 1.576289 | 0.009605 | 1.610128 | 0.001086 | 1.607801 | 0.012633 |
| *TRPC1* | 2.333377 | 0.000426 | 1.951024 | 0.001998 | 1.781472 | 0.002809 | 1.780605 | 0.020782 |
| *CYBRD1* | 2.778985 | 4.92E-05 | 1.696955 | 0.001808 | 8.582694 | 4.68E-07 | 3.137422 | 0.000934 |
| *CNTNAP1* | 2.851755 | 3.95E-05 | 3.032397 | 0.000164 | 2.762463 | 9.7E-06 | 1.522022 | 0.024474 |
| *ADAM23* | 1.768979 | 0.001994 | 2.08341 | 0.001637 | 2.423116 | 9.63E-05 | 1.837145 | 0.010058 |
| *CD70* | 1.667208 | 0.004077 | 2.058221 | 0.001842 | 8.284467 | 4.87E-07 | 1.58664 | 0.035982 |
| **COMMON DOWN-REGULATED GENES in R vs. Parentals** | | | | | | | | |
| **Gene symbol** | **DU-145DR** | | **DU-145CZR** | | **PC-3DR** | | **PC-3CZR** | |
|  | **FC** | **FDR** | **FC** | **FDR** | **FC** | **FDR** | **FC** | **FDR** |
| *CST6* | -4.61972 | 3.89E-06 | -3.6889 | 0.000554 | -1.63411 | 0.001767 | -1.72366 | 0.009029 |
| *RIBC2* | -1.66135 | 0.003559 | -1.6178 | 0.012583 | -2.08296 | 0.000324 | -1.60436 | 0.028787 |
| *LMNB1* | -1.67786 | 0.001182 | -1.54755 | 0.002681 | -1.58041 | 0.002889 | -2.2507 | 0.01366 |
| *BMP4* | -6.17783 | 1.69E-06 | -1.83703 | 0.001532 | -6.09643 | 4.66E-07 | -1.90086 | 0.008199 |
| *SOX4* | -1.52287 | 0.044064 | -1.59479 | 0.043468 | -2.06638 | 2.79E-05 | -1.56093 | 0.03453 |

**Table S4.** Commonly differentially expressed genes in cabazitaxel-resistant (CZR) versus docetaxel-resistant (DR) cells from microarray data. Fold change expression of 559 commonly differentially expressed genes in CZR vs DR cell lines in both cell models (DU-145 and PC-3). FC: Fold Change; FDR: False Discovery Rate.

| **COMMON UP-REGULATED GENES in CZR vs. DR** | | | | | |
| --- | --- | --- | --- | --- | --- |
| **DU-145CZR vs. DU-145DR** | | | **PC-3CZR vs. PC-3DR** | | |
| **Gene Symbol** | **FC** | **FDR** | **Gene Symbol** | **FC** | **FDR** |
| *MAL2* | 89.24227 | 6.22E-09 | *ESRP1* | 117.6798 | 6.96E-09 |
| *TC2N* | 27.59321 | 7.34E-07 | *TC2N* | 43.57968 | 6.96E-09 |
| *KRTAP2-3* | 25.77504 | 5.87E-08 | *CDH1* | 42.1148 | 6.96E-09 |
| *MPZL2* | 17.70197 | 1.93E-07 | *MPZL2* | 39.51691 | 3.41E-08 |
| *MME* | 16.1646 | 2.08E-07 | *OCLN* | 39.51635 | 4.91E-07 |
| *LAMC2* | 15.46964 | 5.87E-08 | *EFEMP1* | 33.49979 | 6.96E-09 |
| *TACSTD2* | 15.14354 | 2.35E-06 | *GJB2* | 24.35794 | 1.73E-05 |
| *TRIM2* | 14.91635 | 8.84E-07 | *PLEKHG1* | 21.7533 | 4.66E-07 |
| *GPR87* | 13.69786 | 2.89E-07 | *TMEM45B* | 21.37771 | 4.66E-07 |
| *SERPINA1* | 12.78669 | 2.81E-06 | *EPCAM* | 18.8743 | 1.05E-08 |
| *SEMA3A* | 11.63339 | 2.6E-06 | *SLC1A3* | 17.91141 | 1.69E-07 |
| *GBP1* | 10.76857 | 2.3E-06 | *ST14* | 17.72339 | 2.52E-07 |
| *LOC100996579* | 9.271524 | 1.56E-06 | *FAM84B* | 17.12682 | 1.67E-08 |
| *C1orf116* | 9.066637 | 4.37E-07 | *DDIT4* | 14.92446 | 2.58E-08 |
| *GALNT3* | 8.814384 | 1.14E-06 | *TACSTD2* | 14.70803 | 1.52E-07 |
| *TMEM45B* | 8.021527 | 8.88E-07 | *LCN2* | 13.84876 | 3.34E-07 |
| *CD274* | 7.629813 | 3.52E-07 | *PTGFRN* | 13.17936 | 2.03E-07 |
| *CALD1* | 7.538283 | 5.24E-06 | *SLC27A2* | 12.95229 | 4.12E-06 |
| *SYTL2* | 7.399592 | 3.47E-06 | *TMEM30B* | 12.89563 | 3.59E-08 |
| *GOLT1A* | 7.242675 | 1.82E-06 | *TXNIP* | 12.51429 | 1.07E-06 |
| *VWDE* | 6.740058 | 4.39E-07 | *NNMT* | 11.1968 | 3.68E-06 |
| *IL7* | 6.565753 | 4.65E-05 | *SERPINA1* | 10.19806 | 6.21E-07 |
| *TMEM156* | 6.52326 | 7.8E-07 | *HOXB9* | 10.16124 | 4.59E-06 |
| *VAMP8* | 6.409754 | 1.14E-06 | *MYZAP* | 9.491654 | 3.63E-07 |
| *KAL1* | 6.174684 | 8.57E-07 | *LPCAT2* | 9.221253 | 5.67E-07 |
| *CSF2RA* | 6.025789 | 3.07E-05 | *TNS4* | 8.779876 | 9.1E-06 |
| *EPCAM* | 6.024264 | 5.19E-07 | *CDS1* | 8.572589 | 6.61E-08 |
| *SPINT1* | 5.833943 | 1.94E-06 | *LOC647859* | 8.382357 | 1.44E-06 |
| *CDH1* | 5.787778 | 2.6E-06 | *C2orf15* | 8.318506 | 1.35E-06 |
| *PLA2G4A* | 5.775607 | 1.94E-06 | *DPYD* | 8.165968 | 8.91E-08 |
| *CASP4* | 5.726081 | 8.93E-07 | *LAD1* | 8.074635 | 6.85E-07 |
| *AFAP1-AS1* | 5.718883 | 2.62E-06 | *GALNT3* | 7.274704 | 2.03E-07 |
| *ST14* | 5.595976 | 9.39E-06 | *ARAP2* | 7.169619 | 3.6E-07 |
| *EREG* | 5.503507 | 1.16E-06 | *SLC6A11* | 7.09478 | 3.1E-06 |
| *SYK* | 5.168414 | 8.58E-07 | *STK32A* | 6.678179 | 0.001469 |
| *ATP10A* | 5.082353 | 5.85E-06 | *IRF6* | 6.556566 | 1.33E-07 |
| *SCN9A* | 4.769531 | 8.6E-06 | *DLC1* | 6.356806 | 4.66E-07 |
| *SLFN5* | 4.72045 | 4.1E-06 | *IFI16* | 6.117526 | 2.74E-05 |
| *DDX60* | 4.58939 | 1.94E-06 | *SPINT1* | 6.030265 | 2.3E-07 |
| *LPXN* | 4.474813 | 2.59E-06 | *CLDN1* | 5.979934 | 5.7E-07 |
| *FBXO32* | 4.470939 | 8.6E-06 | *MPZL3* | 5.979596 | 4.31E-06 |
| *ELOVL7* | 4.460227 | 2.51E-05 | *MAP7* | 5.776823 | 1.74E-07 |
| *TSPAN31* | 4.444643 | 2.86E-06 | *FAM174B* | 5.733952 | 7.1E-06 |
| *DPYD* | 4.441731 | 4.04E-06 | *SH3GL2* | 5.705586 | 1.75E-06 |
| *RNF43* | 4.393237 | 7.03E-06 | *CHMP4C* | 5.584122 | 6.85E-07 |
| *FRMD4B* | 4.336934 | 8.94E-05 | *SMPDL3B* | 5.368837 | 3.19E-07 |
| *PRRG4* | 4.335255 | 7.03E-06 | *PRRG4* | 5.347418 | 1.5E-06 |
| *LAMP3* | 4.247277 | 3.97E-06 | *PRR15* | 5.339411 | 1.09E-05 |
| *APOBEC3D* | 4.203721 | 0.000143 | *SEPP1* | 5.326473 | 4.93E-06 |
| *DDIT4* | 4.184458 | 4.72E-06 | *MARVELD2* | 5.275294 | 3.62E-06 |
| *SMPDL3B* | 4.124079 | 1.12E-05 | *MOCOS* | 5.097689 | 4.59E-06 |
| *IFIH1* | 4.070173 | 7.7E-06 | *LSR* | 5.040675 | 2.52E-07 |
| *GDAP1* | 4.05978 | 2.08E-05 | *ZNF165* | 5.01444 | 3.34E-07 |
| *KIAA1244* | 4.054498 | 9.98E-06 | *MARVELD3* | 4.994898 | 1.39E-06 |
| *TRIM38* | 4.024584 | 6.09E-06 | *ATP10A* | 4.889673 | 6.82E-06 |
| *PBX1* | 4.017801 | 0.000334 | *MICAL2* | 4.869132 | 2.4E-05 |
| *ESRP1* | 4.008397 | 6.75E-06 | *HOOK1* | 4.859332 | 2.61E-07 |
| *IRF1* | 3.994053 | 4.91E-06 | *C1orf116* | 4.812617 | 1.97E-06 |
| *JPH1* | 3.926703 | 8.64E-05 | *MBNL3* | 4.784069 | 3.63E-07 |
| *RAB17* | 3.881297 | 0.000127 | *OR7E91P* | 4.761214 | 2.85E-06 |
| *IFI16* | 3.820175 | 2.34E-05 | *RPS6KA2* | 4.728221 | 2.55E-05 |
| *CLDN1* | 3.681344 | 3.21E-06 | *ATF3* | 4.707345 | 0.000116 |
| *TXNIP* | 3.658053 | 7.53E-05 | *EFNB2* | 4.658571 | 3.26E-07 |
| *C1S* | 3.638006 | 9.36E-05 | *MAP2* | 4.64077 | 7.56E-05 |
| *HERPUD1* | 3.594644 | 3.49E-06 | *ABHD17C* | 4.354799 | 1.1E-05 |
| *ARNT2* | 3.58779 | 0.000112 | *SLC37A1* | 4.29506 | 1.45E-05 |
| *MTUS1* | 3.558896 | 6.88E-05 | *CADPS2* | 4.28942 | 1.17E-05 |
| *FAM84B* | 3.511401 | 4.38E-05 | *CDC42EP3* | 4.182769 | 7.42E-06 |
| *NMI* | 3.463327 | 4.91E-06 | *WSCD1* | 4.094985 | 2.08E-06 |
| *ERAP1* | 3.462382 | 4.1E-06 | *CD274* | 4.093043 | 1.39E-06 |
| *TAOK3* | 3.422513 | 4.09E-06 | *MYO6* | 4.088295 | 1.94E-06 |
| *HOXB9* | 3.413962 | 4.91E-06 | *MAPK13* | 4.035755 | 1.01E-06 |
| *DMKN* | 3.393505 | 1.12E-05 | *TRIB3* | 3.966131 | 4.59E-06 |
| *APOL6* | 3.3916 | 6.32E-05 | *IFIH1* | 3.94348 | 1.17E-05 |
| *FLJ23867* | 3.364239 | 9.39E-05 | *JPH1* | 3.919674 | 8.66E-05 |
| *BMP4* | 3.362952 | 2.81E-05 | *SH2D3A* | 3.905522 | 1.42E-05 |
| *SFMBT2* | 3.303151 | 9.26E-06 | *SELL* | 3.891197 | 0.000137 |
| *GJB2* | 3.284427 | 2.32E-05 | *SCN9A* | 3.875892 | 4.69E-05 |
| *ASNS* | 3.277446 | 4.54E-06 | *INADL* | 3.873061 | 1.61E-06 |
| *PLEKHA5* | 3.242574 | 4.99E-06 | *CD40* | 3.852553 | 1.04E-05 |
| *STYK1* | 3.239071 | 3.23E-05 | *HOXB2* | 3.835083 | 2.72E-05 |
| *FAM111A* | 3.181875 | 9.7E-06 | *ARRDC4* | 3.800594 | 6.52E-06 |
| *STEAP1* | 3.165367 | 0.022473 | *SESN2* | 3.783227 | 1.68E-05 |
| *DLG3* | 3.122111 | 0.001267 | *C4orf32* | 3.738077 | 8.19E-06 |
| *WSCD1* | 3.119397 | 1.72E-05 | *MOXD1* | 3.737193 | 1.07E-05 |
| *ABHD17C* | 3.114301 | 6.17E-05 | *ASNS* | 3.727503 | 1.69E-05 |
| *CDS1* | 3.101099 | 1.78E-05 | *SYK* | 3.60427 | 1.27E-05 |
| *CHAC1* | 3.093367 | 4.85E-05 | *SH3BGRL2* | 3.557605 | 3.46E-05 |
| *TJP2* | 3.070355 | 1.37E-05 | *ZDHHC21* | 3.55511 | 2.1E-06 |
| *ULBP1* | 3.055516 | 0.000195 | *SNHG8* | 3.552879 | 1.1E-05 |
| *PARP12* | 3.008474 | 3.18E-05 | *SLFN5* | 3.491938 | 0.000202 |
| *FAM160A1* | 3.000587 | 2.32E-05 | *TINAGL1* | 3.490114 | 6.96E-06 |
| *RARRES3* | 2.974692 | 2.81E-05 | *FAM46C* | 3.30511 | 7.55E-06 |
| *MCTP2* | 2.957741 | 0.000111 | *RNF43* | 3.290711 | 4.51E-05 |
| *ANK3* | 2.94351 | 0.000109 | *ICA1* | 3.261573 | 0.000322 |
| *HOXB2* | 2.925094 | 0.000322 | *BAMBI* | 3.232205 | 4.69E-06 |
| *GALNT12* | 2.863321 | 0.000156 | *BMP4* | 3.207196 | 6.81E-05 |
| *SEPP1* | 2.838924 | 4.8E-05 | *C10orf32* | 3.148061 | 0.000343 |
| *PPP1R14C* | 2.800401 | 0.000556 | *STEAP4* | 3.146986 | 5.25E-06 |
| *FAM157A* | 2.776369 | 0.000394 | *GUCY1B3* | 3.113557 | 4.38E-06 |
| *CADPS2* | 2.732999 | 4.14E-05 | *IFIT2* | 3.097169 | 5.76E-05 |
| *EGLN3* | 2.732799 | 5.15E-05 | *ERN1* | 3.089519 | 7.42E-06 |
| *MYZAP* | 2.695842 | 0.007397 | *TAOK3* | 3.049917 | 3.5E-06 |
| *SLC41A2* | 2.671041 | 0.000123 | *RIPK4* | 3.017448 | 7.17E-06 |
| *MIR181A1HG* | 2.659621 | 0.000219 | *C17orf51* | 3.015831 | 4.59E-06 |
| *RPS6KA6* | 2.659543 | 0.000168 | *GPR87* | 3.014342 | 4.59E-06 |
| *SH3RF1* | 2.659409 | 6.32E-05 | *LOC101927204* | 3.012207 | 1.77E-05 |
| *LINC00702* | 2.658672 | 8.65E-05 | *PLA2G4A* | 3.006608 | 6.1E-05 |
| *FAM169A* | 2.647296 | 9.78E-05 | *SEMA3E* | 2.999978 | 8.1E-06 |
| *SEL1L3* | 2.64632 | 3.09E-05 | *OASL* | 2.991566 | 4.79E-06 |
| *FAM83A* | 2.64547 | 2.39E-05 | *ULBP1* | 2.947544 | 2.4E-06 |
| *ROR1* | 2.643804 | 2.66E-05 | *ELOVL7* | 2.946007 | 4.12E-05 |
| *B4GALNT3* | 2.614414 | 3.21E-05 | *ROR1* | 2.925109 | 6.19E-06 |
| *MBNL3* | 2.612176 | 3.26E-05 | *GLS2* | 2.902196 | 6.33E-05 |
| *RIPK4* | 2.605451 | 0.00035 | *LINC01239* | 2.867837 | 0.009553 |
| *MOB3B* | 2.605062 | 3.06E-05 | *KAL1* | 2.853613 | 0.000306 |
| *MANSC1* | 2.602971 | 0.000128 | *VDR* | 2.827823 | 2.8E-06 |
| *LIPG* | 2.60099 | 4.86E-05 | *ARHGAP28* | 2.820683 | 6.75E-05 |
| *PLEKHG1* | 2.592475 | 0.000147 | *ZBED3* | 2.810801 | 0.000219 |
| *LOC101927391* | 2.587342 | 0.031996 | *MNS1* | 2.809377 | 7.56E-05 |
| *LAD1* | 2.574742 | 0.000691 | *ESRP2* | 2.806458 | 4.19E-06 |
| *HES1* | 2.567338 | 0.000112 | *LOC101927391* | 2.799146 | 0.000113 |
| *SLC12A8* | 2.558277 | 0.000158 | *PADI2* | 2.792442 | 0.000267 |
| *SLC37A1* | 2.557134 | 5.5E-05 | *RAB19* | 2.792247 | 2.74E-05 |
| *ARHGAP10* | 2.547502 | 4.18E-05 | *ALOX12P2* | 2.745818 | 1.3E-05 |
| *UPP1* | 2.544723 | 0.000127 | *WWC1* | 2.745057 | 4.82E-06 |
| *VDR* | 2.53928 | 4.85E-05 | *FAM83A* | 2.738404 | 2.52E-05 |
| *EFEMP1* | 2.532426 | 2.2E-05 | *FGD4* | 2.718249 | 3.05E-05 |
| *SLC6A11* | 2.512837 | 8.64E-05 | *EREG* | 2.717627 | 1.07E-05 |
| *ARHGAP28* | 2.505246 | 0.000383 | *RTKN2* | 2.663004 | 2.63E-05 |
| *IRF6* | 2.497829 | 6.17E-05 | *MIR622* | 2.634012 | 7.42E-06 |
| *HDAC9* | 2.493843 | 7.64E-05 | *AIG1* | 2.612323 | 6.19E-06 |
| *MYO6* | 2.491312 | 0.00033 | *STAP2* | 2.599191 | 1.01E-05 |
| *LACC1* | 2.483951 | 0.000467 | *ARHGAP44* | 2.579973 | 1.16E-05 |
| *LRRC49* | 2.452765 | 0.000314 | *RARRES3* | 2.565138 | 0.001523 |
| *IDS* | 2.44529 | 3.63E-05 | *LAT2* | 2.562294 | 0.000103 |
| *PTGFRN* | 2.444552 | 7.97E-05 | *DNAJC1* | 2.550182 | 3.45E-05 |
| *SLC27A2* | 2.44425 | 9.4E-05 | *LGALS8* | 2.545077 | 8.73E-05 |
| *TMEM87B* | 2.43694 | 0.000142 | *PBX1* | 2.535206 | 2.99E-05 |
| *CXCL2* | 2.428699 | 0.000192 | *ALDH1A3* | 2.533779 | 1.05E-05 |
| *LINC00673* | 2.424426 | 5.29E-05 | *SAMD12* | 2.532144 | 1.93E-05 |
| *SNX24* | 2.419793 | 0.000173 | *PTER* | 2.530011 | 0.000174 |
| *SAMD12* | 2.40794 | 0.000645 | *TRIM2* | 2.529016 | 4.96E-05 |
| *BIRC3* | 2.398838 | 0.00132 | *IL17RE* | 2.522357 | 0.000148 |
| *SELL* | 2.397201 | 0.000825 | *MYO5B* | 2.514583 | 0.000109 |
| *RAB19* | 2.388424 | 0.000383 | *MANSC1* | 2.513379 | 1.71E-05 |
| *SNHG12* | 2.381172 | 4.82E-05 | *LINC01116* | 2.492868 | 3.07E-05 |
| *ZNF585B* | 2.37809 | 0.001389 | *CSF2RA* | 2.477417 | 0.000156 |
| *OCLN* | 2.371725 | 0.000125 | *PLEKHB1* | 2.475099 | 1.17E-05 |
| *MAP2* | 2.344027 | 0.000121 | *NIPAL1* | 2.474803 | 8.11E-06 |
| *ELL2* | 2.329234 | 4.78E-05 | *KLHL2* | 2.469522 | 8.09E-05 |
| *CBLC* | 2.326372 | 0.000917 | *SDC4* | 2.464354 | 2.64E-05 |
| *MOCOS* | 2.306686 | 0.00012 | *LRIG1* | 2.463871 | 0.000188 |
| *MAP7* | 2.306151 | 0.000136 | *SNX10* | 2.463366 | 0.00082 |
| *SPX* | 2.294115 | 0.000234 | *FAAH2* | 2.463319 | 6.28E-05 |
| *PPP1R1C* | 2.293361 | 0.000116 | *MLLT3* | 2.447685 | 1.67E-05 |
| *GRPEL2* | 2.265065 | 0.000197 | *PDE9A* | 2.434539 | 0.000138 |
| *FGD4* | 2.263979 | 0.000156 | *LAMP3* | 2.424456 | 0.004202 |
| *SLC1A3* | 2.250553 | 0.000291 | *VWDE* | 2.422568 | 8.8E-06 |
| *FAM174B* | 2.219091 | 0.000101 | *ELF3* | 2.421973 | 0.000335 |
| *MAPK13* | 2.205133 | 0.000161 | *DRAM1* | 2.414938 | 3.81E-05 |
| *AVEN* | 2.197292 | 0.000123 | *VAMP8* | 2.408816 | 1.29E-05 |
| *ALDH1A3* | 2.190438 | 0.000235 | *STYK1* | 2.396539 | 0.000173 |
| *ANKFN1* | 2.170828 | 0.000175 | *ZSCAN12P1* | 2.393461 | 4.29E-05 |
| *PYROXD1* | 2.169362 | 0.009978 | *NIPAL2* | 2.391132 | 0.000132 |
| *DNAJC6* | 2.167656 | 0.000195 | *LRRC49* | 2.359747 | 0.001081 |
| *BAZ2B* | 2.166463 | 0.001137 | *TUBE1* | 2.355517 | 2.42E-05 |
| *BEST1* | 2.159484 | 0.00014 | *RAB17* | 2.352804 | 6.92E-05 |
| *DLC1* | 2.158746 | 8.15E-05 | *FBXO32* | 2.325283 | 0.000567 |
| *APLF* | 2.153332 | 0.000193 | *RBMS3* | 2.313226 | 0.000141 |
| *LCN2* | 2.151132 | 0.007268 | *KIAA0922* | 2.312621 | 0.000111 |
| *LOC101927204* | 2.145983 | 0.002608 | *KIAA1244* | 2.309503 | 0.000112 |
| *NAGS* | 2.145043 | 0.000165 | *BIRC3* | 2.285321 | 4.2E-05 |
| *RPS6KA2* | 2.138822 | 0.00164 | *SKIL* | 2.272248 | 0.000115 |
| *RBMS3* | 2.132274 | 0.000525 | *MCTP2* | 2.25145 | 5.95E-05 |
| *SLIT2* | 2.127642 | 0.002955 | *FA2H* | 2.250959 | 0.000166 |
| *SMIM6* | 2.125976 | 0.000677 | *FAM66C* | 2.232163 | 3.96E-05 |
| *GUCY1B3* | 2.117528 | 0.000108 | *C1S* | 2.228546 | 0.00099 |
| *CAMK1D* | 2.113178 | 0.000116 | *TMEM156* | 2.220207 | 8.9E-05 |
| *CLMP* | 2.103206 | 0.001353 | *ANK3* | 2.213162 | 1.37E-05 |
| *SDCCAG8* | 2.102189 | 0.000248 | *OGFRL1* | 2.210205 | 3.15E-05 |
| *NABP1* | 2.088856 | 0.000575 | *DERA* | 2.196424 | 5.47E-05 |
| *DCP1B* | 2.087929 | 0.000192 | *SFMBT2* | 2.196165 | 0.001056 |
| *CCDC82* | 2.078625 | 0.005336 | *CFH* | 2.192403 | 0.001145 |
| *KLHL2* | 2.073027 | 0.000492 | *FAS* | 2.187078 | 3.54E-05 |
| *MRPL35* | 2.066244 | 0.000482 | *FLJ23867* | 2.185966 | 0.045438 |
| *SNX10* | 2.062139 | 0.000472 | *GTPBP10* | 2.183365 | 2.14E-05 |
| *FAM110C* | 2.05771 | 0.000503 | *ZNF774* | 2.175663 | 0.000237 |
| *PA2G4P4* | 2.045992 | 0.006817 | *SNORA33* | 2.169269 | 0.003137 |
| *AIG1* | 2.037001 | 0.000214 | *SLC12A8* | 2.157272 | 3.81E-05 |
| *CGNL1* | 2.035108 | 0.001065 | *ARFGAP2* | 2.150068 | 4.58E-05 |
| *LRBA* | 2.02267 | 0.000164 | *FRMD5* | 2.147803 | 8.35E-05 |
| *SH3BGRL2* | 2.022224 | 0.00065 | *LOC374443* | 2.142185 | 0.000115 |
| *HERC6* | 2.017435 | 0.000763 | *MRPL15* | 2.123583 | 0.000371 |
| *ERN1* | 1.999553 | 0.000464 | *APLF* | 2.120314 | 9.14E-05 |
| *KIAA1147* | 1.992894 | 0.000143 | *SEL1L3* | 2.119134 | 5.8E-05 |
| *MMD* | 1.991883 | 0.000265 | *ACTR3C* | 2.115917 | 0.00024 |
| *IRAK2* | 1.991533 | 0.000214 | *FAM111A* | 2.098127 | 0.000221 |
| *LOC374443* | 1.989775 | 0.000458 | *HOXB3* | 2.093973 | 0.000164 |
| *STK32A* | 1.986983 | 0.000548 | *FAM157A* | 2.093965 | 0.015949 |
| *NIPAL1* | 1.985026 | 0.000513 | *LOC647264* | 2.07949 | 0.000521 |
| *CCDC120* | 1.983929 | 0.000287 | *GDAP1* | 2.076791 | 9.19E-05 |
| *DOPEY1* | 1.981659 | 0.007387 | *RAB11FIP4* | 2.07652 | 0.000112 |
| *MARVELD3* | 1.975247 | 0.000196 | *FAM110C* | 2.052196 | 0.004114 |
| *EGFR* | 1.967765 | 0.000245 | *LOC100996579* | 2.051755 | 0.000741 |
| *SESN2* | 1.965898 | 0.000885 | *MAL2* | 2.046753 | 3.08E-05 |
| *ZDHHC21* | 1.959666 | 0.001265 | *PA2G4P4* | 2.044396 | 4.72E-05 |
| *CAPG* | 1.95551 | 0.001087 | *NMI* | 2.03458 | 0.000299 |
| *ARHGAP26* | 1.952025 | 0.000528 | *ARHGAP18* | 2.033987 | 3.29E-05 |
| *MIR622* | 1.950418 | 0.001603 | *SEMA3A* | 2.025694 | 8.35E-05 |
| *FAS* | 1.950198 | 0.000276 | *DCP1B* | 2.016693 | 0.000329 |
| *USP53* | 1.947449 | 0.003418 | *RNF148* | 2.013541 | 0.00019 |
| *PADI2* | 1.93524 | 0.001427 | *PARP12* | 2.00929 | 4.59E-05 |
| *COMMD3* | 1.933486 | 0.000399 | *MIR4653* | 2.008248 | 0.001078 |
| *L1CAM* | 1.931311 | 0.001473 | *STEAP2* | 2.002915 | 5.52E-05 |
| *MOXD1* | 1.930484 | 0.001817 | *PYROXD1* | 2.002438 | 0.000322 |
| *OR7E91P* | 1.929409 | 0.003684 | *EPB41L4A-AS1* | 2.002382 | 0.000459 |
| *CHMP4C* | 1.923079 | 0.000522 | *FAM169A* | 1.997853 | 0.000103 |
| *CGN* | 1.921349 | 0.000849 | *RAPGEF5* | 1.993533 | 0.00012 |
| *BAMBI* | 1.916297 | 0.010207 | *GAL* | 1.991434 | 0.000132 |
| *ARHGAP18* | 1.915002 | 0.000511 | *CDC42SE2* | 1.989618 | 0.000211 |
| *KDM7A* | 1.90987 | 0.000231 | *KRTAP2-3* | 1.988308 | 0.009574 |
| *PDE9A* | 1.907866 | 0.001139 | *BAZ2B* | 1.976986 | 0.00056 |
| *SERINC2* | 1.90224 | 0.000673 | *SNHG12* | 1.962824 | 0.000219 |
| *ICA1* | 1.897598 | 0.000222 | *GPR160* | 1.962458 | 0.001309 |
| *CTSO* | 1.896119 | 0.000619 | *LINC00702* | 1.962236 | 0.014589 |
| *TIFA* | 1.893407 | 0.00087 | *CBLC* | 1.956114 | 0.001548 |
| *PLEKHB1* | 1.89268 | 0.001878 | *CARS* | 1.955038 | 0.000124 |
| *CD40* | 1.88449 | 0.000799 | *LANCL2* | 1.946311 | 9.48E-05 |
| *LOXL1-AS1* | 1.883798 | 0.000754 | *CAMK1D* | 1.946289 | 0.007706 |
| *RASA1* | 1.881671 | 0.000595 | *KIF18A* | 1.93991 | 0.000233 |
| *MND1* | 1.879461 | 0.000764 | *MIR181A1HG* | 1.938212 | 0.001673 |
| *SH2D3A* | 1.875093 | 0.001369 | *INHBE* | 1.936015 | 0.003865 |
| *CFH* | 1.869968 | 0.003984 | *MMD* | 1.931019 | 0.000116 |
| *EZH2* | 1.867837 | 0.000857 | *PPP1R14C* | 1.928644 | 0.000309 |
| *LAT2* | 1.864395 | 0.001524 | *SYTL2* | 1.928574 | 0.000116 |
| *ATF3* | 1.864077 | 0.001556 | *ZNF585B* | 1.92336 | 6.92E-05 |
| *ZNF641* | 1.851072 | 0.002085 | *SNX24* | 1.920846 | 0.001612 |
| *EFNB2* | 1.844974 | 0.000424 | *SERINC2* | 1.920091 | 7.84E-05 |
| *HOOK1* | 1.842047 | 0.006982 | *CGN* | 1.916274 | 0.00037 |
| *ZNF280C* | 1.832419 | 0.001759 | *GBP1* | 1.890956 | 0.007421 |
| *GLIPR1* | 1.827602 | 0.00305 | *APOL6* | 1.88922 | 6.36E-05 |
| *FAAH2* | 1.826627 | 0.004953 | *TMEM54* | 1.886949 | 0.000219 |
| *GAL* | 1.824004 | 0.0008 | *RNF144B* | 1.882219 | 0.000821 |
| *IFIT2* | 1.82328 | 0.002154 | *ZFP36* | 1.882185 | 0.000242 |
| *KIAA0922* | 1.820359 | 0.000591 | *SPX* | 1.881196 | 0.010225 |
| *FRMD5* | 1.815521 | 0.000525 | *NABP1* | 1.878356 | 0.000114 |
| *ACTC1* | 1.814587 | 0.035173 | *ANKFN1* | 1.869385 | 0.016101 |
| *HOXB3* | 1.797909 | 0.0007 | *RYBP* | 1.867051 | 0.000106 |
| *CIRBP* | 1.797839 | 0.000686 | *EZH2* | 1.866185 | 0.000683 |
| *EPB41L4A-AS1* | 1.795875 | 0.000872 | *GRPEL2* | 1.862427 | 0.000185 |
| *SNORA47* | 1.791673 | 0.003095 | *TRIM38* | 1.85914 | 0.000781 |
| *ZC3H15* | 1.785987 | 0.002583 | *ARHGEF5* | 1.85251 | 0.000274 |
| *PTER* | 1.782596 | 0.001185 | *ZNF641* | 1.850701 | 0.000158 |
| *CARS* | 1.778308 | 0.000603 | *H1F0* | 1.846138 | 0.000115 |
| *STAP2* | 1.777461 | 0.000482 | *KDM7A* | 1.845062 | 0.000835 |
| *INADL* | 1.776759 | 0.000634 | *CYP2R1* | 1.842603 | 0.000144 |
| *TINAGL1* | 1.774637 | 0.003726 | *CALD1* | 1.83153 | 7.86E-05 |
| *HSH2D* | 1.774365 | 0.001434 | *ETV6* | 1.829605 | 0.000137 |
| *RNF148* | 1.773205 | 0.008245 | *CCDC120* | 1.829445 | 7.49E-05 |
| *MICAL2* | 1.766069 | 0.001285 | *SNORA47* | 1.827553 | 0.000477 |
| *PARP11* | 1.763569 | 0.000902 | *GSTK1* | 1.820305 | 0.000271 |
| *TUBE1* | 1.76181 | 0.007471 | *LAMC2* | 1.819534 | 0.000848 |
| *C10orf32* | 1.759796 | 0.003583 | *SLC41A2* | 1.815349 | 0.000438 |
| *LGALS8* | 1.756315 | 0.000522 | *LACC1* | 1.795476 | 0.000352 |
| *ARAP2* | 1.752643 | 0.007588 | *WARS* | 1.794687 | 0.00047 |
| *MARVELD2* | 1.745497 | 0.001203 | *CASP4* | 1.79428 | 0.001868 |
| *LOC647264* | 1.734542 | 0.003603 | *PABPC1L* | 1.788364 | 0.008358 |
| *ELL3* | 1.733466 | 0.000821 | *ADCK2* | 1.785655 | 0.000481 |
| *RAB11FIP4* | 1.728062 | 0.005442 | *LIPG* | 1.784918 | 0.001466 |
| *TIPARP* | 1.724583 | 0.000752 | *BTG3* | 1.782136 | 0.008511 |
| *MPZL3* | 1.723163 | 0.001392 | *LRBA* | 1.77775 | 0.000352 |
| *TRIB3* | 1.721384 | 0.001127 | *GLIPR1* | 1.775407 | 0.00034 |
| *FA2H* | 1.721325 | 0.013382 | *PRDM1* | 1.767247 | 0.000886 |
| *IL17RE* | 1.714178 | 0.002692 | *ARNT2* | 1.762545 | 0.026977 |
| *LSR* | 1.709149 | 0.002006 | *RSL24D1* | 1.761264 | 0.000124 |
| *OASL* | 1.70489 | 0.001432 | *DDX60* | 1.758622 | 0.012476 |
| *MNS1* | 1.701763 | 0.002475 | *AIM1* | 1.754828 | 0.000278 |
| *AIM1* | 1.698252 | 0.0008 | *HERC6* | 1.752092 | 0.000637 |
| *TNS4* | 1.693646 | 0.001012 | *BEST1* | 1.749603 | 0.009531 |
| *PABPC1L* | 1.691469 | 0.003657 | *EGFR* | 1.746408 | 0.003796 |
| *SKIL* | 1.690242 | 0.003269 | *ARHGAP10* | 1.743052 | 0.000489 |
| *TULP3* | 1.689678 | 0.000895 | *TMEM62* | 1.742072 | 0.000181 |
| *RAPGEF5* | 1.689548 | 0.011234 | *FBXL14* | 1.73791 | 0.000168 |
| *ZFP36* | 1.686771 | 0.002087 | *CLMP* | 1.733739 | 0.000116 |
| *HSD17B12* | 1.685669 | 0.000903 | *ZNHIT6* | 1.732797 | 0.001057 |
| *TMEM62* | 1.683673 | 0.001098 | *B4GALNT3* | 1.731911 | 0.000494 |
| *GSTK1* | 1.681743 | 0.001688 | *ZNF280C* | 1.727816 | 0.001159 |
| *USP16* | 1.679833 | 0.007471 | *PARP11* | 1.727138 | 0.002186 |
| *SNHG8* | 1.678295 | 0.00244 | *OSBPL10* | 1.72556 | 0.000141 |
| *TBC1D8B* | 1.671333 | 0.003001 | *EGLN3* | 1.725514 | 0.001584 |
| *DRAM1* | 1.670995 | 0.003385 | *CREM* | 1.720675 | 0.001561 |
| *SH3GL2* | 1.670193 | 0.007026 | *HOMER2* | 1.718493 | 0.000172 |
| *CDC42EP3* | 1.665927 | 0.001563 | *CAMSAP3* | 1.711674 | 0.000186 |
| *PLXNA2* | 1.66543 | 0.004506 | *CHAC1* | 1.710159 | 0.00809 |
| *SNORA33* | 1.664799 | 0.022888 | *SLIT2* | 1.709844 | 0.000302 |
| *PRDM1* | 1.659057 | 0.014371 | *L1CAM* | 1.703548 | 0.000288 |
| *CYP2R1* | 1.657223 | 0.001388 | *FAM160A1* | 1.699541 | 0.000477 |
| *ARHGEF5* | 1.652369 | 0.001753 | *GOLT1A* | 1.698269 | 0.006356 |
| *LOC647859* | 1.651717 | 0.000946 | *APOBEC3D* | 1.696114 | 0.033082 |
| *GLS2* | 1.648611 | 0.004059 | *FRMD4B* | 1.693305 | 0.000934 |
| *ETV6* | 1.648303 | 0.000983 | *CXCL2* | 1.692646 | 0.009937 |
| *DERA* | 1.647024 | 0.001628 | *SDCCAG8* | 1.690693 | 0.000501 |
| *CAMSAP3* | 1.645696 | 0.008297 | *DNAJC6* | 1.689234 | 0.000826 |
| *B3GALT5* | 1.642714 | 0.023037 | *RASA1* | 1.682401 | 0.000842 |
| *FDX1* | 1.638196 | 0.003889 | *DOPEY1* | 1.681655 | 0.000594 |
| *CCDC125* | 1.637418 | 0.005058 | *IDS* | 1.678544 | 0.000479 |
| *TMEM30B* | 1.636964 | 0.017287 | *TBC1D8B* | 1.677342 | 0.017555 |
| *PRR15* | 1.634174 | 0.003192 | *NAGS* | 1.677094 | 0.000391 |
| *NNMT* | 1.633301 | 0.001368 | *PCBD2* | 1.675707 | 0.001827 |
| *OSBPL10* | 1.630678 | 0.001804 | *MRPL35* | 1.671975 | 0.000334 |
| *RYBP* | 1.628568 | 0.003724 | *PLXNA2* | 1.671095 | 0.002524 |
| *FAM115C* | 1.62819 | 0.00557 | *MTUS1* | 1.669946 | 0.001436 |
| *C2orf15* | 1.627888 | 0.028631 | *HSH2D* | 1.667743 | 0.002821 |
| *MESDC1* | 1.626784 | 0.00115 | *DLG3* | 1.666955 | 0.000553 |
| *LAMA3* | 1.626293 | 0.001977 | *KIAA1147* | 1.663071 | 0.001142 |
| *BTG3* | 1.623603 | 0.002261 | *MME* | 1.661356 | 0.007846 |
| *CDC42SE2* | 1.615867 | 0.002748 | *CTSO* | 1.654981 | 0.033591 |
| *ZBED3* | 1.613101 | 0.003205 | *CGNL1* | 1.647129 | 0.001871 |
| *WWC1* | 1.610323 | 0.002019 | *LINC00673* | 1.643323 | 0.000386 |
| *ALOX12P2* | 1.609959 | 0.001948 | *AVEN* | 1.640594 | 0.001053 |
| *STEAP2* | 1.605669 | 0.012814 | *CDC42EP1* | 1.633935 | 0.000979 |
| *PCBD2* | 1.604662 | 0.011116 | *ELFN1-AS1* | 1.63312 | 0.019092 |
| *GPR160* | 1.604203 | 0.011116 | *ELL3* | 1.630523 | 0.001878 |
| *SDC4* | 1.602247 | 0.002342 | *HES1* | 1.628057 | 0.000441 |
| *SEMA3E* | 1.601924 | 0.007333 | *TIFA* | 1.62607 | 0.000968 |
| *LOC84214* | 1.601788 | 0.010156 | *XRCC4* | 1.624578 | 0.000992 |
| *KIF18A* | 1.599006 | 0.016476 | *REPS2* | 1.623565 | 0.000776 |
| *REPS2* | 1.597592 | 0.013426 | *CIRBP* | 1.618708 | 0.001612 |
| *STEAP4* | 1.595396 | 0.011116 | *NT5C3A* | 1.618211 | 0.004169 |
| *OGFRL1* | 1.593222 | 0.006759 | *IRF1* | 1.616353 | 0.004206 |
| *INHBE* | 1.591517 | 0.006494 | *SCAPER* | 1.613335 | 0.000877 |
| *C17orf51* | 1.584694 | 0.005068 | *DMXL1* | 1.6122 | 0.000398 |
| *ARHGAP44* | 1.583665 | 0.002468 | *RAB3B* | 1.611934 | 0.000571 |
| *SCAPER* | 1.577684 | 0.004137 | *ACTC1* | 1.610496 | 0.001724 |
| *ACTR3C* | 1.576147 | 0.005097 | *LOXL1-AS1* | 1.608719 | 0.000473 |
| *XRCC4* | 1.575491 | 0.008629 | *SMIM6* | 1.601336 | 0.016532 |
| *WARS* | 1.572166 | 0.00474 | *LOC84214* | 1.598747 | 0.008939 |
| *CDC42EP1* | 1.571387 | 0.003737 | *TULP3* | 1.584401 | 0.001111 |
| *HOMER2* | 1.570112 | 0.002019 | *HERPUD1* | 1.580596 | 0.003161 |
| *RNF144B* | 1.567105 | 0.002966 | *AFAP1-AS1* | 1.575348 | 0.01141 |
| *TMEM54* | 1.567008 | 0.012312 | *MESDC1* | 1.575005 | 0.000604 |
| *H1F0* | 1.566756 | 0.004309 | *USP53* | 1.569442 | 0.012345 |
| *LRIG1* | 1.566168 | 0.002044 | *STYXL1* | 1.569348 | 0.00145 |
| *MIR4653* | 1.564513 | 0.005188 | *PRICKLE1* | 1.567481 | 0.001718 |
| *FAM46C* | 1.56363 | 0.006616 | *DCUN1D3* | 1.565061 | 0.001931 |
| *ZSCAN12P1* | 1.562886 | 0.005995 | *FIGNL1* | 1.564728 | 0.008731 |
| *ESRP2* | 1.561393 | 0.006991 | *RPS6KA6* | 1.563973 | 0.010372 |
| *RTKN2* | 1.560671 | 0.029253 | *TSPAN31* | 1.563744 | 0.007099 |
| *NIPAL2* | 1.560346 | 0.00261 | *FAM115C* | 1.56221 | 0.002817 |
| *GTPBP10* | 1.559708 | 0.013249 | *HSD17B12* | 1.560412 | 0.000803 |
| *ALKBH3* | 1.554901 | 0.002971 | *SSBP1* | 1.559639 | 0.000743 |
| *GRAMD1B* | 1.554709 | 0.001861 | *MND1* | 1.557307 | 0.006303 |
| *ELF3* | 1.552987 | 0.041104 | *PLEKHA5* | 1.556134 | 0.002216 |
| *DCUN1D3* | 1.55248 | 0.006585 | *TMEM87B* | 1.554852 | 0.008959 |
| *FIGNL1* | 1.548728 | 0.012151 | *STEAP1* | 1.552695 | 0.031964 |
| *FBXL14* | 1.547666 | 0.002475 | *ELL2* | 1.552597 | 0.000595 |
| *ADCK2* | 1.544601 | 0.004872 | *PPP1R1C* | 1.546827 | 0.002411 |
| *RAB3B* | 1.542827 | 0.002915 | *LPXN* | 1.546328 | 0.001068 |
| *FAM66C* | 1.542422 | 0.044821 | *FAN1* | 1.542529 | 0.000483 |
| *CREM* | 1.538436 | 0.002222 | *TIPARP* | 1.535399 | 0.000637 |
| *ZNF774* | 1.538121 | 0.027329 | *B3GALT5* | 1.534057 | 0.002003 |
| *ZNF165* | 1.536708 | 0.016594 | *UPP1* | 1.533953 | 0.005791 |
| *LINC01239* | 1.532838 | 0.030443 | *IRAK2* | 1.533003 | 0.001679 |
| *ARFGAP2* | 1.529949 | 0.01888 | *COMMD3* | 1.531586 | 0.002768 |
| *ELFN1-AS1* | 1.526574 | 0.004468 | *USP16* | 1.531035 | 0.000934 |
| *LPCAT2* | 1.525533 | 0.013069 | *MOB3B* | 1.527276 | 0.005281 |
| *C12orf5* | 1.524693 | 0.01543 | *C12orf5* | 1.520711 | 0.002954 |
| *ARRDC4* | 1.523873 | 0.019025 | *SH3RF1* | 1.520615 | 0.00111 |
| *LANCL2* | 1.523595 | 0.006153 | *CCDC125* | 1.520529 | 0.00299 |
| *DMXL1* | 1.523054 | 0.044194 | *FDX1* | 1.519697 | 0.000706 |
| *SSBP1* | 1.521072 | 0.003588 | *DMKN* | 1.519662 | 0.000972 |
| *STYXL1* | 1.518871 | 0.013051 | *TJP2* | 1.518289 | 0.001056 |
| *FAN1* | 1.51818 | 0.012173 | *CCDC82* | 1.516761 | 0.00099 |
| *MYO5B* | 1.516778 | 0.012188 | *GALNT12* | 1.516709 | 0.003833 |
| *NT5C3A* | 1.515444 | 0.034258 | *ALKBH3* | 1.515424 | 0.001653 |
| *MLLT3* | 1.514963 | 0.005214 | *HDAC9* | 1.512157 | 0.001185 |
| *ZNHIT6* | 1.514621 | 0.010632 | *ERAP1* | 1.509513 | 0.012377 |
| *LINC01116* | 1.510779 | 0.005675 | *LAMA3* | 1.508246 | 0.00236 |
| *PRICKLE1* | 1.510387 | 0.004953 | *GRAMD1B* | 1.507596 | 0.002265 |
| *MRPL15* | 1.504777 | 0.003021 | *ZC3H15* | 1.506023 | 0.000934 |
| *C4orf32* | 1.503101 | 0.006481 | *IL7* | 1.505746 | 0.006553 |
| *RSL24D1* | 1.502593 | 0.008645 | *CAPG* | 1.502404 | 0.011925 |
| *DNAJC1* | 1.501294 | 0.015978 | *ARHGAP26* | 1.500956 | 0.004544 |
| **COMMON DOWN-REGULATED GENES in CZR vs. DR** | | | | | |
| **DU-145CZR vs. DU-145DR** | | | **PC-3CZR vs. PC-3DR** | | |
| **Gene**  **Symbol** | **FC** | **FDR** | **Gene Symbol** | **FC** | **FDR** |
| *DNAJC15* | -15.4709 | 7.86E-07 | *CPA4* | -4.95937 | 7.13E-06 |
| *IGFBP3* | -9.69303 | 7.99E-07 | *NOG* | -4.85958 | 4.44E-06 |
| *ID3* | -9.31009 | 1.14E-06 | *MYL9* | -4.30886 | 7.3E-07 |
| *SMAD4* | -6.83448 | 3.85E-07 | *NID2* | -4.21954 | 1.75E-06 |
| *FAT4* | -6.36717 | 3.85E-07 | *FGFR1* | -4.03621 | 2.24E-06 |
| *SLC47A1* | -5.8599 | 1.49E-06 | *CST1* | -3.96581 | 0.005222 |
| *CKB* | -4.97672 | 6.02E-06 | *SUSD2* | -3.81433 | 1.39E-06 |
| *TGFB1I1* | -4.834 | 0.000102 | *EFR3B* | -3.58425 | 1.86E-06 |
| *PTK7* | -4.64572 | 1.65E-06 | *PTPRG* | -3.57229 | 2.09E-06 |
| *NID2* | -4.49395 | 6.75E-06 | *ARHGAP23* | -3.55986 | 4.84E-06 |
| *SLCO4A1* | -4.04759 | 1.04E-05 | *LTBP1* | -3.45029 | 9.93E-07 |
| *ITGB3* | -3.9818 | 2.6E-06 | *ZNF667-AS1* | -3.2896 | 4.59E-06 |
| *LOC101559451* | -3.94032 | 2.99E-06 | *TCEA3* | -3.21088 | 2.27E-06 |
| *TENM3* | -3.79097 | 2.25E-06 | *MAP2K6* | -3.10646 | 6.22E-05 |
| *ST6GALNAC2* | -3.78959 | 8.64E-06 | *TUBB4A* | -3.04618 | 0.000148 |
| *CRIP1* | -3.67473 | 0.000224 | *CPM* | -2.91259 | 4.59E-06 |
| *CPA4* | -3.62189 | 1.09E-05 | *TXNDC16* | -2.88084 | 1.51E-05 |
| *NUTF2* | -3.32635 | 2.7E-05 | *MXRA7* | -2.86311 | 3.64E-06 |
| *LPCAT1* | -3.32385 | 4.91E-06 | *BMP6* | -2.85091 | 0.000251 |
| *ZNF844* | -3.28759 | 3.99E-05 | *HSPG2* | -2.83533 | 5.04E-06 |
| *GPR135* | -3.10943 | 4.2E-05 | *GPR137C* | -2.81834 | 1.67E-05 |
| *ARHGAP23* | -3.06314 | 2.88E-05 | *ZNF844* | -2.80243 | 0.000129 |
| *LOXL2* | -3.06095 | 1.2E-05 | *MN1* | -2.77199 | 1.22E-05 |
| *LOC100506100* | -3.05364 | 7.73E-05 | *TGFB1I1* | -2.7537 | 3.47E-05 |
| *PLAG1* | -3.01995 | 0.001809 | *CYBRD1* | -2.73559 | 2.05E-05 |
| *PRRX2* | -3.00494 | 6.94E-05 | *C5orf42* | -2.64532 | 5.35E-05 |
| *PIK3C3* | -2.90331 | 8.71E-06 | *HTRA1* | -2.62665 | 6.65E-06 |
| *RHOBTB3* | -2.87289 | 1.14E-05 | *EHD2* | -2.59463 | 0.006814 |
| *HIST1H2BC* | -2.8514 | 0.000383 | *FAXC* | -2.54942 | 1.29E-05 |
| *ANXA2R* | -2.78722 | 3.66E-05 | *CD22* | -2.52049 | 0.000361 |
| *LOC100996660* | -2.78139 | 1.33E-05 | *ABHD3* | -2.50902 | 9.66E-05 |
| *ANKRD20A5P* | -2.77931 | 0.00052 | *SCARNA17* | -2.50647 | 1.2E-05 |
| *ARHGAP24* | -2.75786 | 3.64E-05 | *LOXL2* | -2.47417 | 1.07E-05 |
| *MAP2K6* | -2.74054 | 7.8E-05 | *NUTF2* | -2.46941 | 0.003829 |
| *SCARNA17* | -2.72993 | 2.86E-05 | *ARHGAP24* | -2.44502 | 1.22E-05 |
| *MYL9* | -2.58248 | 3.64E-05 | *KDELC1* | -2.42785 | 3.57E-05 |
| *TTLL11-IT1* | -2.51495 | 0.000119 | *PIAS2* | -2.42676 | 2.24E-05 |
| *PTPRG* | -2.47799 | 9.64E-05 | *PINK1* | -2.41536 | 1.18E-05 |
| *EML1* | -2.47353 | 0.000205 | *GAB1* | -2.41512 | 2.74E-05 |
| *HTRA1* | -2.38938 | 0.000183 | *TRERF1* | -2.40149 | 1.42E-05 |
| *PTPRS* | -2.3745 | 5.23E-05 | *TENM3* | -2.39835 | 1.2E-05 |
| *FUT11* | -2.36645 | 0.000107 | *CKB* | -2.3547 | 3.56E-05 |
| *ZCCHC2* | -2.34552 | 4.86E-05 | *CITED4* | -2.33886 | 9.21E-06 |
| *BMP6* | -2.33674 | 8.36E-05 | *RHOBTB3* | -2.3318 | 2.06E-05 |
| *PLOD1* | -2.32449 | 0.000116 | *GSPT2* | -2.32428 | 0.000313 |
| *ZNF625* | -2.31551 | 0.000568 | *LEPRE1* | -2.27677 | 2.55E-05 |
| *GPR137C* | -2.29259 | 9.1E-05 | *CA5B* | -2.27009 | 3.59E-05 |
| *RTTN* | -2.22619 | 9.36E-05 | *LIFR* | -2.23421 | 1.37E-05 |
| *TCEA3* | -2.20749 | 0.000184 | *IGFBP3* | -2.20614 | 1.97E-05 |
| *OLFML2A* | -2.18357 | 0.000309 | *ATP2B4* | -2.19931 | 2.78E-05 |
| *QPCTL* | -2.17556 | 0.000673 | *CRIP1* | -2.18685 | 4.25E-05 |
| *ADNP2* | -2.1695 | 0.000158 | *LPCAT1* | -2.16817 | 1.92E-05 |
| *PINK1* | -2.16484 | 0.000333 | *SNORD58C* | -2.15192 | 0.000635 |
| *STXBP1* | -2.15874 | 0.000141 | *MMP16* | -2.15109 | 3.35E-05 |
| *CXXC1* | -2.15234 | 0.000216 | *PRTG* | -2.14788 | 0.000313 |
| *CCBL1* | -2.14004 | 0.000123 | *DFNB31* | -2.10323 | 6.52E-05 |
| *CLU* | -2.13384 | 0.000383 | *LOC101929643* | -2.09593 | 0.02782 |
| *EHMT2* | -2.12261 | 0.000368 | *SYT11* | -2.09039 | 0.000416 |
| *TRERF1* | -2.12176 | 0.000374 | *KDSR* | -2.07373 | 1.86E-05 |
| *ATP2B4* | -2.11099 | 0.000138 | *PARD6G* | -2.07238 | 7.1E-05 |
| *ATP9B* | -2.10732 | 0.000653 | *CLU* | -2.05779 | 2.55E-05 |
| *FBXO43* | -2.10666 | 0.000165 | *PLAG1* | -2.05116 | 0.000401 |
| *LEPRE1* | -2.09752 | 0.000559 | *PDXP* | -2.05076 | 5.1E-05 |
| *MXRA7* | -2.08501 | 0.000149 | *ANXA2R* | -2.04306 | 0.000635 |
| *TUBB4A* | -2.06824 | 0.000435 | *FAM210B* | -2.04006 | 4.09E-05 |
| *EPG5* | -2.04986 | 0.000211 | *KLF12* | -2.04004 | 3.58E-05 |
| *FXR1* | -2.0438 | 0.000436 | *PTK7* | -2.03878 | 6.92E-05 |
| *HSPA2* | -2.03987 | 0.000149 | *CAV1* | -2.03613 | 0.000264 |
| *FAM114A1* | -2.02817 | 0.000224 | *RTTN* | -2.02912 | 0.000188 |
| *LOC153684* | -2.02393 | 0.000642 | *TIMP2* | -2.02663 | 2.74E-05 |
| *EHD2* | -2.00391 | 0.000618 | *CCBL1* | -2.02536 | 2.55E-05 |
| *C18orf54* | -1.99354 | 0.002045 | *DLEU1* | -2.01435 | 6.92E-05 |
| *PARD6G* | -1.9906 | 0.001777 | *GPR135* | -2.00043 | 0.000308 |
| *TXNL4A* | -1.98702 | 0.000316 | *PLOD1* | -1.99181 | 6.1E-05 |
| *LOC100506844* | -1.97731 | 0.001479 | *BNC2* | -1.98071 | 6.56E-05 |
| *CAP2* | -1.97685 | 0.000195 | *ITGB3* | -1.96557 | 0.006549 |
| *CDR2L* | -1.96489 | 0.000397 | *ZNF667* | -1.96216 | 8.38E-05 |
| *GPR125* | -1.9613 | 0.000333 | *FUT11* | -1.95922 | 0.000501 |
| *PC* | -1.96057 | 0.001518 | *MURC* | -1.93491 | 0.000403 |
| *C5orf42* | -1.94704 | 0.005563 | *EHMT2* | -1.91033 | 7.77E-05 |
| *TXNDC16* | -1.94445 | 0.002799 | *ABTB1* | -1.90793 | 0.000309 |
| *TMCC1-AS1* | -1.93092 | 0.000478 | *DBP* | -1.90486 | 7.4E-05 |
| *SORBS3* | -1.9296 | 0.000658 | *SLC47A1* | -1.8997 | 0.000461 |
| *LY6G5B* | -1.92513 | 0.000771 | *ZCCHC2* | -1.89155 | 0.000131 |
| *CTGF* | -1.92148 | 0.000442 | *SCD5* | -1.87524 | 0.000758 |
| *TYMSOS* | -1.91784 | 0.000753 | *PRKCA* | -1.87423 | 0.000129 |
| *TIMM21* | -1.90604 | 0.000603 | *ZNF625* | -1.86314 | 0.000852 |
| *ZNF667-AS1* | -1.90449 | 0.00116 | *DNAJC15* | -1.86238 | 0.000482 |
| *DFNB31* | -1.90231 | 0.001266 | *FAT4* | -1.85162 | 0.000727 |
| *C8orf58* | -1.89703 | 0.012772 | *LOC153684* | -1.85002 | 0.000407 |
| *COLGALT1* | -1.89627 | 0.000854 | *RALGDS* | -1.84797 | 0.000465 |
| *CNTNAP3B* | -1.87671 | 0.000706 | *TIMM21* | -1.84773 | 0.000244 |
| *CPM* | -1.87477 | 0.000265 | *SNORD58A* | -1.84115 | 0.000321 |
| *PGAP1* | -1.8612 | 0.005311 | *ST6GALNAC2* | -1.84113 | 0.003973 |
| *RRAS* | -1.85432 | 0.002611 | *OLFML2A* | -1.84061 | 0.005273 |
| *WDR7* | -1.85049 | 0.002345 | *EPG5* | -1.80806 | 0.000107 |
| *PFAS* | -1.84635 | 0.001941 | *NID1* | -1.8054 | 9.43E-05 |
| *DLEU1* | -1.84503 | 0.001158 | *SLCO4A1* | -1.79796 | 0.000259 |
| *PTPRM* | -1.84341 | 0.000312 | *SMAD4* | -1.7863 | 0.000127 |
| *FGFR1* | -1.83987 | 0.000872 | *ANKRD20A5P* | -1.78427 | 0.000332 |
| *LIFR* | -1.82561 | 0.003164 | *CUTA* | -1.77283 | 0.000248 |
| *LTBP1* | -1.81744 | 0.002124 | *CACNA2D2* | -1.74279 | 0.000128 |
| *MIR27A* | -1.8158 | 0.00063 | *C18orf54* | -1.72723 | 0.001313 |
| *MIB1* | -1.80209 | 0.000965 | *CTGF* | -1.72323 | 0.00159 |
| *TIMP2* | -1.80143 | 0.000603 | *FAM69B* | -1.71333 | 0.000589 |
| *SYT11* | -1.80023 | 0.001125 | *NCAPD3* | -1.71098 | 0.002885 |
| *RALGDS* | -1.78745 | 0.002898 | *EML1* | -1.70877 | 0.0003 |
| *GSPT2* | -1.78296 | 0.01266 | *IER3IP1* | -1.70747 | 0.000376 |
| *CNPY3* | -1.77705 | 0.001714 | *GPR125* | -1.69397 | 0.000516 |
| *EFR3B* | -1.76135 | 0.006815 | *LOC101559451* | -1.68183 | 0.000446 |
| *ABHD3* | -1.75706 | 0.000822 | *PRRX2* | -1.6786 | 0.000386 |
| *NRM* | -1.75408 | 0.006711 | *LY6G5B* | -1.67693 | 0.001029 |
| *FAM102B* | -1.74097 | 0.009461 | *ZNF236* | -1.66816 | 0.000186 |
| *KIAA1211* | -1.73565 | 0.039031 | *STXBP1* | -1.66733 | 0.000204 |
| *MMP16* | -1.73312 | 0.000653 | *SCN1B* | -1.66235 | 0.000261 |
| *PLEKHG4* | -1.73283 | 0.004927 | *AXL* | -1.66168 | 0.000214 |
| *LOC101929643* | -1.72518 | 0.004506 | *ID3* | -1.66102 | 0.001837 |
| *ZNF32* | -1.72456 | 0.003109 | *NRM* | -1.65528 | 0.002744 |
| *NFATC3* | -1.7216 | 0.000906 | *MAP3K3* | -1.654 | 0.000349 |
| *ST3GAL2* | -1.71988 | 0.002234 | *LRP1* | -1.65058 | 0.001259 |
| *CAV1* | -1.70895 | 0.000622 | *ZNF331* | -1.64931 | 0.000645 |
| *IER3IP1* | -1.70817 | 0.004295 | *TTLL11-IT1* | -1.64336 | 0.000206 |
| *DOLK* | -1.7022 | 0.002484 | *ST3GAL2* | -1.64277 | 0.004393 |
| *CA5B* | -1.70026 | 0.001127 | *TXNL4A* | -1.63944 | 0.000519 |
| *CUTA* | -1.69952 | 0.00322 | *PC* | -1.63859 | 0.001042 |
| *DYM* | -1.6891 | 0.000715 | *SLC26A6* | -1.63696 | 0.000637 |
| *NID1* | -1.68868 | 0.000882 | *HIST1H2BC* | -1.63505 | 0.009023 |
| *PRKCA* | -1.68551 | 0.000802 | *DENND3* | -1.6348 | 0.009531 |
| *PDXP* | -1.67696 | 0.002371 | *ADNP2* | -1.63059 | 0.000379 |
| *SNORD58A* | -1.67053 | 0.003152 | *DOLK* | -1.62632 | 0.001005 |
| *SCN1B* | -1.66168 | 0.005925 | *LOC100506100* | -1.6228 | 0.002623 |
| *NOG* | -1.65307 | 0.002891 | *FAM114A1* | -1.62202 | 0.005591 |
| *CYBRD1* | -1.63763 | 0.002841 | *TMCC1-AS1* | -1.61838 | 0.000592 |
| *GAB1* | -1.63634 | 0.002689 | *LOC100506844* | -1.61651 | 0.003331 |
| *SAMD1* | -1.63521 | 0.0026 | *FXR1* | -1.60166 | 0.000279 |
| *SUSD2* | -1.63509 | 0.003044 | *ATP9B* | -1.59782 | 0.000654 |
| *SLC26A6* | -1.63474 | 0.007287 | *CXXC1* | -1.59631 | 0.00053 |
| *ZNF667* | -1.63082 | 0.003984 | *HSPA2* | -1.59297 | 0.000726 |
| *ABTB1* | -1.62362 | 0.001323 | *NFATC3* | -1.59219 | 0.000795 |
| *GRN* | -1.61889 | 0.004486 | *CAP2* | -1.59069 | 0.002308 |
| *SMAD9* | -1.61741 | 0.004103 | *PLXND1* | -1.58976 | 0.00033 |
| *LRP1* | -1.60827 | 0.001915 | *DIXDC1* | -1.58821 | 0.001843 |
| *CACNA2D2* | -1.60226 | 0.009128 | *MIR27A* | -1.58812 | 0.008585 |
| *MURC* | -1.5945 | 0.021378 | *PFAS* | -1.58728 | 0.006239 |
| *CST1* | -1.58578 | 0.023398 | *PLEKHG4* | -1.58389 | 0.00348 |
| *ATP5A1* | -1.57357 | 0.00152 | *ATP5A1* | -1.58259 | 0.001022 |
| *ARPC5* | -1.56322 | 0.001948 | *SMAD9* | -1.5767 | 0.000404 |
| *DBP* | -1.56247 | 0.012565 | *FBXO43* | -1.57382 | 0.007342 |
| *ZNF331* | -1.56167 | 0.003013 | *DYM* | -1.57004 | 0.000776 |
| *AXL* | -1.5579 | 0.002556 | *CNTNAP3B* | -1.56778 | 0.008959 |
| *FAXC* | -1.55508 | 0.016193 | *WDR7* | -1.56606 | 0.000487 |
| *CITED4* | -1.55503 | 0.017116 | *PTPRM* | -1.56476 | 0.010189 |
| *SNORD58C* | -1.55219 | 0.026856 | *PTPRS* | -1.5608 | 0.000438 |
| *SCD5* | -1.55197 | 0.003504 | *KIAA1211* | -1.56049 | 0.000864 |
| *NCAPD3* | -1.54572 | 0.002602 | *FAM102B* | -1.55748 | 0.000887 |
| *PRTG* | -1.53927 | 0.002432 | *LOC100996660* | -1.55716 | 0.012812 |
| *DENND3* | -1.53746 | 0.004209 | *PIK3C3* | -1.55291 | 0.001605 |
| *FAM210B* | -1.534 | 0.003021 | *ZNF32* | -1.55095 | 0.014516 |
| *LRRC34* | -1.53083 | 0.004295 | *LRRC34* | -1.54483 | 0.004644 |
| *SNORA37* | -1.5291 | 0.007027 | *COLGALT1* | -1.53926 | 0.019643 |
| *PIAS2* | -1.52893 | 0.006146 | *TYMSOS* | -1.53248 | 0.017596 |
| *MN1* | -1.52634 | 0.00583 | *C8orf58* | -1.5296 | 0.002319 |
| *BNC2* | -1.52174 | 0.018941 | *GRN* | -1.5288 | 0.001088 |
| *KLF12* | -1.5192 | 0.049695 | *PPP2R2C* | -1.52191 | 0.000604 |
| *PPP2R2C* | -1.51887 | 0.002727 | *CDR2L* | -1.51938 | 0.001668 |
| *DIXDC1* | -1.51469 | 0.003736 | *CNPY3* | -1.51781 | 0.000552 |
| *LOC730101* | -1.51364 | 0.007861 | *SNORA37* | -1.51655 | 0.002511 |
| *ZNF236* | -1.51301 | 0.002915 | *MIB1* | -1.51322 | 0.005865 |
| *KDSR* | -1.51166 | 0.005933 | *LOC730101* | -1.51108 | 0.004562 |
| *KDELC1* | -1.50768 | 0.023785 | *RRAS* | -1.51058 | 0.003262 |
| *HSPG2* | -1.5065 | 0.004802 | *SAMD1* | -1.50916 | 0.007494 |
| *FAM69B* | -1.50631 | 0.027337 | *SORBS3* | -1.50528 | 0.003494 |
| *CD22* | -1.50446 | 0.003416 | *QPCTL* | -1.5045 | 0.001755 |
| *MAP3K3* | -1.50256 | 0.006815 | *ARPC5* | -1.50301 | 0.002319 |
| *PLXND1* | -1.50047 | 0.026909 | PGAP1 | -1.50095 | 0.018014 |

**Table S5.** *In silico* analysis from public gene expression datasets. *In silico* analysis of alterations’ frequency of cell plasticity related genes in prostate cancer tumors through cBio Cancer Genomics Portal (http://cbioportal.org) platform. Mut: Mutation, Del: Deep Deletion.

|  | | ***ESRP1*** | | | ***ZEB1*** | | | ***CDH1*** | | | ***AXL*** | | |
| --- | --- | --- | --- | --- | --- | --- | --- | --- | --- | --- | --- | --- | --- |
| **Study** | **Type** | **Amp** | **Mut** | **Del** | **Amp** | **Mut** | **Del** | **Amp** | **Mut** | **Del** | **Amp** | **Mut** | **Del** |
| NEPC (1) | Neuroendocrine | 41.2%  (47/114) | 0.9% | -- | 7.9%  (9/114) | -- | -- | 3.5%  (4/114) | -- | 0.9% | 10.5%  (12/114) | 0.9% | -- |
| MICH (2) | Metastatic | 24.6%  (15/61) | -- | -- | -- | -- | 3.3%  (2/61) | 1.6% | 1.6% | 3.3%  (2/61) | -- | 1.6%  (1/61) | 1.6% |
| FHCRC (3) | Metastatic | 17%  (30/176) | -- | 0.6% | -- | 2.8%  (5/176) | 0.6% | 1.7% | -- | 2.8%  (5/176) | -- | 1.7%  (3/176) | 1.7%  (3/176) |
| SU2C (4) | Metastatic | 14%  (21/150) | 2.7% | -- | 1.3%  (2/150) | 1.3% | 1.3% | -- | 1.3%  (2/150) | 1.3% | -- | 1.3%  (2/150) | 0.7% |
| TCGA (5) | Primary tumor | 5.7%  (19/333) | 0.3% | 0.3% | -- | 0.6% | 1.2%  (4/333) | 0.3% | 0.6% | 4.5%  (15/333) | -- | 0.3%  (1/333) | 0.3% |
| MSKCC (6) | Primary tumor | 5.7%  (11/194) | -- | -- | -- | -- | 0.5%  (1/194) | 0.5%  (1/194) | 0.5% | 0.5% | -- | 1%  (2/194) | -- |
| Broad/Cornell (7) | Primary tumor | 5.3%  (3/57) | -- | -- | -- | -- | -- | -- | -- | -- | -- | -- | -- |

1. Beltran H, Prandi D, Mosquera JM, Benelli M, Puca L, Cyrta J, Marotz C, Giannopoulou E, Chakravarthi BVSK, Varambally S, et al. Divergent clonal evolution of castration-resistant neuroendocrine prostate cancer. *Nat Med* (2016) **22**:298–305. doi:10.1038/nm.4045

2. Grasso CS, Wu Y-M, Robinson DR, Cao X, Dhanasekaran SM, Khan AP, Quist MJ, Jing X, Lonigro RJ, Brenner JC, et al. The mutational landscape of lethal castration-resistant prostate cancer. *Nature* (2012) **487**:239–243. doi:10.1038/nature11125

3. Kumar A, Coleman I, Morrissey C, Zhang X, True LD, Gulati R, Etzioni R, Bolouri H, Montgomery B, White T, et al. Substantial interindividual and limited intraindividual genomic diversity among tumors from men with metastatic prostate cancer. *Nat Med* (2016) **22**:369–378. doi:10.1038/nm.4053

4. Robinson D, Van Allen EM, Wu Y-M, Schultz N, Lonigro RJ, Mosquera J-M, Montgomery B, Taplin M-E, Pritchard CC, Attard G, et al. Integrative clinical genomics of advanced prostate cancer. *Cell* (2015) **161**:1215–1228. doi:10.1016/j.cell.2015.05.001

5. Cancer Genome Atlas Research Network. The Molecular Taxonomy of Primary Prostate Cancer. *Cell* (2015) **163**:1011–1025. doi:10.1016/j.cell.2015.10.025

6. Taylor BS, Schultz N, Hieronymus H, Gopalan A, Xiao Y, Carver BS, Arora VK, Kaushik P, Cerami E, Reva B, et al. Integrative genomic profiling of human prostate cancer. *Cancer Cell* (2010) **18**:11–22. doi:10.1016/j.ccr.2010.05.026

7. Baca SC, Prandi D, Lawrence MS, Mosquera JM, Romanel A, Drier Y, Park K, Kitabayashi N, MacDonald TY, Ghandi M, et al. Punctuated evolution of prostate cancer genomes. *Cell* (2013) **153**:666–677. doi:10.1016/j.cell.2013.03.021

**Table S6.** Univariate and multivariate Cox model for PSA progression-free survival (PSA-PFS) in tumor samples. Univariate and multivariate Cox model for PSA-PFS in tumor samples adjusted for clinically significant variables (*P*<0.1) in univariate analysis (marked in bold). Gene expression was measured by qRT-PCR. *variables considered dichotomous; **variables considered continuous. ECOG: Eastern Cooperative Oncology Group; LDH: lactate dehydrogenase; Hb: haemoglobin concentration; PSA: prostate-specific antigen; AP: alkaline phosphatase; HR: hazard ratio; CI: confidence interval.

| **DOCETAXEL (PSA-PFS)** | **Univariate** | | | | **Multivariate** | | | |
| --- | --- | --- | --- | --- | --- | --- | --- | --- |
| **Variable** | HR | 95% CI | | *P*-value | HR | 95% CI | | *P*-value |
| Stage at diagnosis* | 0.958 | 0.587 | 1.564 | 0.865 | - | - | - | - |
| Gleason at diagnosis* | 0.842 | 0.529 | 1.340 | 0.469 | - | - | - | - |
| ECOG* | 1.583 | 0.848 | 2.954 | 0.149 | - | - | - | - |
| LDH** | 1.000 | 0.999 | 1.002 | 0.467 | - | - | - | - |
| Hb** | 1.000 | 0.989 | 1.011 | 0.968 | - | - | - | - |
| PSA** | 0.999 | 0.998 | 1.001 | 0.415 | - | - | - | - |
| AP** | 1.000 | 1.000 | 1.001 | 0.199 | - | - | - | - |
| Visceral metastases* | 1.020 | 0.619 | 1.681 | 0.939 | - | - | - | - |
| Bone metastases* | 1.469 | 0.635 | 3.398 | 0.369 | - | - | - | - |
| *ESRP1* expression* | **0.370** | **0.219** | **0.627** | **0.000** | **0.343** | **0.200** | **0.589** | **0.000** |
| *CHGA* expression* | **1.531** | **0.977** | **2.400** | **0.063** | 1.490 | 0.945 | 2.351 | 0.086 |
| *SYP* expression* | **1.647** | **1.042** | **2.603** | **0.033** | **2.051** | **1.276** | **3.298** | **0.003** |
| **CABAZITAXEL (PSA-PFS)** | **Univariate** | | | | **Multivariate** | | | |
| **Variable** | HR | 95% CI | | *P*-value | HR | 95% CI | | *P*-value |
| Stage at diagnosis* | 0.908 | 0.443 | 1.862 | 0.793 | - | - | - | - |
| Gleason at diagnosis* | 1.047 | 0.472 | 2.322 | 0.910 | - | - | - | - |
| ECOG* | 2.820 | 0.380 | 20.922 | 0.311 | - | - | - | - |
| LDH** | **1.001** | **1.000** | **1.002** | **0.011** | **1.002** | **1.001** | **1.003** | **0.005** |
| Hb** | 0.993 | 0.976 | 1.012 | 0.475 | - | - | - | - |
| PSA** | 1.000 | 0.999 | 1.001 | 0.704 | - | - | - | - |
| AP** | 1.001 | 0.999 | 1.002 | 0.573 | - | - | - | - |
| Visceral metastases* | 0.788 | 0.388 | 1.597 | 0.508 | - | - | - | - |
| Bone metastases* | 0.543 | 0.161 | 1.829 | 0.324 | - | - | - | - |
| *ESRP1* expression* | **2.202** | **1.046** | **4.635** | **0.038** | **3.085** | **1.048** | **9.086** | **0.041** |
| *ZEB1* expression* | **0.412** | **0.203** | **0.834** | **0.014** | 0.456 | 0.172 | 1.209 | 0.115 |
| *AXL* expression* | **0.355** | **0.165** | **0.765** | **0.008** | 1.447 | 0.426 | 4.914 | 0.554 |
| *AURKA* expression* | **1.903** | **0.945** | **3.834** | **0.072** | 0.935 | 0.336 | 2.601 | 0.898 |

**Table S7.** Univariate and multivariate Cox model for radiologic-PFS (RX-PFS) in tumor samples. Univariate and multivariate Cox model for RX-PFS in tumor samples adjusted for clinically significant variables (*P*<0.1) in univariate analysis (marked in bold). Gene expression was measured by qRT-PCR. *variables considered dichotomous; **variables considered continuous. ECOG: Eastern Cooperative Oncology Group; LDH: lactate dehydrogenase; Hb: haemoglobin concentration; PSA: prostate-specific antigen; AP: alkaline phosphatase; HR: hazard ratio; CI: confidence interval.

| **DOCETAXEL (RX-PFS)** | **Univariate** | | | | **Multivariate** | | | |
| --- | --- | --- | --- | --- | --- | --- | --- | --- |
| **Variable** | HR | 95% CI | | *P*-value | HR | 95% CI | | *P*-value |
| Stage at diagnosis* | 0.870 | 0.510 | 1.484 | 0.609 | - | - | - | - |
| Gleason at diagnosis* | 1.119 | 0.657 | 1.905 | 0.680 | - | - | - | - |
| ECOG* | **1.907** | **0.900** | **4.041** | **0.092** | **2.917** | **1.312** | **6.483** | **0.009** |
| LDH** | 1.001 | 0.999 | 1.002 | 0.393 | - | - | - | - |
| Hb** | 1.001 | 0.988 | 1.014 | 0.928 | - | - | - | - |
| PSA** | 0.999 | 0.997 | 1.000 | 0.182 | - | - | - | - |
| AP** | 1.000 | 1.000 | 1.001 | 0.456 | - | - | - | - |
| Visceral metastases* | 0.985 | 0.568 | 1.707 | 0.956 | - | - | - | - |
| Bone metastases* | 0.962 | 0.414 | 2.235 | 0.929 | - | - | - | - |
| *ESRP1* expression* | **0.479** | **0.278** | **0.827** | **0.008** | **0.327** | **0.172** | **0.620** | **0.001** |
| *CDH2* expression* | **1.838** | **1.059** | **3.188** | **0.030** | **2.226** | **1.167** | **4.248** | **0.015** |
| *SYP* expression* | **1.601** | **0.945** | **2.712** | **0.080** | **2.153** | **1.125** | **4.120** | **0.021** |

**Table S8.** Univariate and multivariate Cox model for overall survival (OS) in tumor samples. Univariate and multivariate Cox model for OS in tumor samples adjusted for clinically significant variables (*P*<0.1) in univariate analysis (marked in bold). Gene expression was measured by qRT-PCR. *variables considered dichotomous; **variables considered continuous. ECOG: Eastern Cooperative Oncology Group; LDH: lactate dehydrogenase; Hb: haemoglobin concentration; PSA: prostate-specific antigen; AP: alkaline phosphatase; HR: hazard ratio; CI: confidence interval.

| **DOCETAXEL (OS)** | **Univariate** | | | | **Multivariate** | | | |
| --- | --- | --- | --- | --- | --- | --- | --- | --- |
| **Variable** | HR | 95% CI | | *P*-value | HR | 95% CI | | *P*-value |
| Stage at diagnosis* | 0.959 | 0.611 | 1.506 | 0.857 | - | - | - | - |
| Gleason at diagnosis* | 1.242 | 0.803 | 1.920 | 0.330 | - | - | - | - |
| ECOG* | **2.224** | **1.220** | **4.052** | **0.009** | **2.384** | **1.006** | **5.649** | **0.048** |
| LDH** | **1.002** | **1.000** | **1.003** | **0.007** | 1.001 | 0.999 | 1.002 | 0.376 |
| Hb** | **0.978** | **0.967** | **0.988** | **0.000** | 0.990 | 0.974 | 1.006 | 0.205 |
| PSA** | 1.001 | 1.000 | 1.002 | 0.160 | - | - | - | - |
| AP** | **1.000** | **1.000** | **1.001** | **0.082** | 1.001 | 1.000 | 1.002 | 0.063 |
| Visceral metastases* | 0.954 | 0.607 | 1.501 | 0.839 | - | - | - | - |
| Bone metastases* | 1.769 | 0.831 | 3.765 | 0.139 | - | - | - | - |
| *CDH1* expression* | **0.636** | **0.390** | **1.036** | **0.069** | 0.626 | 0.267 | 1.471 | 0.283 |
| *VIM* expression* | **0.527** | **0.333** | **0.836** | **0.006** | 1.088 | 0.508 | 2.330 | 0.828 |
| *ZEB1* expression* | **0.612** | **0.397** | **0.945** | **0.027** | 0.768 | 0.353 | 1.671 | 0.505 |
| *CD44* expression* | **0.578** | **0.362** | **0.922** | **0.021** | 0.763 | 0.367 | 1.584 | 0.468 |
| AURKA expression* | **1.416** | **0.940** | **2.133** | **0.096** | 1.151 | 0.605 | 2.191 | 0.668 |
| *MYCN* expression* | **1.483** | **0.945** | **2.329** | **0.087** | 1.408 | 0.774 | 2.561 | 0.262 |
| *SYP* expression* | **1.506** | **0.961** | **2.361** | **0.074** | 1.704 | 0.896 | 3.242 | 0.104 |
| *EZH2* expression* | **1.775** | **1.129** | **2.792** | **0.013** | 1.078 | 0.512 | 2.268 | 0.844 |
| **CABAZITAXEL (OS)** | **Univariate** | | | | **Multivariate** | | | |
| **Variable** | HR | 95% CI | | *P*-value | HR | 95% CI | | *P*-value |
| Stage at diagnosis* | 0.981 | 0.483 | 1.991 | 0.957 | - | - | - | - |
| Gleason at diagnosis* | 0.932 | 0.417 | 2.084 | 0.864 | - | - | - | - |
| ECOG* | 1.014 | 0.240 | 4.283 | 0.985 | - | - | - | - |
| LDH** | **1.003** | **1.001** | **1.004** | **0.001** | **1.003** | **1.001** | **1.005** | **0.003** |
| Hb** | **0.979** | **0.963** | **0.996** | **0.014** | 1.002 | 0.977 | 1.027 | 0.886 |
| PSA** | 1.000 | 0.999 | 1.001 | 0.518 | - | - | - | - |
| AP** | 1.000 | 0.998 | 1.002 | 0.796 | - | - | - | - |
| Visceral metastases* | 0.708 | 0.358 | 1.401 | 0.321 | - | - | - | - |
| Bone metastases* | 1.849 | 0.554 | 6.172 | 0.317 | - | - | - | - |
| *CDH1* expression* | **0.509** | **0.254** | **1.020** | **0.057** | 1.271 | 0.446 | 3.623 | 0.653 |
| *VIM* expression* | **0.497** | **0.253** | **0.974** | **0.042** | 0.786 | 0.27 | 2.286 | 0.658 |
| *AXL* expression* | **0.376** | **0.155** | **0.913** | **0.031** | 1.385 | 0.349 | 5.488 | 0.643 |
| *AURKA* expression* | **1.865** | **0.960** | **3.624** | **0.066** | 1.076 | 0.442 | 2.622 | 0.871 |
| *SYP* expression* | **3.702** | **1.686** | **8.125** | **0.001** | **5.257** | **1.798** | **15.365** | **0.002** |
